# New Phenylspirodrimanes from the Sponge-Associated Fungus *Stachybotrys chartarum* MUT 3308

**DOI:** 10.3390/md21030135

**Published:** 2023-02-21

**Authors:** Marie Dayras, Estelle Sfecci, Elena Bovio, Olivia Rastoin, Maeva Dufies, Fabien Fontaine-Vive, Elisabeth Taffin-de-Givenchy, Thierry Lacour, Gilles Pages, Giovanna Cristina Varese, Mohamed Mehiri

**Affiliations:** 1 Marine Natural Products Team, Institut de Chimie de Nice, Université Côte d’Azur, CNRS UMR 7272, 06108 Nice, France; 2 Centre Scientifique de Monaco, LIA ROPSE, Laboratoire International Associé, Université Côte d’Azur, 06108 Nice, France; 3 Mycotheca Universitatis Taurinensis, Department of Life Sciences and Systems Biology, University of Turin, Viale Mattioli 25, 10125 Turin, Italy; 4 UMR Institut Sophia Agrobiotech, INRAE, CNRS, UCA, 400 routes des Chappes, 06903 Sophia Antipolis, France; 5 Centre Antoine Lacassagne, Institute for Research on Cancer and Aging of Nice, Université Côte d’Azur, CNRS UMR 7284, INSERM U1081, 06189 Nice, France; 6 Parc d’Activités Arôma Grasse/Immeuble Grasse Biotech, 45 boulevard Marcel Pagnol, 06130 Grasse, France; 7 Department of Biomedical, Centre Scientifique de Monaco, 98000 Monaco, Monaco

**Keywords:** marine fungus, *Stachybotrys chartarum*, isolation, phenylspirodrimanes, cytotoxicity

## Abstract

Two phenylspirodrimanes, never isolated before, stachybotrin J (**1**) and new stachybocin G (*epi*-stachybocin A) (**2**), along with the already reported stachybotrin I (**3**), stachybotrin H (**4**), stachybotrylactam (**5**), stachybotrylactam acetate (**6**), 2*α*-acetoxystachybotrylactam acetate (**7**), stachybotramide (**8**), chartarlactam B (**9**), and F1839-J (**10**) were isolated from the sponge-associated fungus *Stachybotrys chartarum* MUT 3308. Their structures were established based on extensive spectrometric (HRMS) and spectroscopic (1D and 2D NMR) analyses. Absolute configurations of the stereogenic centers of stachybotrin J (**1**), stachybocin G (**2**), and stachybotrin I (**3**), were determined by comparison of their experimental circular dichroism (CD) spectra with their time-dependent density functional theory (TD-DFT) circular dichroism (ECD) spectra. The putative structures of seventeen additional phenylspirodrimanes were proposed by analysis of their respective MS/MS spectra through a Feature-Based Molecular Networking approach. All the isolated compounds were evaluated for their cytotoxicity against five aggressive cancer cell lines (MP41, 786, 786R, CAL33, and CAL33RR), notably including two resistant human cancer cell lines (786R, CAL33RR), and compounds **5**, **6,** and **7** exhibited cytotoxicity with IC_50_ values in the range of 0.3−2.2 µM.

## 1. Introduction

Fungi belonging to the genus *Stachybotrys* produce a broad range of mycotoxins classified into three structural groups: macrocyclic trichothecenes (MT), atranones, and phenylspirodrimanes (PSDs) [1,2]. Monomeric and dimeric PSDs represent the largest group with over 80 meroterpenoids featuring an unusual PSD skeleton bearing various structural modifications. PSDs can be further divided into three main classes: tetracyclic aromatic sesquiterpenoids with alcohol and/or aldehyde functionalities, such as stachybotrydial [3,4], pentacyclic aromatic sesquiterpenoids, such as stachybotrylactam [5,6] and stachybotrylactone [7,8,9,10,11], and stachyflin and its derivatives which present a pentacyclic moiety with a *cis*-fused decalin [12,13]. Monomeric PSDs have been reported to exhibit a wide range of pharmacological activities such as antiosteoporosis properties [14], the ability to inhibit immune-complex disease [7,15], tyrosine kinase receptors [5], and antihyperlipidemic effects [16]. On the other side, dimers showed different biological activities compared to monomeric PSDs, such as antibacterial activities, potential ET receptor antagonists, and neuroprotective, anti-inflammatory, and antitumor activities [17,18,19,20]. In the literature, most PSDs were reported to exhibit weak (IC_50_ 18.4–24.7 μM) or no cytotoxicity [21,22]. To the best of our knowledge, PSDs have never been evaluated for their cytotoxicity against resistant cancer lines.

Drug resistance is currently a major problem in several cancers such as UM (uveal melanoma), RCC (renal cell carcinoma), and HNSCC (head and neck squamous cell carcinoma). Although the 1980s was the decade of general radio-chemotherapy with the use of very toxic antitumor drugs and radiation procedures that resulted in numerous side effects, the 2000s was the decade of targeted therapies due to the development of treatments that specifically target driving mutations [23,24,25]. Despite the improvements, many of the patients were not cured and suffered relapses. During the 2010s, the strategies focused on damaging the microenvironment, particularly blood vessels, in colon, breast, lung, and kidney cancers [26]. However, the effect of anti-angiogenic drugs was short-lived, and relapses were inevitable. The 2020s is definitely the decade of immunotherapies improving patient survival, but only in 20% of patients with various cancers such as UM, RCC, and HNSCC [27,28,29,30]. Therefore, to further improve current treatments, the introduction of new therapies is urgently needed. These breakthrough treatments can improve the quality of life or survival of many patients. The discovery of these new drugs from natural products is one of the most important tasks in medicinal chemistry.

**Figure 1:**
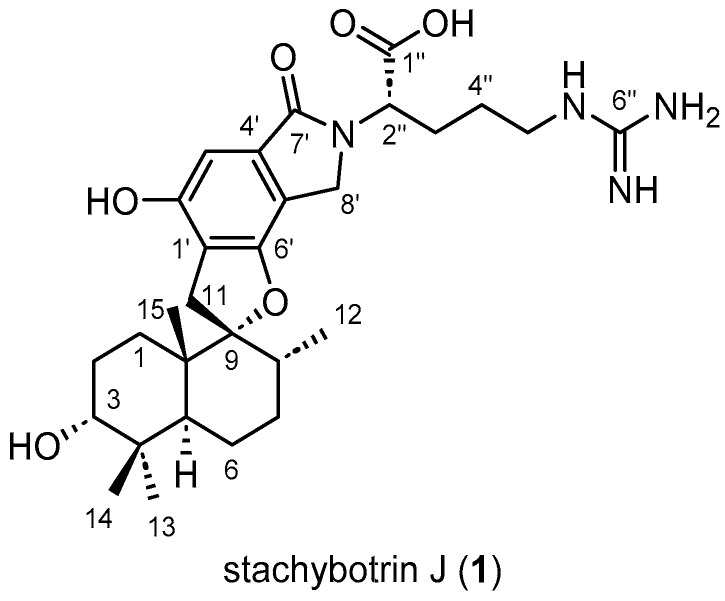

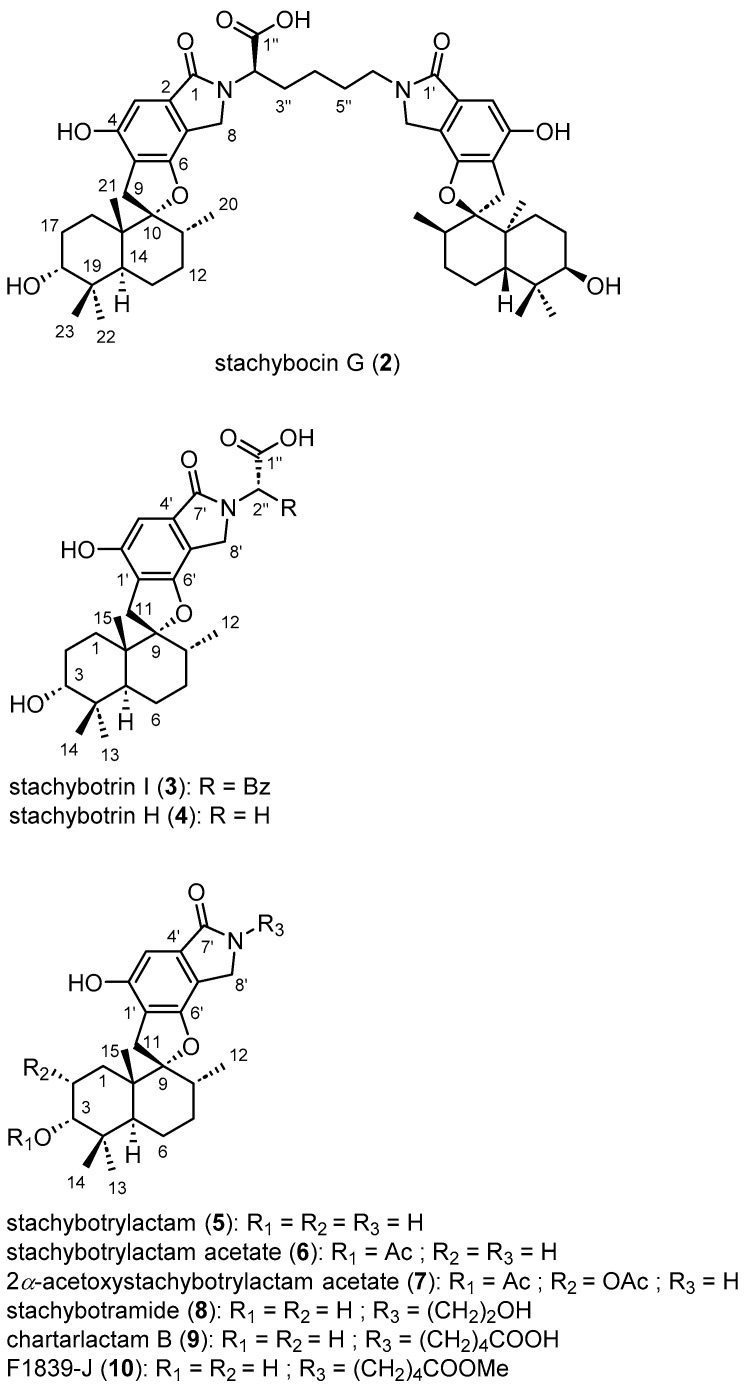
Chemical structure of the isolated compounds **1**–**10** (represented in their neutral form).

In this context, we were interested by PSDs produced by the fungus *Stachybotrys chartarum* MUT 3308, previously isolated from the Mediterranean sponge *Aplysina cavernicola* [31] and cultivated in solid and liquid media. Herein, we report the isolation of two PSDs, never isolated before, stachybotrin J (**1**) and new stachybocin G (*epi*-stachybocin A) (**2**), along with the already reported stachybotrin I (**3**), stachybotrin H (**4**), stachybotrylactam (**5**), stachybotrylactam acetate (**6**), 2*α*-acetoxystachybotrylactam acetate (**7**), stachybotramide (**8**), chartarlactam B (**9**), and F1839-J (**10**) (Figure 1). Moreover, structural hypotheses for seventeen additional PSD analogs have also been proposed using a Feature-Based Molecular Networking (FBMN) approach. All the isolated compounds were evaluated for their cytotoxic activities against several aggressive human cancer cell lines, notably including two resistant human cancer cell lines.

## 2. Results and Discussion

### 2.1. Structure Elucidation

*Stachybotrys chartarum* MUT 3308 was cultivated, in solid and liquid conditions, using PDA as a medium. For each culture condition, all the fungal material and the medium were extracted with an appropriate mixture of solvents and fractionated by reverse-phase or normal-phase chromatography. The most interesting fractions, based on the HPLC-PDA-ELSD and UHPLC-HRMS/MS metabolic profiles, were purified by RP HPLC to yield, in total, pure compounds **1** (2.1 mg), **2** (3.5 mg), **3** (3.1 mg), **4** (1.2 mg), **5** (9.4 mg), **6** (2.4 mg), **7** (2.3 mg), **8** (0.6 mg), **9** (2.1 mg), and **10** (1.3 mg), as white amorphous solids.

**Figure 2:**
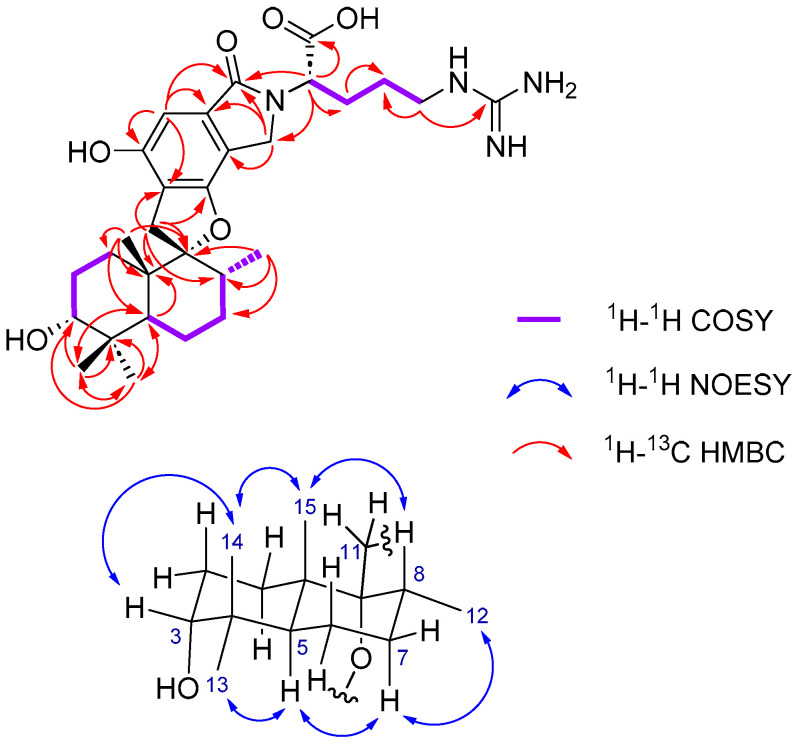
Key 2D NMR correlations for stachybotrin J (**1**).

The molecular formula of compound **1**, C_29_H_42_N_4_O_6_, was deduced from the HRESI(+)MS analysis which showed a pseudo-molecular ion peak at *m/z* 543.3171 [M + H]^+^ (543.3177 calc. C_29_H_43_N_4_O_6_^+^, 11 degrees of unsaturation). Compound **1** showed ^1^H and ^13^C NMR chemical shifts very close to those of stachybotrylactam (**5**) [$\bar{X}$(|Δ*δ*_H(**5**-**1**)_|) = 0.03 ppm and *s* = Δ*δ*_H(**5**-**1**)_ = 0.07 ppm, $\bar{X}$(|Δ*δ*_C(**5**-**1**)_|) = 0.3 ppm and *s* = Δ*δ*_C(**5**-**1**)_ = 0.8 ppm] which allowed us to deduce that their PSD moieties share the same absolute configuration: 3*R*, 5*S*, 8*R*, 9*R*, and 10*S*. The ^1^H and ^13^C NMR spectra of **1** also featured one methine [*δ*_H_ 4,76 (m, 1H, H-2″)], three methylenes [*δ*_H_ 3.24 (m, 2H, H-5″), 2.15 (m, 1H, H-3a″), 1.91 (m, 1H, H-3b″), 1.56 (m, 2H, H-4″), and *δ*_C_ 57.6 (C-2″), 41.9 (C-5″), 28.8 (C-3″), 27.2 (C-4″)], a guanidinium carbon [*δ*_C_ 158.6 (C-6″)], and a carboxylic acid carbon (*δ*_C_ 170.3, C-1″), characteristic of an arginine residue connected to the nitrogen, which was further confirmed by key H-2″/H-3″, H-3″/ H-4″, and H-4″/H-5″ COSY correlations but also key H-2″/C-7′, H-2″/C-8′, H-2″/C-1″, H-2″/C-3″, H-3″/C-4″, H-5″/C-4″, and H-5″/C-6″ HMBC correlations (Table 1, Figure 2, Table S1).

All this data allowed us to identify compound **1** as a PSD derivative never isolated before, named stachybotrin J (**1**). Although this compound was semi-synthesized by Steinert et al. in 2022, the absolute configuration of the stereogenic center of the arginine residue was not determined [32].

**Figure 3:**
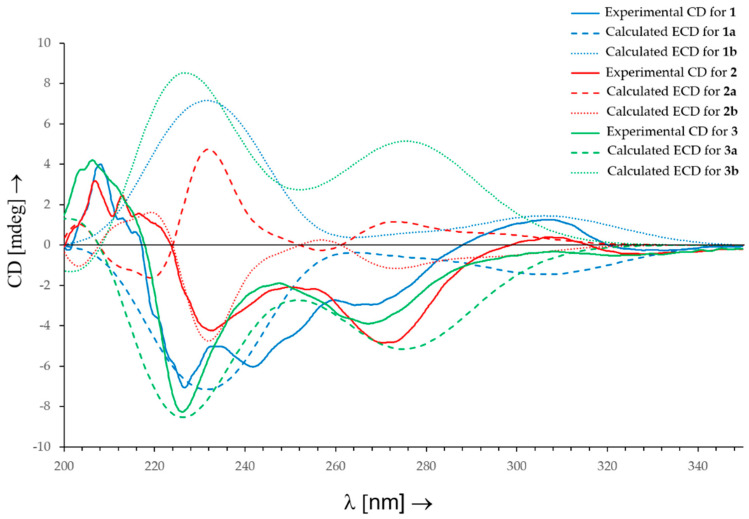
Experimental CD spectra for stachybotrin J (**1**), stachybocin G (**2**), and stachybotrin I (**3**) and Boltzmann-averaged TD-DFT calculated ECD spectra for **1a** (2″*S* 3*R,* 5*S,* 8*R,* 9*R,* 10*S*), **1b** (2″*R*, 3*R,* 5*S,* 8*R,* 9*R,* 10*S*), **2a** (2″*S*, 10*R*, 10′*R*, 13*R*, 13′*R*, 14*S*, 14′*S*, 15*S*, 15′*S*, 18*R*, 18′*R*), **2b** (2″*R*, 10*R*, 10′*R*, 13*R*, 13′*R*, 14*S*, 14′*S*, 15*S*, 15′*S*, 18*R*, 18′*R*), **3a** (2″*S*, 3*R,* 5*S,* 8*R,* 9*R,* 10*S*), and **3b** (2″*R*, 3*R,* 5*S,* 8*R,* 9*R,* 10*S*).

The experimental CD spectrum of **1** exhibited two negative Cotton effects (CEs) at *λ*_max_ = 227 nm and *λ*_max_ = 270 nm. The Boltzmann-averaged TD-DFT calculated ECD spectrum for the most stable conformers of the enantiomer **1a** (2″*S*, 3*R,* 5*S,* 8*R,* 9*R,* 10*S*), performed at the B3LYP/6-311+G(d,p) level of theory, also showed two negative CEs, at *λ*_max_ = 230 nm and *λ*_max_ = 308 nm, which reproduced the signs and differences in amplitude of the experimental CEs. Thus, a 2″*S*-configuration was determined for **1** (Figure 3, Tables S11–S14, Figure S67).

**Figure 4:**
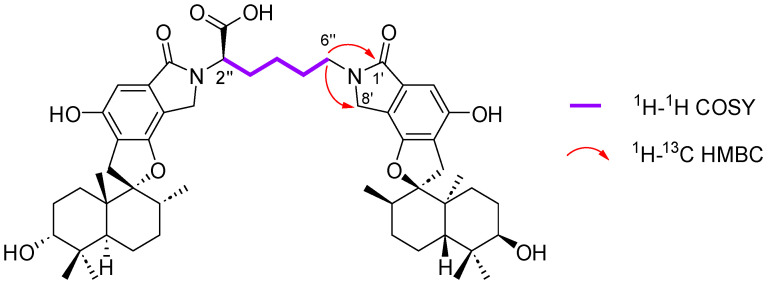
Key 2D NMR correlations for the connecting unit of the dimer stachybocin G (**2**).

The molecular formula of compound **2**, C_52_H_70_N_2_O_10_, was deduced from the HRESI(+)MS analysis which showed a pseudo-molecular ion peak at *m/z* 883.5052 [M + H]^+^ (calcd for C_52_H_71_N_2_O_10_^+^, 883.5103, 19 degrees of unsaturation). The ^13^C NMR spectrum of compound **2** exhibited 23 pairs of almost identical carbon signals characteristic of two spirohydrobenzofuranlactam units. The dimer structure and lysine residue as the connecting unit was confirmed by the proton spin network H-2″–H-3″–H-4″–H-5″–H-6″ revealed by COSY correlations, and H-6″/C-1′ and H-6″/C-8′ key HMBC correlations (Figure 4 and Figure S14).

Comparison of HRMS and NMR data of **2** with previously published data [33], led to its identification as the planar structure of stachybocin A (**33**) (Table S2). The PSD moieties of **2** showed very close chemical shifts by ^1^H and ^13^C NMR spectroscopy to those of stachybotrylactam (**5**) [$\bar{X}$(|Δ*δ*_H(**5**-**2**)_|) = 0.02 ppm and *s* = Δ*δ*_H(**5**-**2**)_ = 0.06 ppm, $\bar{X}$(|Δ*δ*_C(**5**-**2**)_|) = 0.4 ppm and *s* = Δ*δ*_C(**5**-**2**)_ = 1.2 ppm], which allowed us to deduce the following absolute configuration for the spirohydrobenzofuranlactam skeletons: 10*R*, 10′*R*, 14*S*, 14′*S*, 15*S*, 15′*S*, 18*R*, and 18′*R*. A 2″*S*-configuration was determined previously for stachybocin A (**33**) based on a Jones oxidation to yield the phthalimide derivative, followed by a hydrochloric acid hydrolysis to release the amino acid and an HPLC analysis [33]; however, the reported acidic conditions could lead to total or partial racemization of lysine [34]. In this study, a 2″*R-*configuration of compound **2** was deduced by comparison of the experimental CD spectrum (negative CEs at *λ*_max_ = 232 nm and *λ*_max_ = 271 nm) with the Boltzmann-averaged TD-DFT calculated ECD spectrum for the most stable conformers of the enantiomer **2**: **2a** (2″*S*, 10*R*, 10′*R*, 13*R*, 13′*R*, 14*S*, 14′*S*, 15*S*, 15′*S*, 18*R*, 18′*R*), which exhibited positive CEs, at *λ*_max_ = 231 nm and *λ*_max_ = 273 nm, and **2b** (2″*R*, 10*R*, 10′*R*, 13*R*, 13′*R*, 14*S*, 14′*S*, 15*S*, 15′*S*, 18*R*, 18′*R*), which presents negative CEs at *λ*_max_ = 231 nm and *λ*_max_ = 273 nm (Figure 3, Tables S15′S18, Figure S67). Therefore, compound **2** is the C-2″ epimer of stachybocin A (**33**) named stachybocin G (**2**), a new PSD dimer derivative.

The molecular formula of compound **3**, C_32_H_39_NO_6_, was deduced from the HRESI(+)MS analysis which showed a pseudo-molecular ion peak at *m/z* 534.2839 [M + H]^+^ (calcd for C_32_H_40_NO_6_^+^, 534.2850, 14 degrees of unsaturation). Compound **3** showed very close ^1^H and ^13^C NMR chemical shifts for its PSD moiety, which were also very close to those of stachybotrylactam (**5**) [$\bar{X}$(|Δ*δ*_H(**5**-**3**)_|) = 0.04 ppm and *s* = Δ*δ*_H(**5**-**3**)_ = 0.07 ppm], which allowed us to infer the following absolute configuration for **3**: 3*R*, 5*S*, 8*R*, 9*R*, and 10*S*. The ^1^H and ^13^C NMR spectra of **3** featured one methine [*δ*_H_ 5.13 (m, 1H, H-2″)], one methylene [*δ*_H_ 3.54 (m, 1H, H-3a″), 3.21 (m, 1H, H-3b″) and *δ*_C_ 38.0 (C-3″)], one aromatic ring [*δ*_H_ 7.26 (d, ^3^*J* = 7.3 Hz, 2H, H-5″), 7.19 (t, ^3^*J* = 7.3 Hz, 2H, H-6″), 7.10 (t, ^3^*J* = 7.3 Hz, 1H, H-7″) and *δ*_C_ 140.2 (C-4″), 129.6 (C-5″), 129.4 (C-6″), 127.3 (C-7″), 59.7 (C-2″)], and a carboxylic acid carbon (*δ*_C_ 170.3, C-1″), characteristic of the phenylalanine residue of stachybotrin I (**3**) (Table S3) [35]. Stachybotrin I (**3**) (or an isomer) was previously isolated from the culture of a *S. atra* ST002348 [35]; however, (i) multiplicities of the protons in the ^1^H NMR spectrum were not reported, (ii) ^13^C chemical shifts were deduced from the HMQC spectrum, and consequently the ^13^C chemical shifts were not determined for quaternary carbons, and (iii) the relative/absolute configurations were not established. The CD spectrum of **3** exhibits two negative Cotton effects at 226 nm and 268 nm. Comparison with the calculated ECD spectra for the most stable conformers of the two possible enantiomers of **3** [**3a** (2″*S* 3*R,* 5*S,* 8*R,* 9*R,* 10*S*) and **3b** (2″*R*, 3*R,* 5*S,* 8*R,* 9*R,* 10*S*)] allowed us to deduce a 2″*S*-configuration for compound **3** (Figure 3, Tables S19–S22, Figure S67).

An extensive examination of the HRMS and NMR data of **4**–**10** and comparison with previously published data [6,16,17,18,21,36,37] (Tables S4–S10), notably those relating to metabolites isolated from the genus *Stachybotrys*, led to their identification as stachybotrin H (**4**), stachybotrylactam (**5**), stachybotrylactam acetate (**6**), 2*α*-acetoxystachybotrylactam acetate (**7**), stachybotramide (**8**), chartarlactam B (**9**), and F1839-J (**10**), respectively.

**Figure 5:**
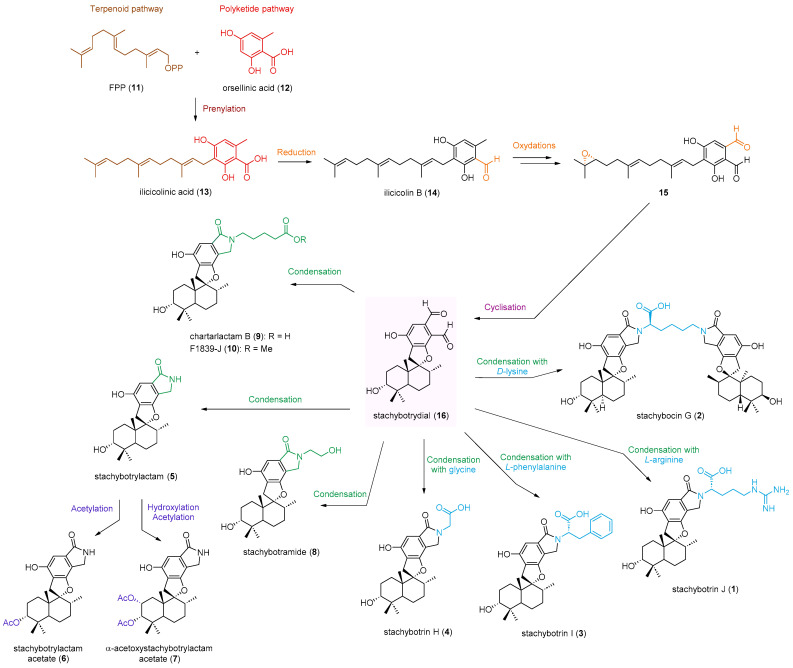
Plausible biogenetic pathway of compounds **1**–**10** isolated from *S. chartarum* MUT 3308.

PSD dimers were scarcely reported in the literature and their origin, natural or artifacts, is still a matter of debate. Recently, Jagels and his group [38] completed the Jarvis hypothesis [36] according to which stachybotrylactam (**5**) and its *N*-functionalized derivatives from *S. chartarum* could be artifacts by showing that isoindolinones production is favored in *N*-rich media. Several plausible biogenetic pathways for PSDs have been proposed. Structurally, PSDs are mainly polyketide–terpenoid hybrid meroterpenoids [39,40]. Compounds **1**–**10** could be derived from a common intermediate, ilicicolin B (**14**), which originates from farnesyldiphosphate (**11**) and orsellinic acid (**12**) (Figure 5). Afterwards, ilicicolin B (**14**) would undergo a series of reactions, notably oxidations and cyclizations, to yield stachybotrydial (**16**) [41]. Stachybotrydial (**16**) could react with a wide range of nucleophiles readily available in the medium, notably amines, for which the complete mechanism is still not fully elucidated [38,42], to give all isoindolinones. For example, ammonia, obtained by the enzymatic conversion of the nitrate present in the medium, would react with stachybotrydial (**16**) to give stachybotrylactam (**5**) [38,43]. In an identical way, amino acids, such as glycine, *L*-phenylalanine, and *L*-arginine, would react with stachybotrydial (**16**) to yield stachybotrin H (**4**), stachybotrin I (**3**), and stachybotrin J (**1**), respectively. Very recently, some PSD derivatives, such as stachybotrin J (**1**), have been obtained by semi-synthesis from stachybotrydial (**16**) and amino acids to support this hypothesis [32]. However, the absolute configurations of these compounds have not been reported. Thus, stachybocin G (**2**) could be obtained by reaction of *D*-lysine with two stachybotrydial (**16**) units or by C-2″ epimerization of stachybocin A (**33**) (Figure 5).

### 2.2. Feature-Based Molecular Networking Analysis

**Figure 6:**
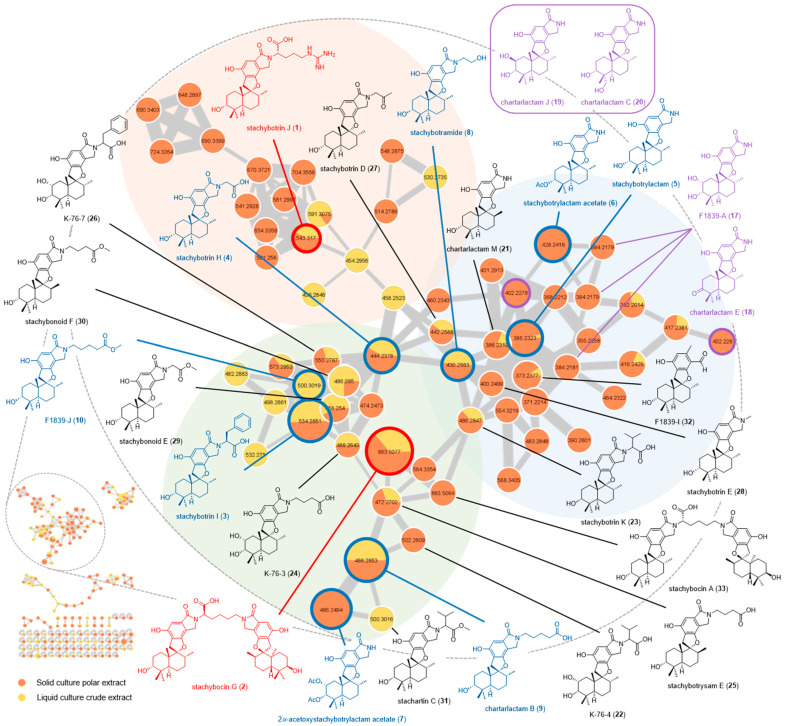
Feature-Based Molecular Network analysis of the crude solid culture extract and the liquid culture filtrate crude extract of *S. chartarum* MUT 3308 (common fragment number: 6; similarity score: 0.6). Nodes are shown as pie charts to reflect the relative abundance of each ion in each of the extracts. Node size represents the total sum of the precursor ion intensity in the MS^1^ scan. Edge thickness corresponds to relative cosine score similarity between nodes. The annotated cluster is enlarged. Isolated molecules are in blue, new molecules are in red, and proposed molecules in black or purple if several nodes could be matched to them.

Mass spectrometry Feature-Based Molecular Networking (FBMN) analyses were performed to putatively assign further PSD derivatives that could be produced by *S. chartarum* MUT 3308 when cultivated in solid (F2: CH_3_OH/CH_2_Cl_2_ (1:1, *v/v*) crude extract) and liquid (AcOEt crude extract) conditions [44]. For this purpose, each fungal crude organic extract was (i) analyzed by UHPLC-HRESIMS(/MS), (ii) preprocessed using MZmine 2 [45], and (iii) analyzed by the FBMN approach to also distinguish possible isomers in the network based on their retention time [44]. The graphical representation of the molecular network (depicting the chemical space present in the MS/MS data) of *S. chartarum* MUT 3308 allowed us to highlight 196 nodes, of which 131 are linked together, which suggests the production of numerous metabolites. The main cluster, dedicated to compounds **1**–**10** and their derivatives, is constituted by 64 nodes, of which 21 nodes (33%) were common to both cultivation conditions, 34 nodes (53%) that were only observed for the solid cultivation condition, and 9 nodes (14%) that were specific to the liquid cultivation condition (Figure 6). The FBMN approach allowed us to assign the isolated compounds **1**–**10** and to annotate seventeen more PSD derivatives. In total, in this cluster, 28 nodes (44%) have been identified (Figure 6, Table S23).

The green subcluster is constituted by 21 nodes, 6 of which were assigned as 2*α*-acetoxystachybotrylactam acetate (**7**) (*m/z* 486.2494, [M + H]^+^; RT = 20.69 min), chartarlactam B (**9**) (*m/z* 486.2853, [M + H]^+^; RT = 20.76 min), F1839-J (**10**) (*m/z* 500.3019, [M + H]^+^; RT = 23.74 min), stachybotrin H (**4**) (*m/z* 444.2378, [M + H]^+^; RT = 20.03 min), stachybotrin I (**3**) (*m/z* 534.2851, [M + H]^+^; RT = 23.43 min), and stachybocin G (**2**) (*m/z* 883.5077, [M + H]^+^; RT = 26.41 min) by comparison with the MS data and the retention time of the isolated compounds **2**, **3**, **4**, **7**, **9**, and **10** (Figure 6, Table S23). Nine nodes were confidently manually annotated to K-76-4 (**22**) (*m/z* 502.2809, [M + H]^+^; RT = 19.33 min), a compound that had never reported named stachybotrin K (**23**) (*m/z* 486.2847, [M + H]^+^; RT = 19.52 min), K-76-3 (**30**) (*m/z* 488.2643, [M + H]^+^; RT = 19.76 min), stachybotrysam E (**25**) (*m/z* 472.2702, [M + H]^+^; RT = 20.33 min), K-76-7 (**26**) (*m/z* 550.2787, [M + H]^+^; RT = 20.86 min), stachybonoid E (**29**) (*m/z* 458.2540, [M + H]^+^; RT = 21.17 min), stachybonoid F (**30**) (*m/z* 486.2850, [M + H]^+^; RT = 22.69 min), stachartin C (**31**) (*m/z* 500.3016, [M + H]^+^; RT = 22.76 min), and stachybocin A (**33**) (*m/z* 883.5064, [M + H]^+^; RT = 24.93 min).

The green subcluster, except for 2*α*-acetoxystachybotrylactam acetate (**7**), is mainly dedicated to *N*-substituted PSD derivatives. K-76-3 (**24**) and stachybotrysam E (**25**) contain a butanoic acid moiety, and chartarlactam B (**9**) features a pentanoic acid moiety [5,16,46]. Stachybonoid F (**30**) and F1839-J (**10**) correspond to the ester derivatives of K-76-3 (**24**) and chartarlactam B (**9**), respectively [6,41]. Stachybotrin H (**4**), stachybotrin I (**3**), stachybotrin K (**23**), and stachartin C (**31**) are amino acid derivatives of stachybotrylactam (**5**) as they feature a glycine, phenylalanine, and valine residue, respectively [35,37,47]. Stachybonoid E (**29**) correspond to the ester derivative of stachybotrin H (**4**) [41]. K-76-7 (**26**) is the hydroxylated derivative of stachybotrin I (**3**) [5].

The blue subcluster comprised 25 nodes, 3 of which were assigned as stachybotrylactam (**5**) (*m/z* 386.2323, [M + H]^+^; RT = 19.99 min), stachybotrylactam acetate (**6**) (*m/z* 428.2418, [M + H]^+^; RT = 22.16 min), and stachybotramide (**8**) (*m/z* 430.2583, [M + H]^+^; RT = 19.57 min), by comparison with the HRMS(/MS) data and the retention time of the isolated compounds **5**, **6**, and **8** (Figure 6, Table S23). Three nodes (*m/z* 384.2179, *m/z* 384.2181; RT = 16.71 min, RT = 18.99 min, RT = 19.72 min) were manually annotated to F1839-A (**17**) ([M-H_2_O+H]^+^), chartarlactam E (**18**) ([M + H]^+^), or their isomers. Two nodes were putatively identified as chartarlactam J (**19**) (*m/z* 402.2278, [M + H]^+^; RT = 17.44 min) or chartarlactam C (**20**) (*m/z* 402.2280, [M + H]^+^; RT = 18.95 min). Four nodes were confidently assigned to chartarlactam M (**21**) (*m/z* 386.2319, [M + H]^+^; RT = 18.86 min), stachybotrin D (**27**) (*m/z* 442.2588, [M + H]^+^; RT = 20.93 min), stachybotrin E (**28**) (*m/z* 400.2499, [M + H]^+^; RT = 20.94 min), and F1839-I (**32**) (*m/z* 373.2377, [M + H]^+^; RT = 23.04 min).

The blue subcluster, except for stachybotramide (**8**), stachybotrin D (**27**), stachybotrin E (**28**), and F1839-I (**32**) [6,21,36,48], is mainly dedicated to PSD derivatives modulated on the drimane skeleton. Chartarlactam J (**19**) and chartarlactam M (**21**) are the C-2 and the C-8 epimers of stachybotrylactam (**5**) and F1839-A (**17**), respectively [16,49]. F1839-A (**17**) and chartarlactam C (**20**) are the C-2 and C-13 hydroxylated analogs of stachybotrylactam (**5**), respectively [16,49]. Chartarlactam E (**18**) is the C-3 oxidized analog of stachybotrylactam (**5**) [16].

The orange subcluster is comprised of 18 nodes and only 1 of which was annotated as stachybotrin J (**1**) (*m/z* 543.3170, [M + H]^+^; RT = 20.61 min) by comparison with the MS data of the isolated compound **1** (Figure 6, Table S23).

All the putatively annotated compounds could also be proposed as their isomers. The relative and/or absolute configuration cannot be determined unless other appropriate spectroscopic techniques are used. Thirty-six nodes with *m/z* values of 355.2258, 368.2212, 371.2214, 382.2014, 390.2601, 416.2429, 417.2381, 431.2913, 436.2846, 454.2956, 458.2523, 460.2343, 474.2473, 482.2883, 483.2848, 484.2322, 498.2861, 501,2580, 514.2786, 530.2735, 532.2710, 541.2928, 548.2875, 554.3219, 568.2212, 573.2953, 581.2997, 584.3354, 591.3075, 648.2897, 654.3358, 670.3721, 690.3399, 690.3403, 704.3556, and 724.3264 could not be assigned to any known metabolite by manual or GNPS-based dereplication approaches.

### 2.3. Biological Assays

Compounds **1**–**10**, isolated in small amounts, were evaluated for their cytotoxicity against five aggressive human cancer cell lines: MP41 (melanoma), 786 (renal carcinoma), 786R (sunitinib-resistant renal cell carcinoma), CAL33 (head and neck carcinoma), and CAL33RR (cisplatin- and radiotherapy-resistant head and neck carcinoma). The above cells were treated with compounds **1**–**10** for two days and XTT assays were used to assess cell metabolism and proliferative capacity. The IC_50_ values (µM) are shown in Table 2. Compounds **5**–**7** showed a weak toxicity against the MP41 cell line (IC_50_ < 1.0 µM) although compounds **1**–**4** and **8**–**10** showed almost no cytotoxicity against the MP41 cell line (IC_50_ > 50 µM). In addition, compounds **5**–**7** exhibited better cytotoxic activities against the 786 and CAL33 cell lines (from 3.6 to 2.5-fold less), with IC_50_ values in the range of 0.3–1.5 µM, compared to sunitinib (IC_50_ = 2.5 ± 0.5) and cisplatin (IC_50_ = 1.5 ± 0.3), respectively, which were used as positive controls. Similarly, compounds **5**–**7** also showed better cytotoxic activities against the two resistant human cancer cell lines, compared to the positive controls, with IC_50_ values from 0.8 to 2.2 µM against 786R (sunitinib IC_50_ > 10 ± 1) and with IC_50_ values ranging from 0.6 to 1.0 µM against CAL33RR (cisplatin IC_50_ > 10 ± 1). Compounds **1**–**4** and **8**–**10** were almost non-cytotoxic against the MP41, 786, 786R, CAL33, and CAL33RR cell lines (except for compound **8**, IC_50_ < 20 µM). Consequently, our results clearly suggest that in terms of structure–activity relationships, a non-substituted lactam functionality in PSDs is required for cytotoxicity against the aggressive human cancer cell lines studied, notably the resistant cancer cell lines. PSDs have hardly been studied for their antitumor activities. In the literature, compounds **5**, **4**, and **8** showed no cytotoxicity against K562 (leukemia), HL60 (leukemia), and Hela (cervical cancer) cell lines (IC_50_ > 100 µM) [37], and compounds **5**, **3**, **7**, and **8** showed no cytotoxicity against NIH-3T3 (fibroblast) and HepG2 (liver carcinoma) cell lines (IC_50_ > 50 µM) [18]. On the other hand, alternative therapies to metastatic RCC and HNSCC are urgently needed to prevent relapse on current conventional treatments (sunitinib for RCC and cisplatin for HNSCC). Recently, *N*,*N′*-diarylureas and thioureas with a nitro-benzothiazole moiety, were synthesized and evaluated by our team for their anticancer properties, particularly against the 786 and CAL33 cell lines. Compared to compounds **5**–**7**, the lead compound of this previous study, named C29, exhibited lower cytotoxic activity against cell lines 786 and CAL33 (1.3- to 6.7-fold higher), with IC_50_ values of 2 and 4 µM, respectively [50]. Taken together, all these data seem to indicate that small modifications of the PSD skeleton could lead to a significant change in bioactivity and/or selectivity against human cancer cell lines, which is of great interest for the development of new anticancer drugs, especially against resistant cancer cell lines.

## 3. Materials and Methods

### 3.1. General

All organic solvents used for material extraction were of analytical grade and purchased from Sigma-Aldrich (Merck KGaA, Saint-Louis, MO, USA). Formic acid (H_2_CO_2_) and acetonitrile used for HPLC were of HPLC grade and both were purchased from CARLO ERBA Reagents GmbH (Emmendingen, Germany). Polygoprep C18 (60–80 µm) for the SPE was purchased from Macherey-Nagel GmbH & Co. KG (Düren, Germany). HPLC analyses and purifications by semi-preparative HPLC were performed with a Waters Alliance 2695 HPLC system (Waters Corporation, Milford, MA, USA) coupled with a Waters 996 photodiode array (PDA) detector and a Shimadzu ELSD LT (Shimadzu, Kyoto, Japan). Analyses were performed with a bifunctional Macherey-Nagel NUCLEODUR Sphynx RP column (250 × 4.6 mm, 5 µm) consisting of a balanced ratio of propylphenyl and C18 ligands. Purifications were performed with a bifunctional Macherey-Nagel NUCLEODUR Sphynx RP (250 × 10 mm, 5 µm) and a Phenomenex Prodigy C18 (250 × 21.2 mm, 5 μm) columns. NMR spectra were recorded with 400 and 500 MHz Bruker Avance NMR spectrometers (Bruker Corporation, Billerica, MA, USA). High resolution mass spectra (HRMS) were conducted on a Thermo Q-Exactive (UPLC-HRMS) Orbitrap (Thermo Fisher Scientific, Waltham, MA, USA) using a ThermoFisher Scientific Hypersil GOLD (150 × 2.1 mm, 1.9 μm) column and a mobile phase A H_2_O + 0.1% formic acid (UPLC/MS grade) and B ACN + 0.1% formic acid (UPLC/MS grade), pumped at a rate of 0.2 mL/min with the following gradient: 0–5 min, 10% B; 5–30 min, 10 to 98% B; 30–35 min, 98% B, and a column reconditioning phase to 10% B for 10 min. The MS parameters were set as follows: spray voltage at 3.7 kV (positive mode) or 2.7 kV (negative mode), capillary temperature at 320 °C, a sheath gas rate at 60 units N_2_ (ca. 200 mL/min), and an auxiliary gas rate at 15 units N_2_ (ca. 50 mL/min). The *m/z* range for data-dependent acquisition was set between 100 and 1200 amu. The data were analyzed using Thermo Xcalibur software. Circular dichroism spectra were measured on a JASCO-J-810 polarimeter (JASCO Corporation, Tokyo, Japan). Optical rotations were recorded on an Anton Paar MCP 150 polarimeter (Anton Paar, Graz, Austria).

### 3.2. Fungal Material and Fermentation

*Stachybotrys chartarum* MUT 3308 was isolated from the marine sponge *Aplysina cavernicola* sampled in the Mediterranean Sea at Villefranche-sur-Mer, France (Lat: 43°41′31.48707839999″ N, Lon: 7°19′12.185658623999″ E) [31]. The fungus was isolated by direct plating of the sponge tissues on Corn Meal Agar Seawater (CMAS; corn meal 2 g, agar 15 g, sea salts mix 30 g, gentamicin sulfate 40 mg, piperacillin and tazobactam 11 mg, Sigma-Aldrich (Merck KGaA, Saint-Louis, MO, USA) up to 1 L DI H_2_O) after incubation at 15 °C. The fungus was identified based on its morphological features and by molecular analyses with the amplification of the Internal Transcribed Spacer—ITS. The sequence was deposited in GenBank (accession number MG980591), while the fungus was preserved at the *Mycotheca Universitatis Taurinensis* (MUT—http://www.mut.unito.it (accessed on 29 January 2023)) of the University of Turin, Italy. The cultivation scale-up for the purification of the compounds was performed on Potato Dextrose Agar (PDA; potato extract 4 g, dextrose 20 g, agar 15 g, up to 1 L DI H_2_O) using 100 Petri dishes (6 cm Ø) or 12 Erlenmeyer flaks (1 L). The plates were incubated for 30 days and the flasks (liquid medium) were agitated for 45 days in the dark at 24 °C.

### 3.3. Extraction and Isolation

The fungal culture on Petri dishes was freeze-dried before extraction. The material was extracted first with CH_2_Cl_2_/AcOEt (1:1, *v*/*v*) and then with CH_2_Cl_2_/CH_3_OH (1:1, *v*/*v*) to yield fractions F1 and F2, respectively. Fraction F2 was fractionated by liquid chromatography on C18 silica gel with a gradient of decreasing polarity (from H_2_O to CH_3_OH to CH_2_Cl_2_) to afford ten fractions according to their chromatographic profile. The CH_3_OH fraction from F2 was further purified by reverse-phase HPLC using a Macherey-Nagel propylphenyl-C18 (250 × 4.6 mm, 5 μm) column to yield pure compounds **5** (6.2 mg, 0.014% *w*/*w*), **6** (2.4 mg, 0.005% *w*/*w*), and **7** (2.3 mg, 0.005% *w*/*w*) using H_2_O–ACN + 0.1% formic acid for each solvent (gradient: 60:40 to 0:100 in 25 min). Fraction F2 was desalted by liquid chromatography on C18 silica gel by eluting first with water then the organic compounds were desorbed by using a mixture of CH_3_OH/CH_2_Cl_2_ (1:1, *v*/*v*) to give two fractions. The organic fraction was then fractionated by liquid chromatography on C18 silica gel with a gradient of decreasing polarity (H_2_O, ACN, CH_3_OH, CH_2_Cl_2_) to give eleven fractions. The H_2_O/ACN (1:1, *v*/*v*) fraction was subjected to HPLC purification (Macherey-Nagel propylphenyl-C18, 250 × 4.6 mm, 5 μm) using H_2_O–ACN + 0.1% formic acid for each solvent (gradient: 90:10 to 0:100 in 25 min) to lead to pure compound **1** (2.1 mg, 0.005% *w*/*w*). The main compounds present in the CH_3_OH fraction were purified by semi-preparative HPLC using H_2_O–ACN + 0.1% formic acid for each solvent (gradient: 70:30 to 0:100 in 25 min) to obtain compounds **2** (2.5 mg, 0.006% *w*/*w*) and **5** (2 mg, 0.004% *w*/*w*).

Two batches of *S. chartarum* MUT 3308 liquid culture (5 L and 7 L) were vacuum filtered to separate the filtrate from the mycelium. The filtrates were subjected to a liquid–liquid extraction with AcOEt to obtain two organic crude extracts. These were fractionated by liquid chromatography on silica diol with a gradient of increasing polarity (Cyclohexane, AcOEt, CH_3_OH) to obtain nineteen fractions. Some fractions, due to their chromatographic similarities following HPLC-PDA-ELSD analyses, were grouped together. Fraction 8 was subjected to purification by semi-preparative HPLC (Gemini C18, 250 × 10 mm, 5 μm) using H_2_O–ACN + 0.1% formic acid for each solvent (gradient: 90:10 to 10:90 in 25 min) to afford compounds **3** (1.7 mg, 0.004%, *w*/*w*) and **10** (1.3 mg, 0.003% *w*/*w*). Fraction 9 was subjected to purification by semi-preparative HPLC (Gemini C18, 250 × 10 mm; 5 μm) using H_2_O–ACN + 0.1% formic acid for each solvent (gradient: 90:10 to 10:90 in 25 min) to afford compounds **3** (1.4 mg, 0.004% *w*/*w*) and **4** (1.2 mg, 0.003% *w*/*w*). Fraction 10 was subjected to purification by semi-preparative HPLC (Gemini C18, 250 × 10 mm; 5 μm) using H_2_O–ACN + 0.1% formic acid for each solvent (gradient: 90:10 to 0:100 in 25 min) to obtain compounds **2** (1 mg, 0.002% *w*/*w*), **5** (1.2 mg, 0.003% *w*/*w*), **8** (0.6 mg, 0.001% *w*/*w*), and **9** (2.1 mg, 0.005% *w*/*w*).

Stachybotrin J (**1**): White amorphous solid; [α]^24^_D_ = −29 (c 0.1, CH_3_OH); UV (CH_3_OH) λ_max_ (log ε): 232 (2.70), 266 (2.80), 303 (0.70) nm; RT = 16.30 min; HRESI(+)MS *m/z* 543.3171 [M + H]^+^ (calcd for C_29_H_43_N_4_O_6_^+^, 543.3177); ^1^H NMR (500 MHz, CD_3_OD), *δ*ppm (mult., *J*): 6.69 (s, 1H, H-3′), 4.76 (m, 1H, H-2″), 4,74 (d, ^3^*J* = 17.2 Hz, 1H, H-8′a), 4.29 (d, ^3^*J* = 17.2 Hz, 1H, H-8′b), 3.33 (s, 1H, H-3), 3.24 (m, 2H, H-5″), 3.24 (d, ^3^*J* = 16.9 Hz, 1H, H-11*α*), 2.86 (d, ^3^*J* = 16.9 Hz, 1H, H-11*β*), 2.15 (m, 2H, H-5, H-3″a), 1.90 (m, 4H, H-1*β*, H-6*α*, H-8, H-3″b), 1.56 (m, 7H, H-2, H-6*β*, H-7, H-4″), 1.09 (m, 1H, H-1*α*), 1.06 (s, 3H, H-15), 0.98 (s, 3H, H-13), 0.89 (s, 3H, H-14), 0.74 (d, ^3^*J* = 6.5 Hz, 3H, H-12). ^13^C NMR (125 MHz, CD_3_OD), *δ*ppm: 171.7 (C-7′), 170.3 (C-1″), 158.6 (C-6″), 157.6 (C-6′), 155.1 (C-2′), 135.1 (C-5′), 118.7 (C-1′), 114.9 (C-4′), 102.1 (C-3′), 99.7 (C-9), 76.3 (C-3), 57.6 (C-2″), 45.9 (C-8′), 43.5 (C-10), 41.9 (C-5″), 41.3 (C-5), 38.6 (C-8), 38.5 (C-4), 33.0 (C-11), 32.3 (C-7), 29.0 (C-13), 28.8 (C-3″), 27.2 (C-4″), 26.4 (C-6), 25.3 (C-1), 23.0 (C-14), 22.1 (C-2), 16.6 (C-15), 16.0 (C-12).

Stachybocin G (**2**): White amorphous solid; [α]^20^_D_ = −35 (c 0.26, CH_3_OH); UV (CH_3_OH) λ_max_ (log ε): 233 (2.65), 266 (2.40), 301 (0.85) nm; RT = 27.26 min; HRESI(+)MS *m/z* 883.5052 [M + H]^+^ (calcd for C_52_H_71_N_2_O_10_^+^, 883.5103); ^1^H NMR (500 MHz, CD_3_OD), *δ*ppm (mult., *J*): 6.67 (s, 1H, H-3), 6.63 (s, 1H, H-3′), 4.76 (m, 1H, H-2″), 4.74 (d, ^3^*J* = 16.9 Hz, 1H, H-8a), 4.48 (d, ^3^*J* = 17.3 Hz, 1H, H-8′a), 4.31 (d, ^3^*J* = 17.3 Hz, 1H, H-8′b), 4.27 (d, ^3^*J* = 16.9 Hz, 1H, H-8b), 3.58 (t, ^3^*J* = 8.0 Hz, 2H, H-6″), 3.33 (m, 2H, H-18, H-18′), 3.21 (d, ^3^*J* = 16.9 Hz, 2H, H-9*α*, H-9′*α*), 2.84 (d, ^3^*J* = 16.9 Hz, 2H, H-9*β*, H-9′*β*), 2.19 (m, 1H, H-3″a), 2.13 (m, 2H, H-14, H-14′), 1.96 (m, 3H, H-13a, H-3″b, H-13′a), 1.84 (m, 5H, H-11, H-16*β*, H-5″, H-11′, H-16′*β*), 1.55 (m, 10H, H-13b, H-12, H-17, H-13′b, H-12′, H-17′), 1.37 (m, 2H, H-4″), 1.08 (m, 2H, H-16*α*, H-16′*α*), 1.05 (s, 6H, H-21, H-21′), 0.98 (s, 6H, H-22, H-22′), 0.88 (s, 6H, H-23, H-23′), 0,72 (d, ^3^*J* = 6.4 Hz, 6H, H-20, H-20′). ^13^C NMR (125 MHz, CD_3_OD), *δ*ppm: 170.3 (C-1, C-1′), 166.3 (C-1″), 157.6 (C-6), 157.5 (C-6′), 155.2 (C-4), 155.0 (C-4′), 135.1 (C-2′), 134.9 (C-2), 118.7 (C-5), 118.6 (C-5′), 114.9 (C-7), 114.2 (C-7′), 102.1 (C-3, C-3′), 99.7 (C-10), 99.6 (C-10′), 76.4 (C-18, C-18′), 57.7 (C-2″), 48.8 (C-8′), 45.9 (C-8), 43.5 (C-15, C-15′, C-6″), 41.3 (C-14, C-14′), 38.7 (C-11), 38.6 (C-11′), 38.5 (C-19), 38.4 (C-19′), 33.0 (C-9, C-9′), 32.3 (C-12, C-12′), 32.2 (C-3″), 29.0 (C-22, C-22′, C-5″), 26.1 (C-16), 26.0 (C-16′), 25.5 (C-13), 25.4 (C-13′), 25.4 (C-4″), 23.0 (C-23, C-23′), 22.1 (C-17, C-17′), 16.6 (C-21), 16.5 (C-21′), 16.0 (C-20, C-20′).

Stachybotrin I (**3**): White amorphous solid; [α]^20^_D_ = −27 (c 0.1, CH_3_OH); UV (CH_3_OH) λ_max_ (log ε): 229 (2.30), 267 (1.10), 303 (0.35) nm; RT = 24.49 min; HRESI(+)MS *m/z* 534.2839 [M + H]^+^ (calcd for C_32_H_40_NO_6_^+^, 534.2850); ^1^H NMR (500 MHz, CD_3_OD), *δ*ppm (mult., *J*): 7.26 (d, ^3^*J* = 7.3 Hz, 2H, H-5″), 7.19 (t, ^3^*J* = 7.3 Hz, 2H, H-6″), 7.10 (t, ^3^*J* = 7.3 Hz, 1H, H-7″), 6.57 (s, 1H, H-3′), 5.13 (m, 1H, H-2″), 4.67 (d, ^3^*J* = 17.0 Hz, 1H, H-8′a), 4.22 (d, ^3^*J* = 17.0 Hz, 1H, H-8′b), 3.54 (m, 1H, H-3″a), 3.35 (s, 1H, H-3), 3.21 (m, 1H, H-3″b), 3.17 (d, ^3^*J* = 16.8 Hz, 1H, H-11*α*), 2.80 (d, ^3^*J* = 16.8 Hz, 1H, H-11*β*), 2.13 (m, 1H, H-5), 1.96 (m, 1H, H-6*α*), 1.84 (m, 2H, H-1*β*, H-8), 1.55 (m, 5H, H-2, H-6*β*, H-7), 1.09 (m, 1H, H-1*α*), 1.04 (s, 3H, H-15), 0.98 (s, 3H, H-13), 0.88 (s, 3H, H-14), 0.67 (d, ^3^*J* = 6.5 Hz, 3H, H-12). ^13^C NMR (125 MHz, CD_3_OD), δppm: 174.5 (C-7′), 170.3 (C-1″), 157.4 (C-6′), 154.8 (C-2′), 140.1 (C-4″), 135.3 (C-5′), 129.6 (C-5″), 129.4 (C-6″), 127.3 (C-7″), 118.5 (C-1′), 114.9 (C-4′), 102.0 (C-3′), 99.5 (C-9), 76.4 (C-3), 59.7 (C-2″), 46.3 (C-8′), 43.5 (C-10), 41.3 (C-5), 38.6 (C-4), 38.5 (C-8), 38.0 (C-3″), 33.0 (C-11), 32.3 (C-7), 29.0 (C-13), 26.1 (C-6), 25.4 (C-1), 23.0 (C-14), 22.1 (C-2), 16.6 (C-15), 16.0 (C-12).

Stachybotrin H (**4**): White amorphous solid; [α]^20^_D_ = −31 (c 0.1, CH_3_OH); UV (CH_3_OH) λ_max_ (log ε): 224 (1.70), 265 (0.55), 302 (0.20) nm; RT = 21.09 min; HRESI(+)MS *m/z* 444.2372 [M + H]^+^ (calcd for C_25_H_34_NO_6_^+^, 444.2381); ^1^H NMR (500 MHz, CD_3_OD), *δ*ppm (mult., *J*): 6.68 (s, 1H, H-3′), 4.48 (m, 2H, H-2″), 4.23 (d, ^3^*J* = 17.0 Hz, 1H, H-8′a), 4.07 (d, ^3^*J* = 17.0 Hz, 1H, H-8′b), 3.33 (s, 1H, H-3), 3.22 (d, ^3^*J* = 16.8 Hz, 1H, H-11*α*), 2.84 (d, ^3^*J* = 16.8 Hz, 1H, H-11*β*), 2.14 (m, 1H, H-5), 1.96 (m, 1H, H-6*α*), 1.86 (m, 2H, H-1*β*, H-8), 1.64 (m, 2H, H-2), 1.58 (m, 3H, H-6*β*, H-7), 1.09 (m, 1H, H-1*α*), 1.05 (s, 3H, H-15), 0.98 (s, 3H, H-13), 0.88 (s, 3H, H-14), 0.73 (d, ^3^*J* = 6.5 Hz, 3H, H-12). ^13^C NMR (125 MHz, CD_3_OD), *δ*ppm: 174.7 (C-7′), 170.4 (C-1″), 157.5 (C-6′), 155.1 (C-2′), 135.2 (C-5′), 118.6 (C-1′), 114.8 (C-4′), 102.2 (C-3′), 99.6 (C-9), 76.5 (C-3), 49.3 (C-2″), 47.4 (C-8′), 43.5 (C-10), 41.3 (C-5), 38.6 (C-4), 38.4 (C-8), 33.0 (C-11), 32.3 (C-7), 28.9 (C-13), 26.1 (C-6), 25.4 (C-1), 23.0 (C-14), 22.1 (C-2), 16.6 (C-15), 15.9 (C-12).

Stachybotrylactam (**5**): White amorphous solid; [α]^20^_D_ = −18 (c 0.24, CH_3_OH) [[α]25D = −19.7 (c 0.05, CH3OH) [16]]; UV (CH_3_OH) λ_max_ (log ε): 230 (3.10), 264 (2.10), 302 (1.00) nm; RT = 21.15 min; HRESI(+)MS *m/z* 386.2314 [M + H]^+^ (calcd for C_23_H_32_NO_4_^+^, 386.2326); ^1^H NMR (500 MHz, CD_3_OD), *δ*ppm (mult., *J*): 6.69 (s, 1H, H-3′), 4.43 (d, ^3^*J* = 17.4 Hz, 1H, H-8′a), 4.27 (d, ^3^*J* = 17.4 Hz, 1H, H-8′b), 3.34 (s, 1H, H-3), 3.24 (d, ^3^*J* = 16.9 Hz, 1H, H-11*α*), 2.86 (d, ^3^*J* = 16.9 Hz, 1H, H-11*β*), 2.13 (m, 1H, H-5), 1.97 (m, 1H, H-6*α*), 1.86 (m, 1H, H-8), 1.84 (m, 1H, H-1*β*), 1.54 (m, 5H, H-2, H-6*β*, H-7), 1.05 (m, 1H, H-1*α*), 1.06 (s, 3H, H-15), 0.99 (s, 3H, H-13), 0.89 (s, 3H, H-14), 0.74 (d, ^3^*J* = 6.5 Hz, 3H, H-12). ^13^C NMR (125 MHz, CD_3_OD), *δ*ppm: 174.1 (C-7′), 157.8 (C-6′), 155.2 (C-2′), 134.7 (C-5′), 119.00 (C-1′), 116.7 (C-4′), 102.1 (C-3′), 99.7 (C-9), 76.5 (C-3), 43.9 (C-8′), 43.5 (C-10), 41.*3* (C-5), 38.6 (C-4), 38.4 (C-8), 33.0 (C-11), 32.3 (C-7), 29.0 (C-13), 26.0 (C-6), 25.4 (C-1), 22.9 (C-14), 22.1 (C-2), 16.5 (C-15), 16.0 (C-12).

Stachybotrylactam acetate (**6**): White amorphous solid; [α]^20^_D_ = −32 (c 0.1, CH_3_OH); UV (CH_3_OH) λ_max_ (log ε): 224 (1.90), 262 (0.55), 302 (0.25) nm; RT = 23.54 min; HRESI(+)MS *m/z* 428.2419 [M + H]^+^ (calcd for C_25_H_34_NO_5_^+^, 428.2431); ^1^H NMR (500 MHz, CD_3_OD), *δ*ppm (mult., *J)*: 6.70 (s, 1H, H-3′), 4.60 (s, 1H, H-3), 4.35 (d, ^3^*J* = 17.2 Hz, 1H, H-8′a), 4.23 (d, ^3^*J* = 17.2 Hz, 1H, H-8′b), 3.23 (d, ^3^*J* = 17.0 Hz, 1H, H-11*α*), 2.88 (d, ^3^*J* = 17.0 Hz, 1H, H-11*β*), 2.16 (m, 1H, H-5), 2.03 (s, 3H, H-17), 1.87 (m, 2H, H-6*α*, H-8), 1.71 (m, 1H, H-1*β*), 1.55 (m, 5H, H-2, H-6*β*, H-7), 1.15 (m, 1H, H-1*α*), 1.08 (s, 3H, H-15), 0.97 (s, 3H, H-13), 0.92 (s, 3H, H-14), 0.76 (d, ^3^*J* = 6.5 Hz, 3H, H-12). ^13^C NMR (125 MHz, CD_3_OD), *δ*ppm: 174.0 (C-7′), 172.4 (C-16), 157.6 (C-6′), 155.4 (C-2′), 134.8 (C-5′), 118.9 (C-1′), 116.2 (C-4′), 102.3 (C-3′), 99.6 (C-9), 79.5 (C-3), 43.9 (C-8′), 43.5 (C-10), 42.3 (C-5), 38.2 (C-8), 37.8 (C-4), 32.9 (C-11), 32.2 (C-7), 28.4 (C-13), 25.9 (C-1), 23.4 (C-6), 22.3 (C-14), 21.9 (C-2), 21.1 (C-17), 16.4 (C-15), 15.9 (C-12).

2α-acetoxystachybotrylactam acetate (**7**): White amorphous solid; [α]^20^_D_ = −30 (c 0.1, CH_3_OH) [[α]25D = −29 (c 0.1, CH3OH) [36]]; UV (CH_3_OH) λ_max_ (log ε): 233 (2.80), 261 (2.60), 302 (1.25) nm; RT = 22.33 min; HRESI(+)MS *m/z* 486.2474 [M + H]^+^ (calcd for C_27_H_36_NO_7_^+^, 486.2486); ^1^H NMR (500 MHz, CD_3_OD), *δ*ppm (mult., *J*): 6.73 (s, 1H, H-3′), 5.23 (m, 1H, H-2), 4.95 (s, 1H, H-3), 4.36 (d, ^3^*J* = 17.2 Hz, 1H, H-8′a), 4.24 (d, ^3^*J* = 17.2 Hz, 1H, H-8′b), 3.24 (d, ^3^*J* = 17.1 Hz, 1H, H-11*α*), 2.93 (d, ^3^*J* = 17.1 Hz, 1H, H-11*β*), 2.13 (m, 1H, H-5), 2.07 (s, 3H, H-17), 1.91 (m, 1H, H-8), 1.86 (s, 3H, H-19), 1.82 (m, 1H, H-1*β*), 1.66-1.54 (m, 4H, H-6, H-7), 1.40 (m, 1H, H-1*α*), 1.16 (s, 3H, H-15), 0.94 (s, 3H, H-14), 1.05 (s, 3H, H-13), 0.78 (d, ^3^*J* = 6.5 Hz, 3H, H-12). ^13^C NMR (125 MHz, CD_3_OD), *δ*ppm: 173.8 (C-7′), 172.4 (C-18), 172.3 (C-16), 157.4 (C-6′), 155.5 (C-2′), 135.1 (C-5′), 118.6 (C-1′), 116.9 (C-4′), 102.5 (C-3′), 99.2 (C-9), 78.3 (C-3), 69.6 (C-2), 44.8 (C-10), 43.8 (C-8′), 41.9 (C-5), 39.1 (C-4), 37.8 (C-8), 33.0 (C-11), 32.0 (C-7), 31.5 (C-1), 22.0 (C-14), 28.3 (C-13), 21.5 (C-6), 20.9 (C-17), 20.8 (C-19), 17.3 (C-15), 15.8 (C-12).

Stachybotramide (**8**): White amorphous solid; [α]^20^_D_ = −19 (c 0.04, CH_3_OH) [[α]25D = −16 (c 0.1, CH3OH) [36]]; UV (CH_3_OH) λ_max_ (log ε): 233 (2.00), 265 (0.70), 300 (0.25) nm; RT = 20.21 min; HRESI(+)MS *m/z* 430.2581 [M + H]^+^ (calcd for C_25_H_36_NO_5_^+^, 430.2588); ^1^H NMR (500 MHz, CD_3_OD), *δ*ppm (mult., *J*): 6.67 (s, 1H, H-3′), 4.57 (d, ^3^*J* = 17.2 Hz, 1H, H-8′a), 4.43 (d, ^3^*J* = 17.2 Hz, 1H, H-8′b), 3.80 (t, ^3^*J* = 5.4 Hz, 2H, H-2″), 3.70 (t, ^3^*J* = 5.4 Hz, 2H, H-1″), 3.35 (s, 1H, H-3), 3.23 (d, ^3^*J* = 16.9 Hz, 1H, H-11*α*), 2.85 (d, ^3^*J* = 16.9 Hz, 1H, H-11*β*), 2.14 (m, 1H, H-5), 1.97 (m, 1H, H-6*α*), 1.86 (m, 2H, H-1*β*, H-8), 1.57 (m, 5H, H-2, H-6*β*, H-7), 1.10 (m, 1H, H-1*α*), 1.06 (s, 3H, H-15), 0.98 (s, 3H, H-13), 0.89 (s, 3H, H-14), 0.73 (d, ^3^*J* = 6.5 Hz, 3H, H-12).

Chartarlactam B (**9**): White amorphous solid; [α]^20^_D_ = −24 (c 0.1, CH_3_OH) [[α]25D = −22 (c 0.05, CH3OH) [16]]; UV (CH_3_OH) λ_max_ (log ε): 223.8 (2.05), 265 (0.75), 300 (0.25) nm; RT = 21.63 min; HRESI(+)MS *m/z* 486.2838 [M + H]^+^ (calcd for C_28_H_40_NO_6_^+^, 486.2850); ^1^H NMR (500 MHz, CD_3_OD), *δ*ppm (mult., *J*): 6.65 (s, 1H, H-3′), 4.47 (d, ^3^*J* = 17.2 Hz, 1H, H-8′a), 4.33 (d, ^3^*J* = 17.2 Hz, 1H, H-8′b), 3.61 (t, ^3^*J* = 7.0 Hz, 2H, H-1″), 3.33 (s, 1H, H-3), 3.22 (d, ^3^*J* = 16.9 Hz, 1H, H-11*α*), 2.85 (d, ^3^*J* = 16.9 Hz, 1H, H-11*β*), 2.23 (t, ^3^*J* = 7.3 Hz, 2H, H-4″), 2.13 (m, 1H, H-5), 1.96 (m, 1H, H-6*α*), 1.84 (m, 2H, H-1*β*, H-8), 1.73 (m, 2H, H-2″), 1.67 (m, 2H, H-3″), 1.59 (m, 4H, H-2, H-7), 1.51 (m, 1H, H-6*β*), 1.08 (m, 1H, H-1*α*), 1.05 (s, 3H, H-15), 0.98 (s, 3H, H-13), 0.89 (s, 3H, H-14), 0.74 (d, ^3^*J* = 6.5 Hz, 3H, H-12). ^13^C NMR (125 MHz, CD_3_OD), *δ*ppm: 171.7 (C-7′), 170.3 (C-5″), 157.6 (C-6′), 155.2 (C-2′), 135.2 (C-5′), 118.6 (C-1′), 114.3 (C-4′), 102.0 (C-3′), 99.7 (C-9), 76.4 (C-3), 43.6 (C-8′), 43.5 (C-10, C-1″), 41.3 (C-5), 38.6 (C-4″), 38.5 (C-4), 38.4 (C-8), 32.9 (C-11), 32.3 (C-7), 29.5 (C-2″), 29.0 (C-13), 26.0 (C-6), 25.4 (C-1), 25.0 (C-3″), 23.0 (C-14), 22.1 (C-2), 16.5 (C-15), 16.0 (C-12).

F1839-J (**10**): White amorphous solid; [α]^20^_D_ = −12 (c 0.2, CH_3_OH); UV (CH_3_OH) λ_max_ (log ε): 224 (2.25), 265 (0.80), 301 (0.25) nm; RT = 24.77 min; HRESI(+)MS *m/z* 500.2995 [M + H]^+^ (calcd for C_29_H_42_NO_6_^+^, 500.3007); ^1^H NMR (500 MHz, CD_3_OD), *δ*ppm (mult., *J*): 6.67 (s, 1H, H-3′), 4.69 (d, ^3^*J* = 16.9 Hz, 1H, H-8′a), 4.24 (d, ^3^*J* = 16.9 Hz, 1H, H-8′b), 3.87 (m, 1H, H-1″a), 3.78 (m, 1H, H-1″b), 3.65 (m, 3H, H-6″), 3.35 (s, 1H, H-3), 3.22 (d, ^3^*J* = 16.9 Hz, 1H, H-11*α*), 2.85 (d, ^3^*J* = 16.9 Hz, 1H, H-11*β*), 2.26 (m, 2H, H-4″), 2.11 (m, 1H, H-5), 1.93 (m, 1H, H-6*α*), 1.88 (m, 1H, H-1*β*), 1.83 (m, 3H, H-8, H-2″), 1.60 (m, 4H, H-7, H-3″), 1.54 (m, 3H, H-2, H-6*β*), 1.10 (m, 1H, H-1*α*), 1.06 (s, 3H, H-15), 0.99 (s, 3H, H-13), 0.89 (s, 3H, H-14), 0.73 (d, ^3^*J* = 6.5 Hz, 3H, H-12).

### 3.4. Feature-Based Molecular Networking Analysis

The data were processed by using the FBMN method [44]. The data files were converted from the raw data format to mzXML format using MSConvert software (ProteoWizard package 3.0). All mzxml values were processed using MZmine 2.53 [45]. Mass detection was realized with an MS^1^ noise level of 5 × 10^6^ and an MS^2^ noise level of 5 × 10^3^. The ADAP chromatogram builder was employed with a minimum group size of scans of 5, a group intensity threshold of 5 × 10^6^, a minimum highest intensity of 1.7 × 10^7^, and *m/z* tolerance of 0.0 (or 10 ppm). Deconvolution was performed with the Baseline cut-off algorithm according to the following settings: minimum peak height of 2.7 × 10^7^, peak duration range of 0.1–2 min, baseline level of 3 × 10^6^, and an auto *m/z* center calculation. MS/MS scans were paired using a *m/z* tolerance range of 0.02 Da and RT tolerance range of 0.1 min. Isotopologs were grouped using the isotopic peak grouper algorithm with a *m/z* tolerance of 0.0 (or 10 ppm) and a RT tolerance of 0.2 min. Peaks were filtered using a feature list row filter, keeping only peaks with MS/MS scans (GNPS). Peak alignment was performed using the join aligner with a *m/z* tolerance of 0.0 (or 10 ppm), a weight for *m/z* at 75%, a RT tolerance of 0.2 min and weight for RT at 25%. The MGF file and the metadata were generated using the export/submit to GNPS option [51]. The molecular network was calculated and visualized using Cytoscape software [52]. The parent mass tolerance was 0.02 Da and the MS/MS fragment ion tolerance was 0.02 Da. The edges were filtered to have a cosine score above 0.6 and more than 6 matched peaks.

### 3.5. Computational Analysis

TD-DFT calculations of ECD spectra were performed with the Gaussian 16 program package [53]. Conformer distribution analysis and geometry optimizations for all structures were carried out using the AM1 semi-empirical force field implemented in the Spartan 08 program. For each compound, the minimum energy structures were filtered and checked for duplicity. Then, each conformer was geometrically optimized using the hybrid DFT method B3LYP and the basis set 6-31+G(d,p) (B3LYP/6-31+G(d,p)), with thermochemical parameters and the frequencies at 298 K and 1 atm. The solvation effects of methanol were modelized with the polarizable continuum model (PCM). From the TD-DFT calculations performed on each structure optimized conformer, the calculated excitation energy (in nm) and rotatory strength *R*, in dipole velocity (*Rvel*) and dipole length (*Rlen*) forms, were simulated into an ECD curve by using the following Gaussian function (1):

(1) $$\Delta \varepsilon \left(E\right)=\overset{n}{\underset{i=1}{\sum }}\varepsilon_{i}\left(E\right)=\overset{n}{\underset{i=1}{\sum }}\left(\frac{R_{i}E_{i}}{2.29\;\times \;10^{-39}\sqrt{\pi \sigma }}exp\left[-{\left(\frac{E-E_{i}}{\sigma }\right)}^{2}\right]\right)\;$$

where σ is the width of the band at 1/*e* height, and *E*i and *R*i are the excitation energies and rotatory strengths for transition *i*, respectively. σ = 0.30 eV and *R*vel were used. The Boltzmann-averaged ECD spectra were obtained from B3LYP/6-31+G(d,p)-optimized structures. All DFT and TD-DFT calculations were performed using HPC resources from Azzurra.

### 3.6. Cell Culture

The human head and neck squamous cell carcinoma (HNSCC) cell line CAL33 (DSMZ, ACC 447) was provided through a Material Transfer Agreement with the Oncopharmacology Laboratory, Centre Antoine Lacassagne (CAL), where it had initially been isolated [54]. CAL33RR cells were generated by chronic exposure to cisplatin and several round of irradiation by 8 gray X-rays [55]. The kidney cancer cell line 786-0 (ATCC, CRL-1932) was purchased from the American Tissue Culture Collection. The 786R cell line was generated by chronic exposure to sunitinib [56]. The uveal melanoma cell line MP41 (ATCC, CRL-3297) was purchased from the American Tissue Culture Collection. The cells were cultured in Dulbecco’s Modified Eagle medium (DMEM; Gibco) supplemented with 7% fetal bovine serum (Thermo Fisher Scientific, Waltham, MA, USA).

### 3.7. Cytotoxicity Measurement (XTT)

The cells (5 × 10^3^ cells/100 μL) were incubated in a 96-well plate with different concentrations of the drugs for 48 h. Fifty microliters of XTT reagent were added to each well. Each assay was performed in triplicate. The assay is based on the cleavage of the tetrazolium salt 2,3-Bis-(2-methoxy-4-nitro-5-sulfophenyl)–2*H*-tetrazolium-5-carboxanilide (XTT, Sigma-Aldrich, Merck KGaA, Saint-Louis, MO, USA) in the presence of an electron-coupling reagent to produce a soluble formazan salt. This conversion only occurs in viable (metabolically active) cells. The number of viable cells is directly correlated with the amount of orange formazan by measuring the absorbance of the dye at 450 nm on a spectrophotometer.

## 4. Conclusions

The marine sponge-associated fungus *S. chartarum* MUT 3308*,* grown in solid and liquid media, was studied for its ability to produce PSDs, a family of metabolites with interesting biological properties. Two PSDs that had never been isolated before, stachybotrin J (**1**) and new stachybocin G (**2**), along with eight already reported analogues, stachybotrin I (**3**), stachybotrin H (**4**), stachybotrylactam (**5**), stachybotrylactam acetate (**6**), 2*α*-acetoxystachybotrylactam acetate (**7**), stachybotramide (**8**), chartarlactam B (**9**), and F1839-J (**10**), were isolated and fully characterized. Although previously found in the genus *Stachybotrys*, this is the first time that stachybotrin H (**4**) and F1839-J (**10**) have been isolated from *S. chartarum*. A plausible biosynthetic hypothesis has been proposed for compounds **1**–**10**. FBMN analysis led us to hypothesize the presence of numerous derivatives and seventeen have been putatively identified. Compounds **5**, **6**, and **7** showed cytotoxicity against resistant human cancer cell lines in the range of 0.3–2.2 µM. Our data seem to indicate that small modifications on phenylspirodrimane structures could result in a significant change in cytotoxicity activities, which is of value for the development of new anticancer drugs.

## Figures and Tables

**Table 1:** 1D and 2D NMR data (500/125 MHz, CD_3_OD) for stachybotrin J (**1**).

N°	*δ*_C_ (ppm)/Mult.	*δ*_H_ (ppm)/Mult./*J*(Hz)	^1^H-^1^H COSY	^1^H-^13^C HMBC
1*α*	25.3, CH_2_	1.09, m	1*β*, 2, 15	-
1*β*		1.90, m	1*α*, 2, 15	
2	22.1, CH_2_	1.56, m	1*α*, 1*β*, 3	-
3	76.3, CH	3.33, s	-	-
4	38.5, C	-	-	-
5	41.3, CH	2.15, m	6*α*, 6*β*	13, 14
6*α*	26.1, CH	1.90, m	5, 7	-
6*β*		1.56, m		
7	32.3, CH_2_	1.56, m	6*α*, 6*β*	-
8	38.6, CH	1.90, m	7, 12	-
9	99.7, C	-	-	-
10	43.5, C	-	-	-
11*α*	33.0, CH	3.24, d, *16.9*	11*β*	8, 9, 10, 1′, 6′
11*β*		2.86, d, *16.9*	11*α*	
12	16.0, CH_3_	0.74, d, *6.5*	8	7, 8, 9
13	29.0, CH_3_	0.98, s	-	3, 4, 5, 14
14	23.0, CH_3_	0.89, s	-	3, 4, 5, 13
15	16.6, CH_3_	1.06, s	1*α*, 1*β*	1, 5, 9, 10
1′	118.7, C	-	-	-
2′	155.1, C	-	-	-
3′	102.1, CH	6.69, s	-	1′, 2′, 4′, 7′
4′	114.9, C	-	-	-
5′	135.1, C	-	-	-
6′	157.6, C	-	-	-
7′	171.7, C	-	-	-
8′a	45.9, CH	4.74, d, *17.2*	8′b	4′, 5′, 7′
8′b		4.29, d, *17.2*	8′a	
1″	170.3, C	-	-	-
2″	57.6, CH	4.76, m	3″a, 3″b	7′, 8′, 1″, 3″
3″a	28.8, CH_2_	2.15, m	2″, 4″	4″
3″b		1.90, m		
4″	27.2, CH_2_	1.56, m	3″a, 3″b, 5″	-
5″	41.9, CH_2_	3.24, m	4″	4″, 6″
6″	158.6, C	-	-	-

**Table 2:** Antiproliferative activities (IC_50_ (µM)) of compounds **1**–**10** on human cancer cell lines.

	IC_50_ (μM)				
Molecule	MP41	786	786R	CAL33	CAL33RR
**1**	>100	100	>100	20 ± 4	100 ± 7
**2**	>100	85 ± 7	>100	30 ± 4	>100
**3**	>100	>100	>100	40 ± 4	97 ± 5
**4**	>100	20 ± 4	80 ± 6	40 ± 4	100 ± 7
**5**	<1	0.7 ± 0.1	0.8 ± 0.2	0.3 ± 0.1	0.6 ± 0.2
**6**	0.6 ± 0.1	1.5 ± 0.2	2.2 ± 0.3	0.6 ± 0.1	1.0 ± 0.2
**7**	0.4 ± 0.1	0.8 ± 0.2	2.2 ± 0.4	0.4 ± 0.1	0.7 ± 0.2
**8**	50 ± 5	>100	74 ± 5	5 ± 5	97 ± 5
**9**	>100	60 ± 6	>100	30 ± 3	69 ± 6
**10**	>100	30 ± 6	75 ± 5	35 ± 4	97 ± 6
**cisplatin**	-	-	-	1.5 ± 0.3	>10 ± 1
**sunitinib**	-	2.5 ± 0.5	>10 ± 1	-	-

## Data Availability

Not applicable.

## Supplementary Materials

The following are available online at https://www.mdpi.com/article/10.3390/md21030135/s1, Figure S1: HRESI(+)MS spectrum of stachybotrin J (**1**); Figure S2: ^1^H NMR spectrum (500 MHz, CD_3_OD) of stachybotrin J (**1**); Figure S3: ^13^C NMR spectrum (125 MHz, CD_3_OD) of stachybotrin J (**1**); Figure S4: ^1^H-^1^H COSY NMR spectrum (500/500 MHz, CD_3_OD) of stachybotrin J (**1**); Figure S5: ^1^H-^13^C HSQC NMR spectrum (500/125 MHz, CD_3_OD) of stachybotrin J (**1**); Figure S6: ^1^H-^13^C HMBC NMR spectrum (500/125 MHz, CD_3_OD) of stachybotrin J (**1**); Figure S7: ^1^H-^1^H NOESY NMR spectrum (500/500 MHz, CD_3_OD) of stachybotrin J (**1**); Figure S8: HRESI(+)MS spectrum of stachybocin G (**2**); Figure S9: ^1^H NMR spectrum (500 MHz, CD_3_OD) of stachybocin G (**2**); Figure S10: ^13^C NMR spectrum (125 MHz, CD_3_OD) of stachybocin G (**2**); Figure S11: ^1^H-^1^H COSY NMR spectrum (500/500 MHz, CD_3_OD) of stachybocin G (**2**); Figure S12: ^1^H-^13^C HSQC NMR spectrum (500/125 MHz, CD_3_OD) of stachybocin G (**2**); Figure S13: ^1^H-^13^C HMBC NMR spectrum (500/125 MHz, CD_3_OD) of stachybocin G (**2**); Figure S14: Zoom of the ^1^H-^13^C HMBC NMR spectrum (500/125 MHz, CD_3_OD) of stachybocin G (**2**); Figure S15: ^1^H-^1^H NOESY NMR spectrum (500/500 MHz, CD_3_OD) of stachybocin G (**2**); Figure S16: ^1^H NMR spectrum (500 MHz, (CD_3_)_2_SO) of stachybocin G (**2**); Figure S17: ^13^C NMR spectrum (125 MHz, (CD_3_)_2_SO) of stachybocin G (**2**); Figure S18: ^1^H-^1^H COSY NMR spectrum (500/500 MHz, (CD_3_)_2_SO) of stachybocin G (**2**); Figure S19: ^1^H-^13^C HSQC NMR spectrum (500/125 MHz, (CD_3_)_2_SO) of stachybocin G (**2**); Figure S20: ^1^H-^13^C HMBC NMR spectrum (500/125 MHz, (CD_3_)_2_SO) of stachybocin G (**2**); Figure S21: HRESI(+)MS spectrum of stachybotrin I (**3**); Figure S22: ^1^H NMR spectrum (500 MHz, CD_3_OD) of stachybotrin I (**3**); Figure S23: ^13^C NMR spectrum (125 MHz, CD_3_OD) of stachybotrin I (**3**); Figure S24: ^1^H-^1^H COSY NMR spectrum (500/500 MHz, CD_3_OD) of stachybotrin I (**3**); Figure S25: ^1^H-^13^C HSQC NMR spectrum (500/125 MHz, CD_3_OD) of stachybotrin I (**3**); Figure S26: HRESI(+)MS spectrum of stachybotrin H (**4**); Figure S27: ^1^H NMR spectrum (500 MHz, CD_3_OD) of stachybotrin H (**4**); Figure S28: ^13^C NMR spectrum (125 MHz, CD_3_OD) of stachybotrin H (**4**); Figure S29: ^1^H-^1^H COSY NMR spectrum (500/500 MHz, CD_3_OD) of stachybotrin H (**4**); Figure S30: ^1^H-^13^C HSQC NMR spectrum (500/125 MHz, CD_3_OD) of stachybotrin H (**4**); Figure S31: ^1^H-^13^C HMBC NMR spectrum (500/125 MHz, CD_3_OD) of stachybotrin H (**4**); Figure S32: ^1^H-^1^H NOESY NMR spectrum (500/500 MHz, CD_3_OD) of stachybotrin H (**4**); Figure S33: HRESI(+)MS spectrum of stachybotrylactam (**5**); Figure S34: ^1^H NMR spectrum (500 MHz, CD_3_OD) of stachybotrylactam (**5**); Figure S35: ^13^C NMR spectrum (125 MHz, CD_3_OD) of stachybotrylactam (**5**); Figure S36: ^1^H-^1^H COSY NMR spectrum (500/500 MHz, CD_3_OD) of stachybotrylactam (**5**); Figure S37: ^1^H-^13^C HSQC NMR spectrum (500/125 MHz, CD_3_OD) of stachybotrylactam (**5**); Figure S38: ^1^H-^13^C HMBC NMR spectrum (500/125 MHz, CD_3_OD) of stachybotrylactam (**5**); Figure S39: ^1^H-^1^H NOESY NMR spectrum (500/500 MHz, CD_3_OD) of stachybotrylactam (**5**); Figure S40: HRESI(+)MS spectrum of stachybotrylactam acetate (**6**); Figure S41: ^1^H NMR spectrum (500 MHz, CD_3_OD) of stachybotrylactam acetate (**6**); Figure S42: ^13^C NMR spectrum (125 MHz, CD_3_OD) of stachybotrylactam acetate (**6**); Figure S43: ^1^H-^1^H COSY NMR spectrum (500/500 MHz, CD_3_OD) of stachybotrylactam acetate (**6**); Figure S44: ^1^H-^13^C HSQC NMR spectrum (500/125 MHz, CD_3_OD) of stachybotrylactam acetate (**6**); Figure S45: ^1^H-^13^C HMBC NMR spectrum (500/125 MHz, CD_3_OD) of stachybotrylactam acetate (**6**); Figure S46: ^1^H-^1^H NOESY NMR spectrum (500/500 MHz, CD_3_OD) of stachybotrylactam acetate (**6**); Figure S47: HRESI(+)MS spectrum of 2*α*-acetoxystachybotrylactam acetate (**7**); Figure S48: ^1^H NMR spectrum (500 MHz, CD_3_OD) of 2*α*-acetoxystachybotrylactam acetate (**7**); Figure S49: ^13^C NMR spectrum (125 MHz, CD_3_OD) of 2*α*-acetoxystachybotrylactam acetate (**7**); Figure S50: ^1^H-^1^H COSY NMR spectrum (500/500 MHz, CD_3_OD) of 2*α*-acetoxystachybotrylactam acetate (**7**); Figure S51: ^1^H-^13^C HSQC NMR spectrum (500/125 MHz, CD_3_OD) of 2*α*-acetoxystachybotrylactam acetate (**7**); Figure S52: ^1^H-^13^C HMBC NMR spectrum (500/125 MHz, CD_3_OD) of 2*α*-acetoxystachybotrylactam acetate (**7**); Figure S53: ^1^H-^1^H NOESY NMR spectrum (500/500 MHz, CD_3_OD) of 2*α*-acetoxystachybotrylactam acetate (**7**); Figure S54: HRESI(+)MS spectrum of stachybotramide (**8**); Figure S55: ^1^H NMR spectrum (500 MHz, CD_3_OD) of stachybotramide (**8**); Figure S56: ^1^H-^1^H COSY NMR spectrum (500/500 MHz, CD_3_OD) of stachybotramide (**8**); Figure S57: HRESI(+)MS spectrum of chartarlactam B (**9**); Figure S58: ^1^H NMR spectrum (500 MHz, CD_3_OD) of chartarlactam B (**9**); Figure S59: ^13^C NMR spectrum (125 MHz, CD_3_OD) of chartarlactam B (**9**); Figure S60: ^1^H-^1^H COSY NMR spectrum (500/500 MHz, CD_3_OD) of chartarlactam B (**9**); Figure S61: ^1^H-^13^C HSQC NMR spectrum (500/125 MHz, CD_3_OD) of chartarlactam B (**9**); Figure S62: ^1^H-^13^C HMBC NMR spectrum (500/125 MHz, CD_3_OD) of chartarlactam B (**9**); Figure S63: ^1^H-^1^H NOESY NMR spectrum (500/500 MHz, CD_3_OD) of chartarlactam B (**9**); Figure S64: HRESI(+)MS spectrum of F1839-J (**10**); Figure S65: ^1^H NMR spectrum (500 MHz, CD_3_OD) of F1839-J (**10**); Figure S66: HPLC-PDA-ELSD chromatograms of compounds **1**–**10**; Table S1: ^1^H and ^13^C NMR data of **1** (500/125 MHz, CD_3_OD) and synthetic stachybotry-arginine (600/150 MHz, (CD_3_OD); Table S2: ^1^H and ^13^C NMR data of **2** (500/125 MHz, (CD_3_)_2_SO) and stachybocin A (400/100 MHz, (CD_3_)_2_SO); Table S3: ^1^H and ^13^C NMR data of **3** (500/125 MHz, CD_3_OD) and stachybotrin I (400/100 MHz, (CD_3_)_2_SO); Table S4: ^1^H and ^13^C NMR data of **4** (500/125 MHz, CD_3_OD) and stachybotrin H (400/100 MHz, (CD_3_)_2_SO); Table S5: ^1^H and ^13^C NMR data of **5** (500/125 MHz, CD_3_OD) and stachybotrylactam ((CD_3_)_2_CO, 200 MHz; CDCl_3_, 50 MHz); Table S6: ^1^H and ^13^C NMR data of **6** (500/125 MHz, CD_3_OD) and stachybotrylactam acetate (200/50 MHz, CDCl_3_); Table S7: ^1^H and ^13^C NMR data of **7** (500/125 MHz, CD_3_OD) and 2α-acetoxystachybotrylactam acetate (200/50 MHz, CDCl_3_); Table S8: ^1^H NMR data of **8** (500 MHz, CD_3_OD) and stachybotramide (50 MHz, C_5_D_6_N); Table S9: ^1^H and ^13^C NMR data of **9** (500/125 MHz, CD_3_OD) and chartarlactam B (500/125 MHz, (CD_3_)_2_SO); Table S10: ^1^H NMR data of **10** (500 MHz, CD_3_OD) and F1839-J (270 MHz, C_5_D_6_N); Table S11: Coordinates (Ångstroms) for conformer (2″*S*, 3*R,* 5*S,* 8*R,* 9*R,* 10*S*)-**1**_C1 (Energy: −1800.701682 Hartree, Solvent: CH_3_OH, Boltzmann %: 58.3); Table S12: Coordinates (Ångstroms) for conformer (2″*S*, 3*R,* 5*S,* 8*R,* 9*R,* 10*S*)-**1**_C2 (Energy: −1800.70038 Hartree, Solvent: CH_3_OH, Boltzmann %: 14.6); Table S13: Coordinates (Ångstroms) for conformer (2″*S*, 3*R,* 5*S,* 8*R,* 9*R,* 10*S*)-**1**_C3 (Energy: −1800.700959 Hartree, Solvent: CH_3_OH, Boltzmann %: 27.1); Table S14: Coordinates (Ångstroms) for conformer (2″*S*, 3*R,* 5*S,* 8*R,* 9*R,* 10*S*)-**1**_C4 (Energy: −1800.691933 Hartree, Solvent: CH_3_OH, Boltzmann %: 1.91.10^−2^); Table S15: Coordinates (Ångstroms) for conformer (2″*R*, 10*R*, 10′*R*, 13*R*, 13′*R*, 14*S*, 14′*S*, 15*S*, 15′*S*, 18*R*, 18′*R*)-**2**_C1 (Energy: −2885.29118 Hartree, Solvent: CH_3_OH, Boltzmann %: 44.8); Table S16: Coordinates (Ångstroms) for conformer (2″*R*, 10*R*, 10′*R*, 13*R*, 13′*R*, 14*S*, 14′*S*, 15*S*, 15′*S*, 18*R*, 18′*R*)-**2**_C2 (Energy: −2885.28789 Hartree, Solvent: CH_3_OH, Boltzmann %: 1.4); Table S17: Coordinates (Ångstroms) for conformer (2″*R*, 10*R*, 10′*R*, 13*R*, 13′*R*, 14*S*, 14′*S*, 15*S*, 15′*S*, 18*R*, 18′*R*)-**2**_C3 (Energy: −2885.29129 Hartree, Solvent: CH_3_OH, Boltzmann %: 50.2); Table S18: Coordinates (Ångstroms) for conformer (2″*R*, 10*R*, 10′*R*, 13*R*, 13′*R*, 14*S*, 14′*S*, 15*S*, 15′*S*, 18*R*, 18′*R*)-**2**_C4 (Energy: −2885.28879 Hartree, Solvent: CH_3_OH, Boltzmann %: 3.6); Table S19: Coordinates (Ångstroms) for conformer (2″*S*, 3*R,* 5*S,* 8*R,* 9*R,* 10*S*)-**3**_C1 (Energy: −1748.9471 Hartree, Solvent: CH_3_OH, Boltzmann %: 73.9); Table S20: Coordinates (Ångstroms) for conformer (2″*S*, 3*R,* 5*S,* 8*R,* 9*R,* 10*S*)-**3**_C2 (Energy: −1748.9460 Hartree, Solvent: CH_3_OH, Boltzmann %: 24.3); Table S21: Coordinates (Ångstroms) for conformer (2″*S*, 3*R,* 5*S,* 8*R,* 9*R,* 10*S*)-**3**_C3 (Energy: −1748.9433 Hartree, Solvent: CH_3_OH, Boltzmann %: 1.3); Table S22: Coordinates (Ångstroms) for conformer (2″*S*, 3*R,* 5*S,* 8*R,* 9*R,* 10*S*)-**3**_C4 (Energy: −1748.9424 Hartree, Solvent: CH_3_OH, Boltzmann %: 0.5); Figure S67: Structures of the low-energy B3LYP/6-311G(d,p) conformers of A) (2″*S*, 3*R,* 5*S,* 8*R,* 9*R,* 10*S*)–**1**, B) (2″*R*, 10*R*, 10′*R*, 13*R*, 13′*R*, 14*S*, 14′*S*, 15*S*, 15′*S*, 18*R*, 18′*R*)-**2**, and C) (2″*S*, 3*R,* 5*S,* 8*R,* 9*R,* 10*S*)–**3** in CH_3_OH (298 K and 1 atm); Table S23: Molecular network annotations (isolated compounds highlighted in blue).
