# Supplementary material for: New Phenylspirodrimanes from the Sponge-Associated Fungus Stachybotrys chartarum MUT 3308
Source: Mar Drugs. 2023 Feb 21;21(3):135. doi: 10.3390/md21030135 (PMC10053839; doi:10.3390/md21030135)
Supplement: Supplementary file 1 [file marinedrugs-21-00135-s001.zip › marinedrugs-2217085-supplementary.pdf]

Supporting Information

New phenylspirodrimanes from the Sponge-Associated Fungus *Stachybotrys chartarum* MUT 3308

Marie Dayras [1,2], Estelle Sfecci [1,2], Elena Bovio [3,4], Olivia Rastoin [5], Maeva Dufies [5], Fabien Fontaine-Vive [1,2], Elisabeth Taffin-de-Givenchy [1,2], Thierry Lacour [6], Gilles Pages [2,5,7], Giovanna Cristina Varese [3] and Mohamed Mehiri [1,2*]

[1] Marine Natural Products Team, Institut de Chimie de Nice, Université Côte d'Azur, CNRS UMR 7272, 06108 Nice, France; marie.dayras@univ-cotedazur.fr (M.D.); estelle.sfecci@unice.fr (E.S.); mohamed.mehiri@univ-cotedazur.fr (M.M.); fabien.fontaine-vive@univ-cotedazur.fr (F.F.V.); elisabeth.taffin-de-givenchy@univ-cotedazur.fr (E.T.G.)

[2] LIA ROPSE, Laboratoire International Associé, Université Côte d'Azur - Centre Scientifique de Monaco, Nice, France

[3] Mycotheca Universitatis Taurinensis, Department of Life Sciences and Systems Biology, University of Turin, Viale Mattioli 25, 10125 Turin, Italy; elena.bovio@inrae.fr (E.B.); cristina.varese@unito.it (G.C.V.)

[4] UMR Institut Sophia Agrobiotech, INRAE, CNRS, UCA, 400 routes des Chappes, 06903 Sophia Antipolis, France

[5] Institute for Research on Cancer and Aging of Nice, Université Côte d'Azur, CNRS UMR 7284, INSERM U1081, Centre Antoine Lacassagne, 06189 Nice, France; olivia.rastoin@univ-cotedazur.fr (O.R.); maeva.dufies@univ-cotedazur.fr (M.Du.); gilles.pages@univ-cotedazur.fr (G.P.)

[6] Parc d'activités ArômaGrasse/Immeuble GrasseBiotech, 45 boulevard Marcel Pagnol, 06130 Grasse, France; tlacour@biopreserv.fr (T.L.)

[7] Department of Biomedical, Centre Scientifique de Monaco, Monaco, Principality of Monaco

* Correspondence: mohamed.mehiri@univ-cotedazur.fr; Tel.: +33 4 89 15 01 57 (M.M.)

1. Experimental spectra

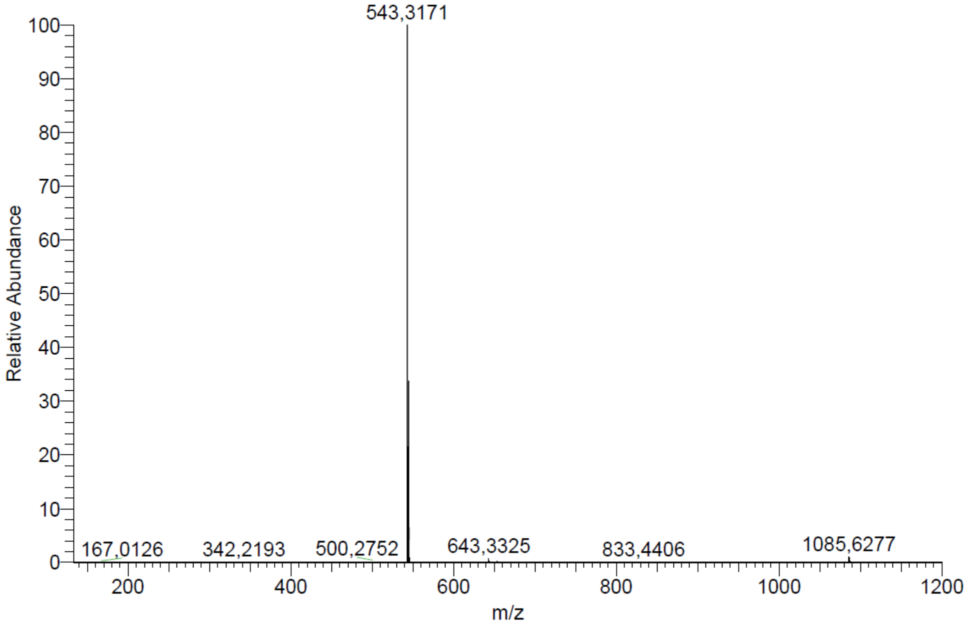

Figure S1. HRESI(+)MS spectrum of stachybotrin J (**1**).

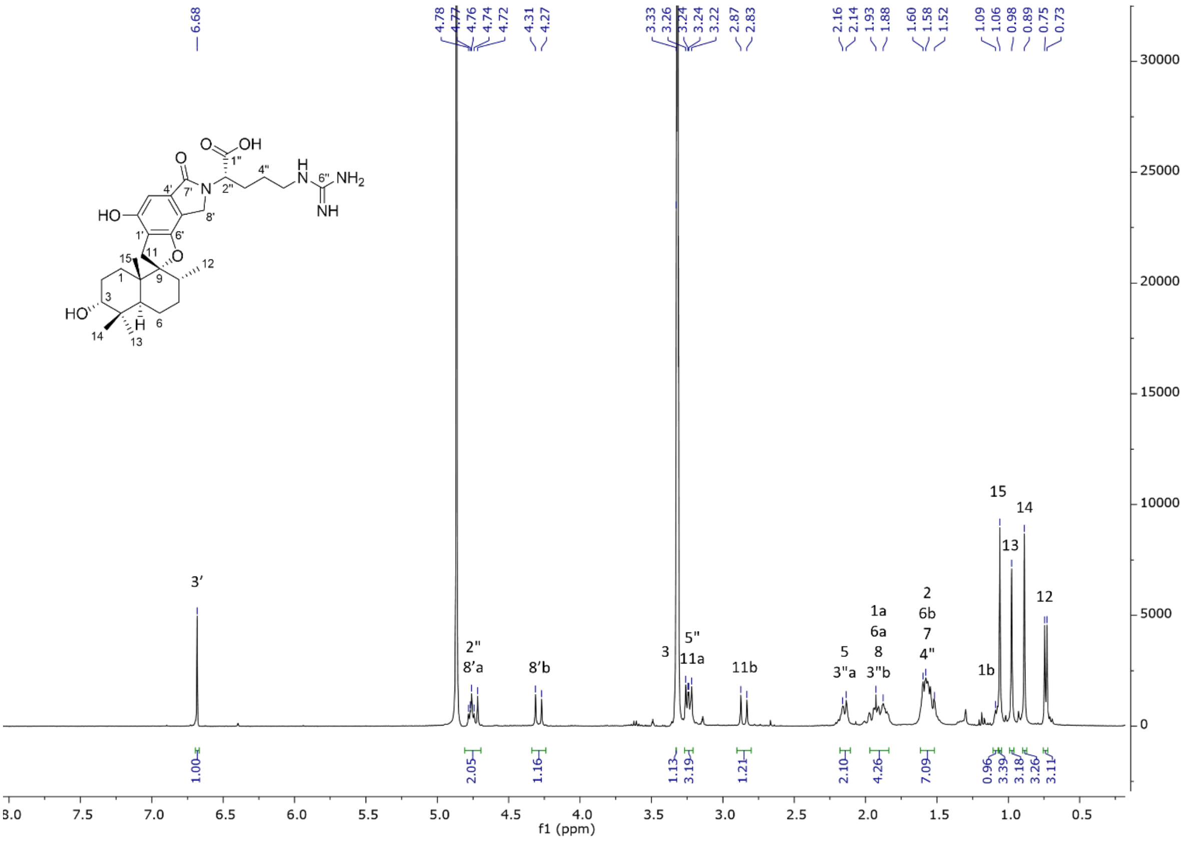

Figure S2. ^{1}H NMR spectrum (500 MHz, CD_3OD) of stachybotrin J (**1**).

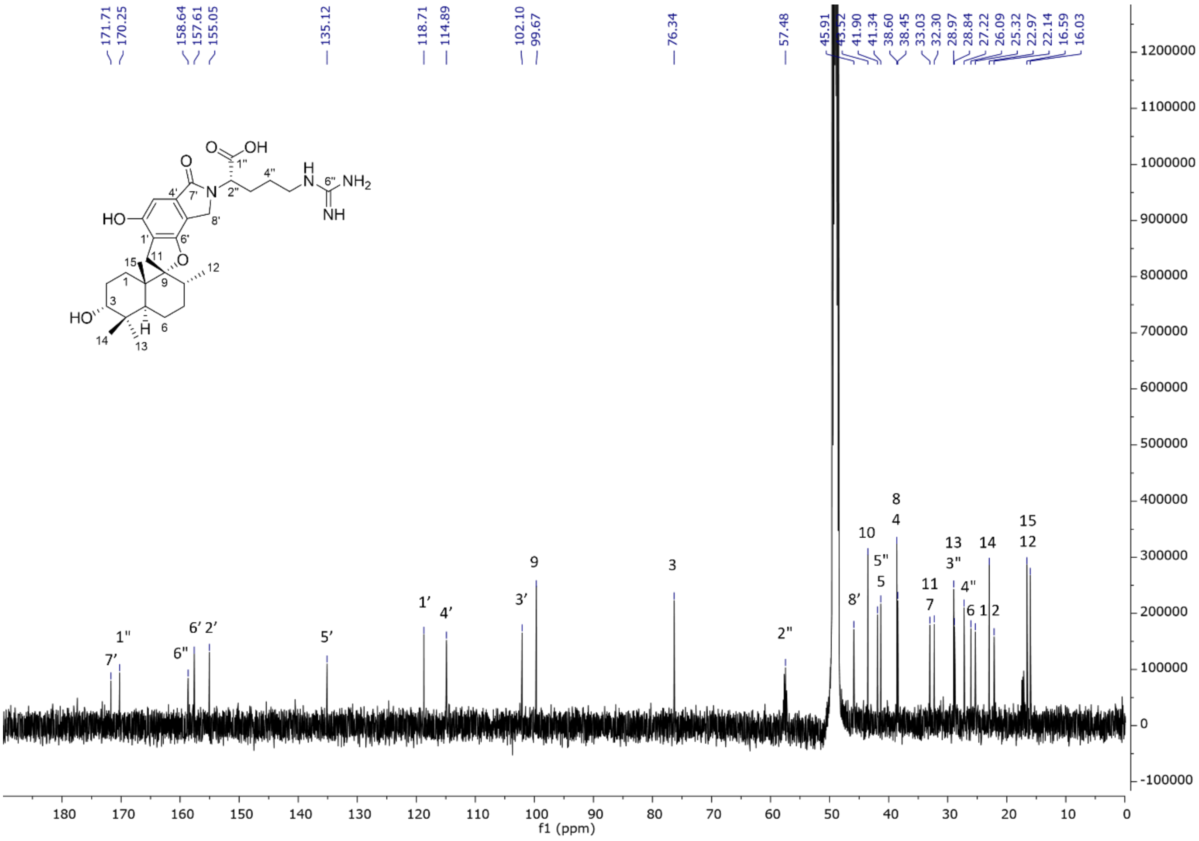

Figure S3. ^{13}C NMR spectrum (125 MHz, CD_3OD) of stachybotrin J (**1**).

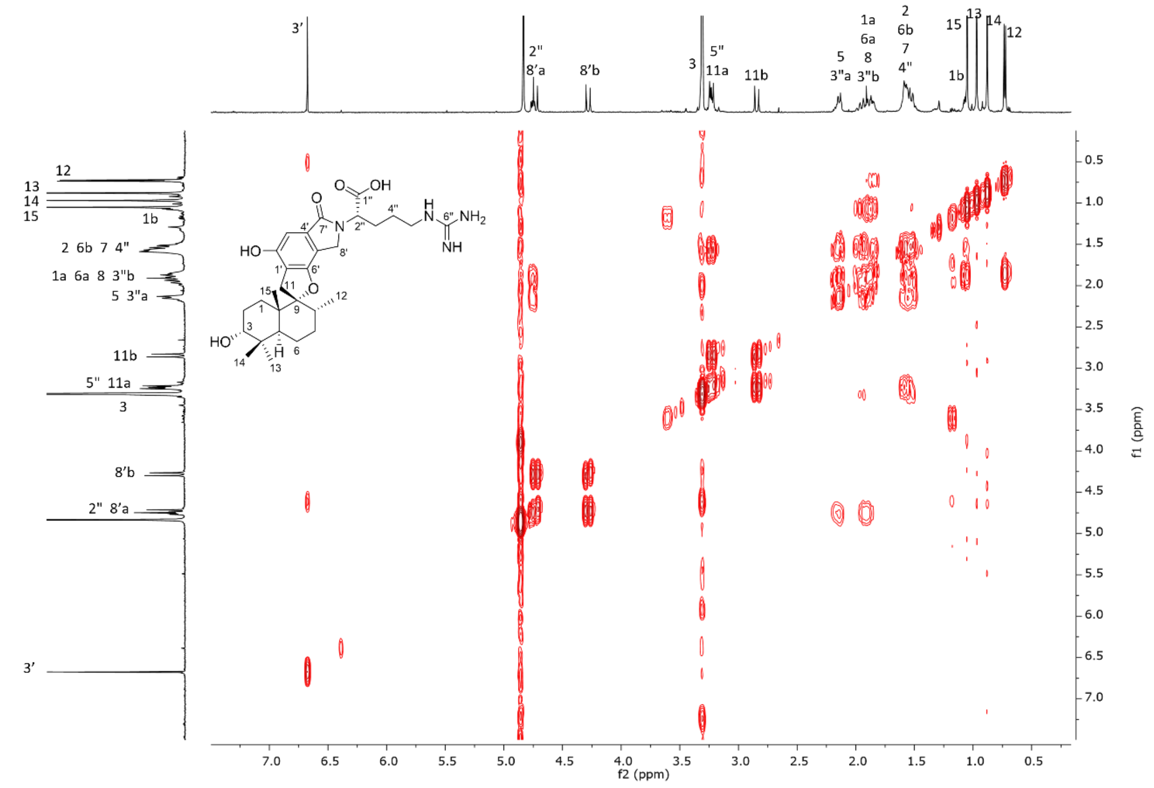

Figure S4. ^{1}H-^{1}H COSY NMR spectrum (500/500 MHz, CD_3OD) of stachybotrin J (**1**).

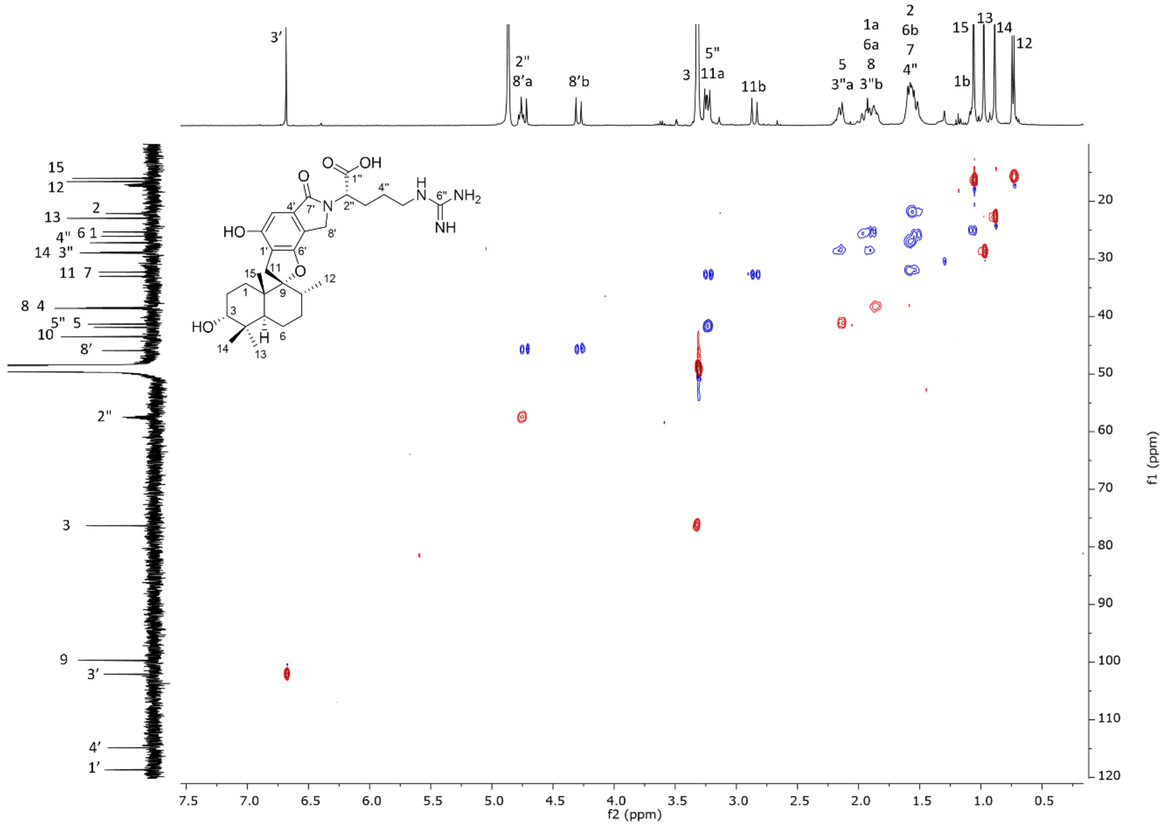

Figure S5. ^{1}H-^{13}C HSQC NMR spectrum (500/125 MHz, CD_3OD) of stachybotrin J (**1**).

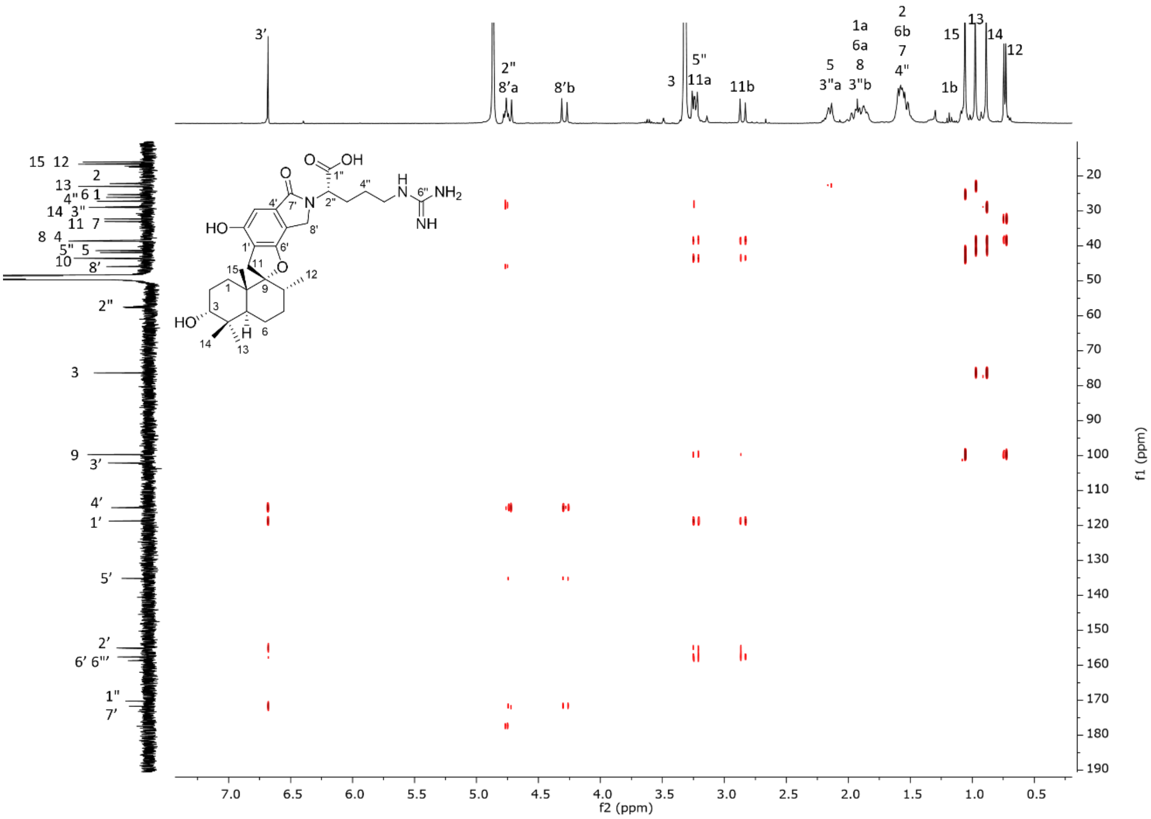

Figure S6. ^{1}H-^{13}C HMBC NMR spectrum (500/125 MHz, CD_3OD) of stachybotrin J (**1**).

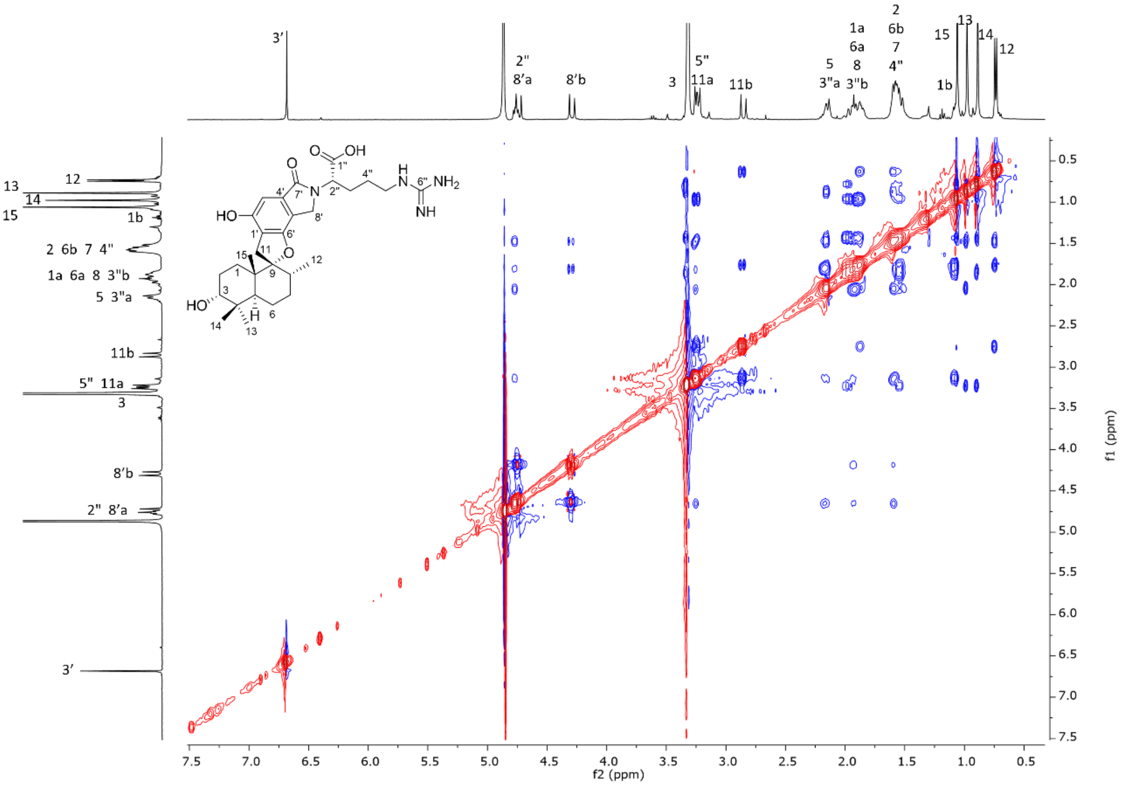

Figure S7. ^{1}H-^{1}H NOESY NMR spectrum (500/500 MHz, CD_3OD) of stachybotrin J (**1**).

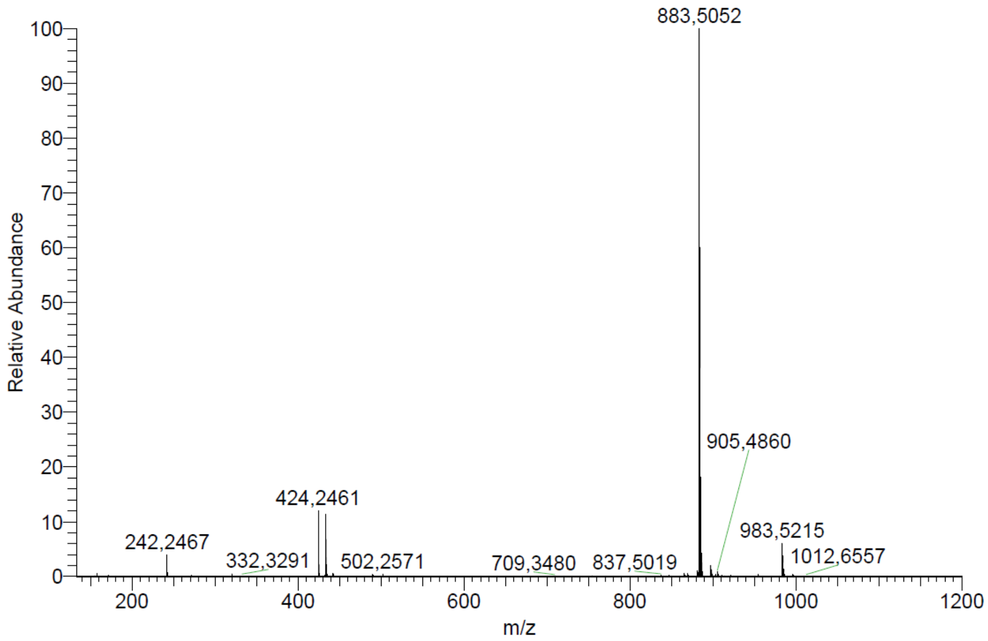

Figure S8. HRESI(+)MS spectrum of stachybocin G (**2**).

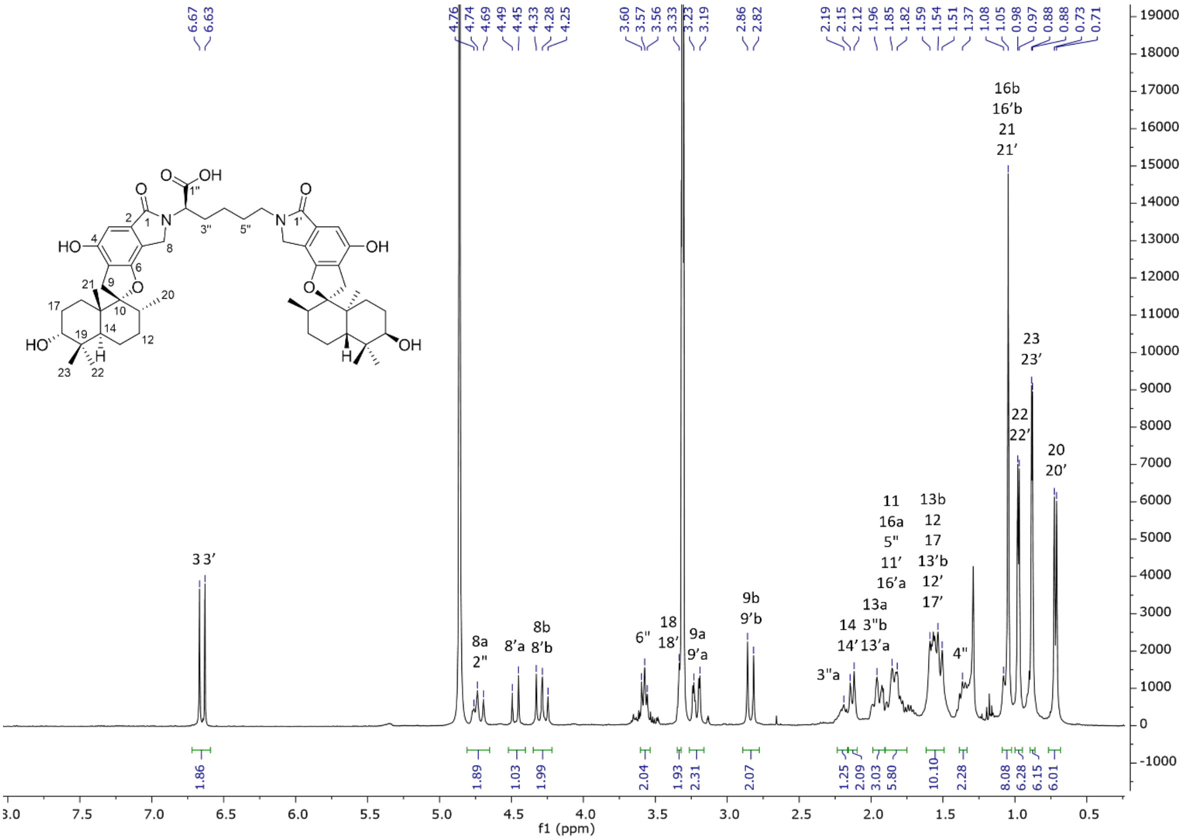

Figure S9. ^{1}H NMR spectrum (500 MHz, CD_3OD) of stachybocin G (**2**).

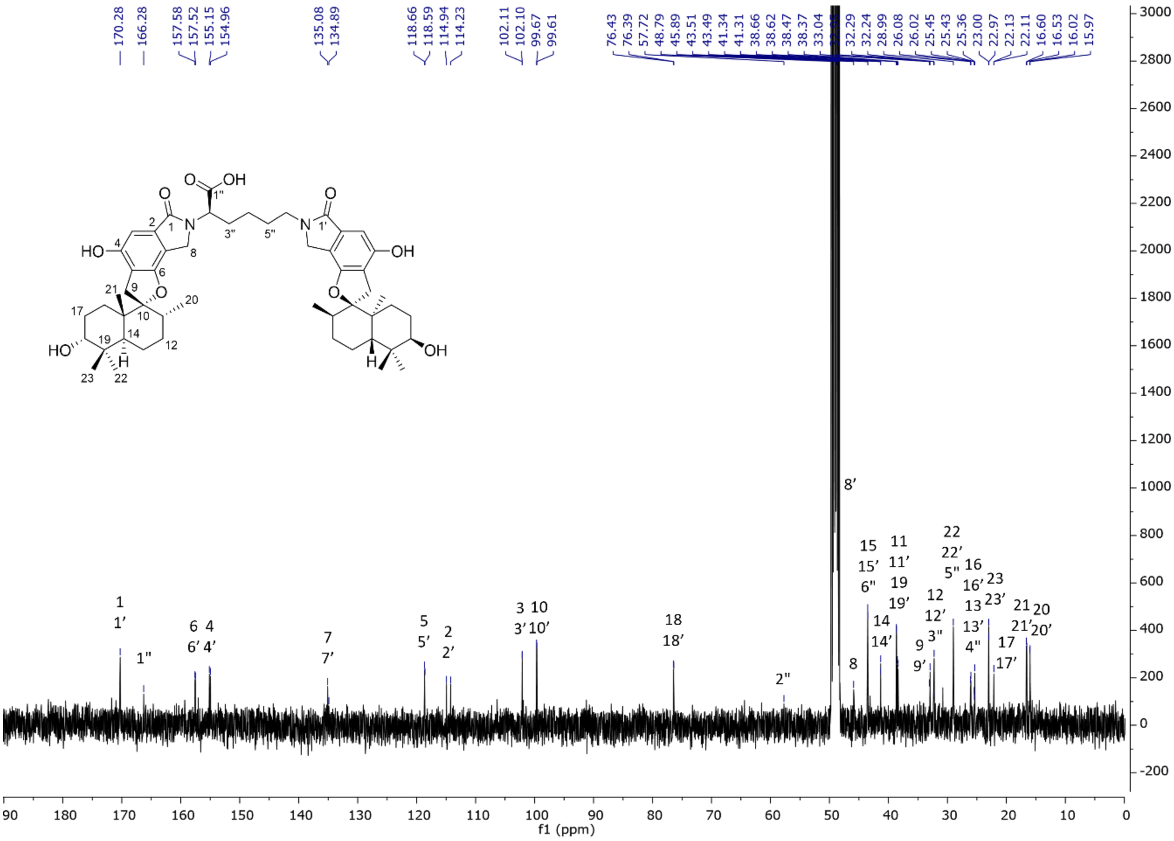

Figure S10. ^{13}C NMR spectrum (125 MHz, CD_3OD) of stachybocin G (**2**).

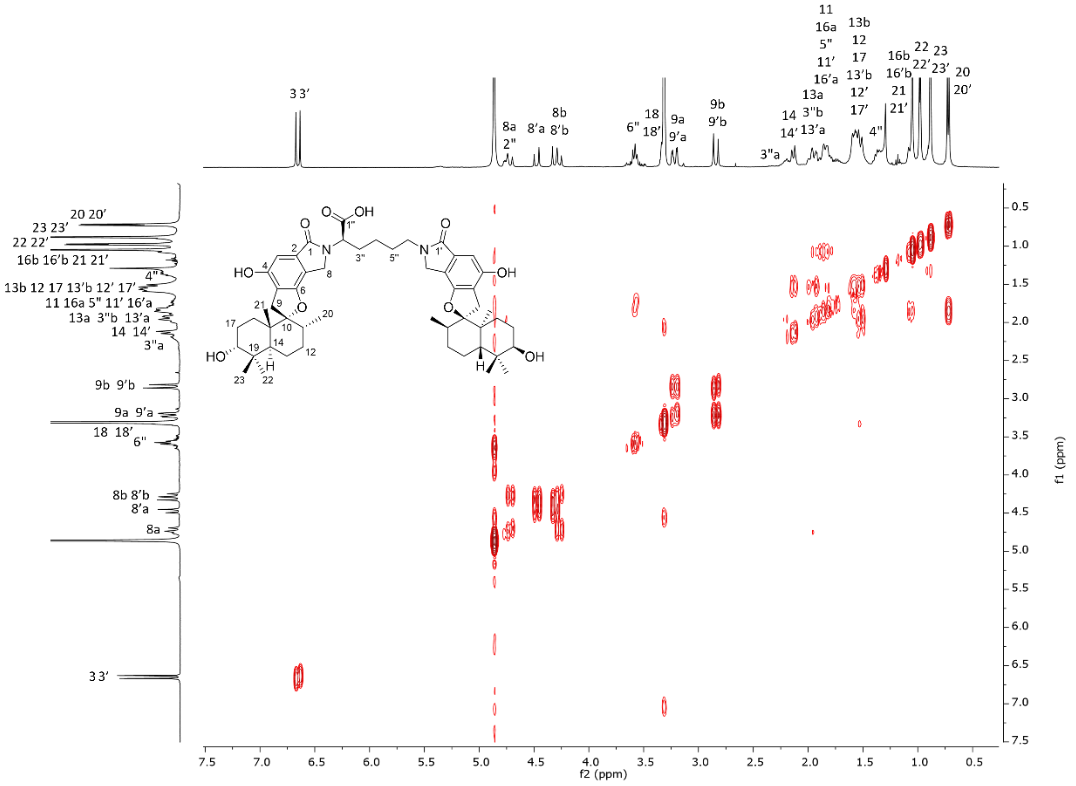

Figure S11. ^{1}H-^{1}H COSY NMR spectrum (500/500 MHz, CD_3OD) of stachybocin G (**2**).

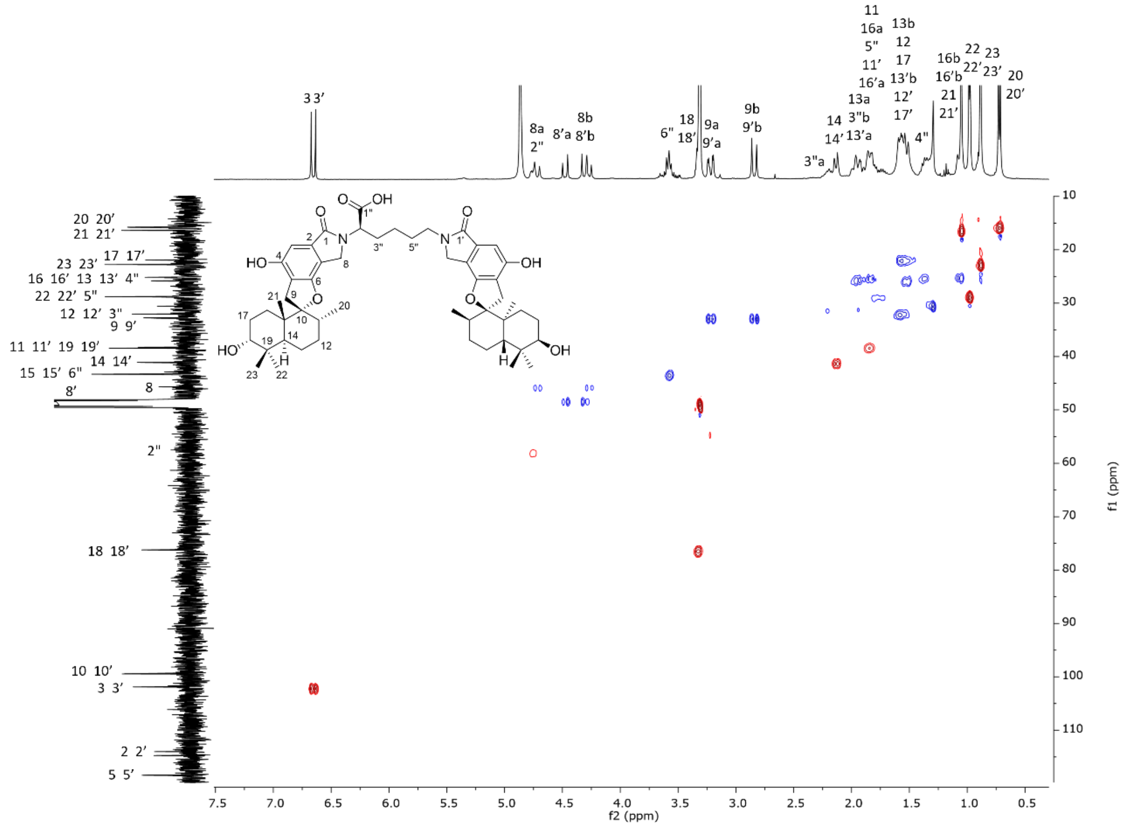

Figure S12. ^{1}H-^{13}C HSQC NMR spectrum (500/125 MHz, CD_3OD) of stachybocin G (**2**).

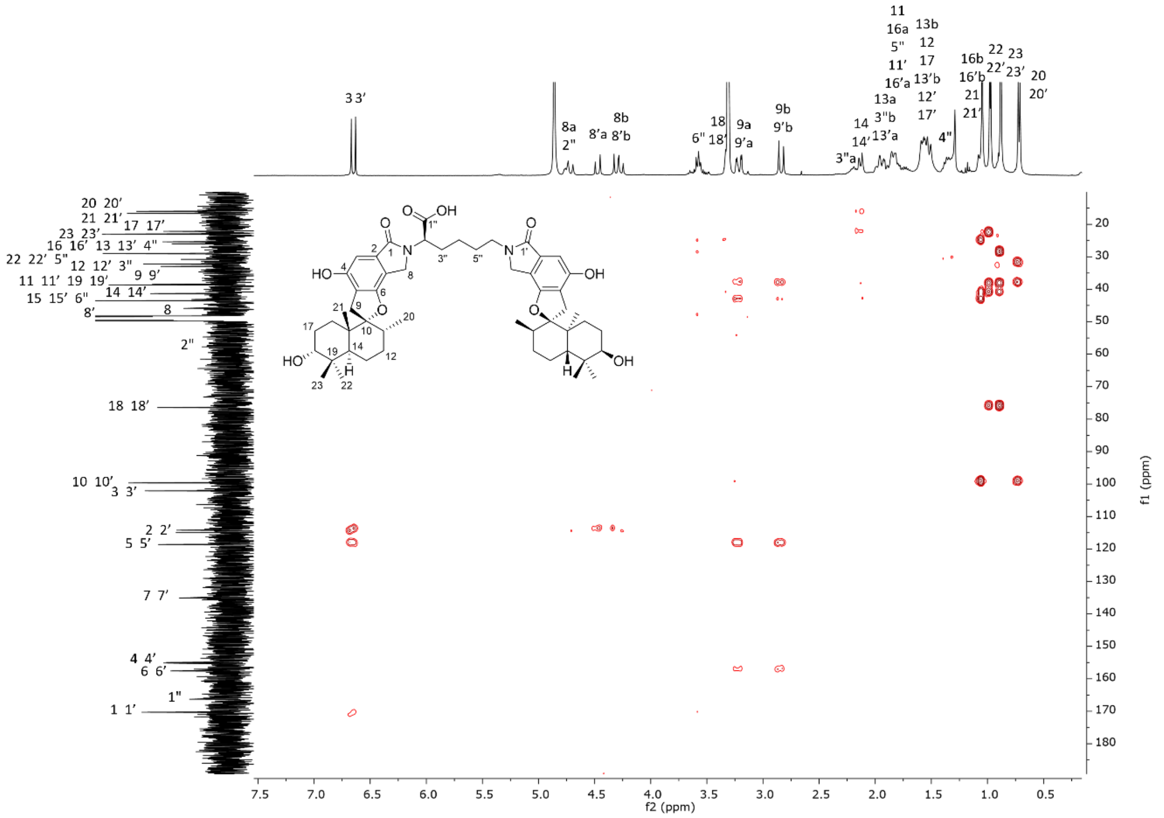

Figure S13. ^{1}H-^{13}C HMBC NMR spectrum (500/125 MHz, CD_3OD) of stachybocin G (**2**).

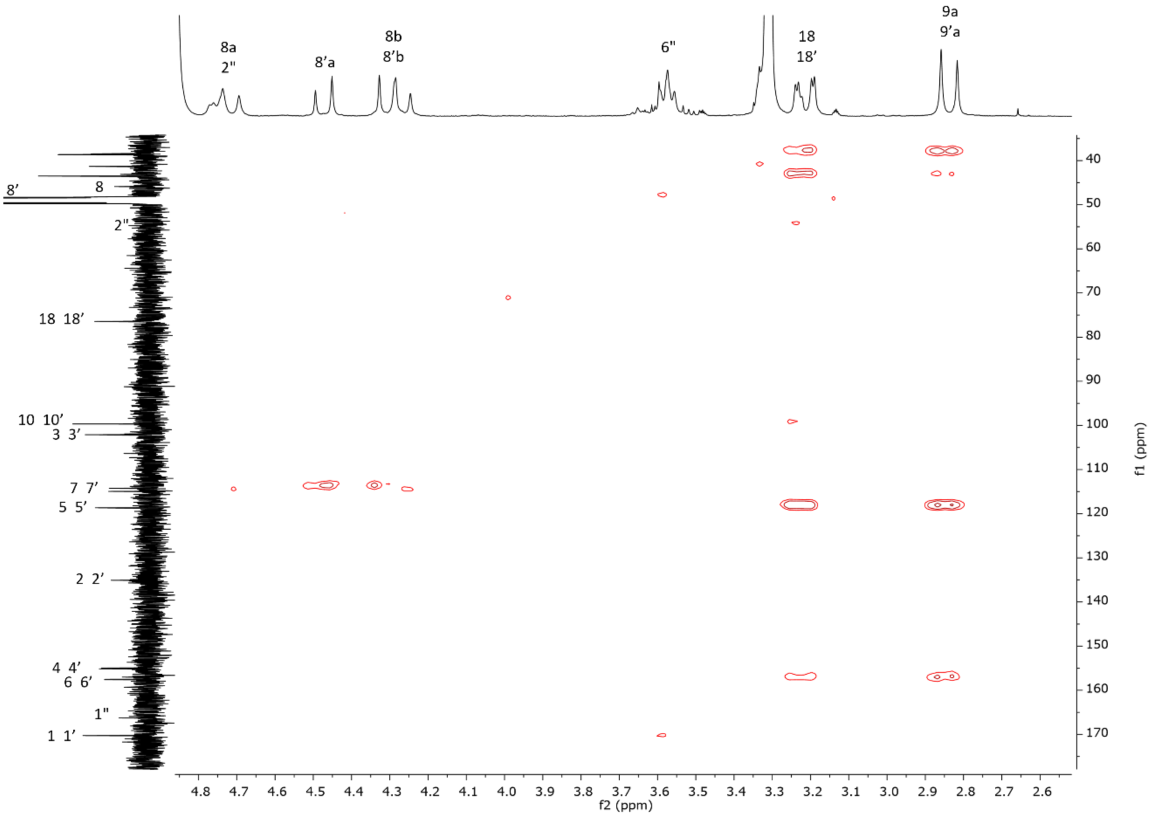

Figure S14. Zoom of the ^{1}H-^{13}C HMBC NMR spectrum (500/125 MHz, CD_3OD) of stachybocin G (**2**).

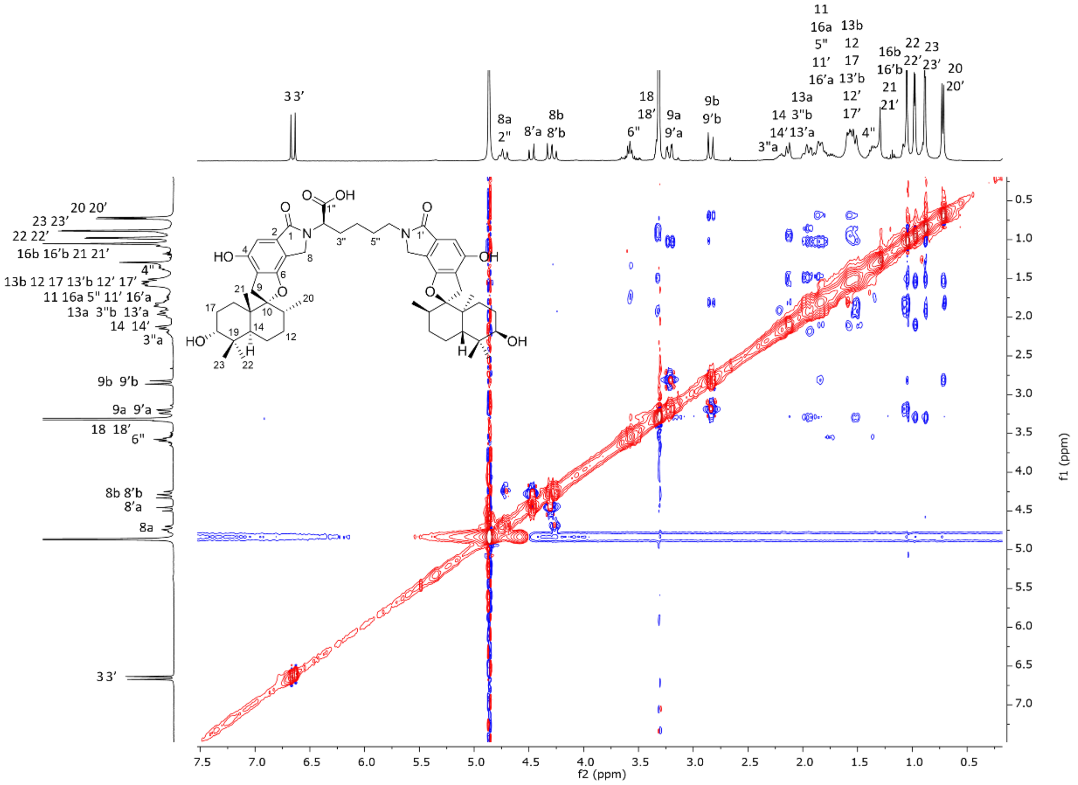

Figure S15. ^{1}H-^{1}H NOESY NMR spectrum (500/500 MHz, CD_3OD) of stachybocin G (**2**).

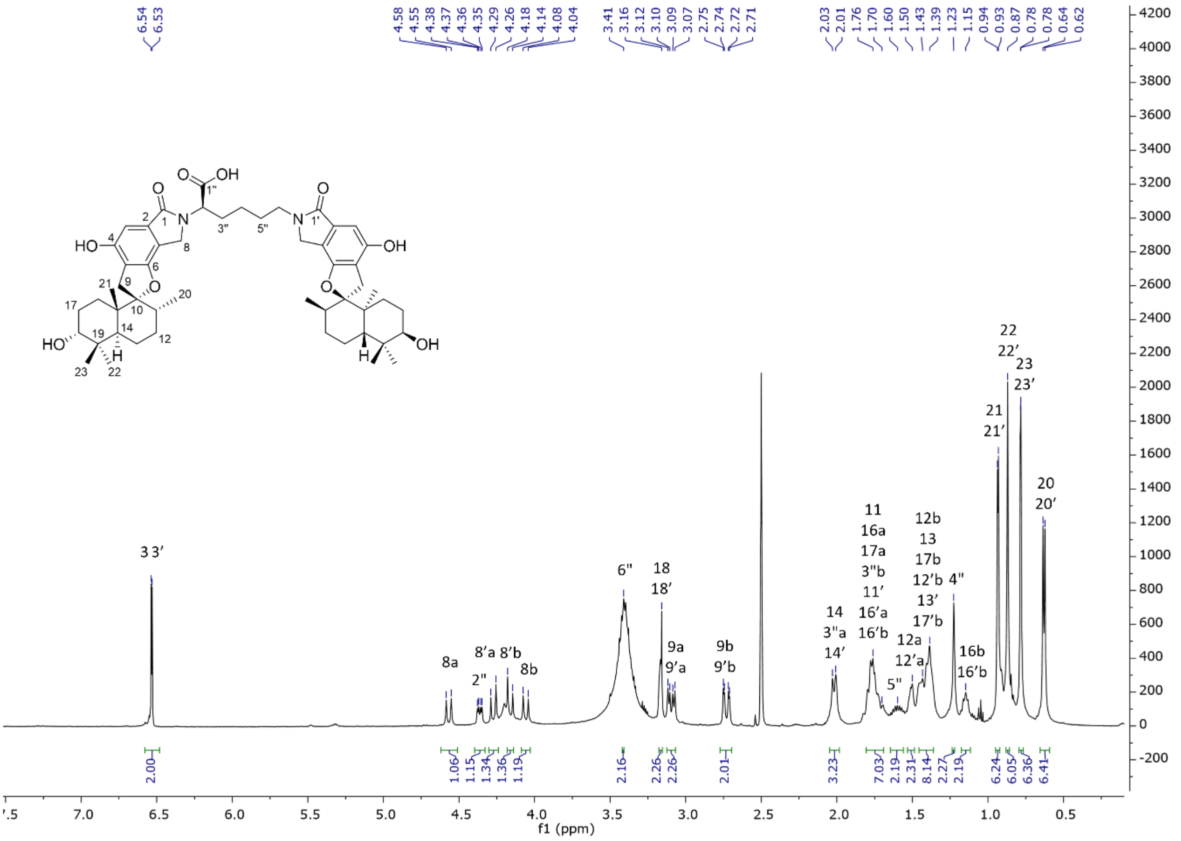

Figure S16. ^{1}H NMR spectrum (500 MHz, $(CD_3)_2SO$) of stachybocin G (**2**).

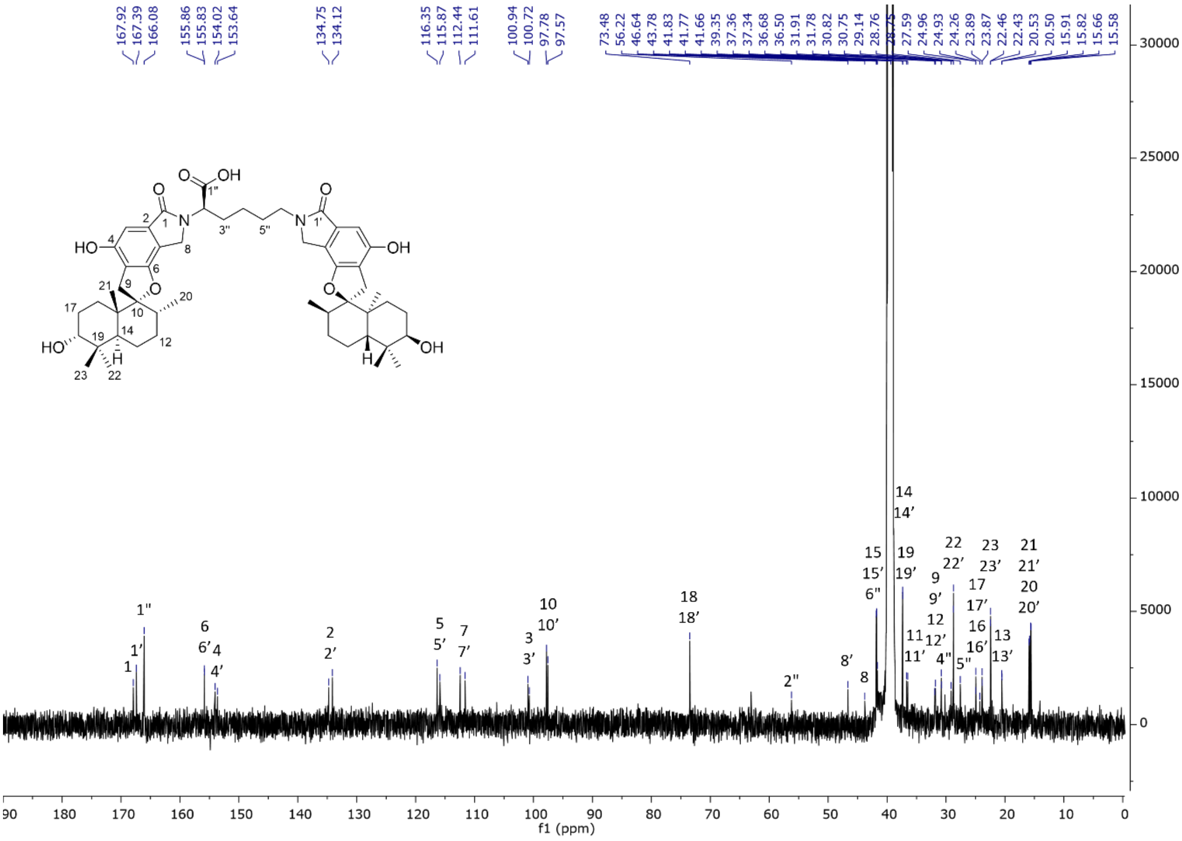

Figure S17. ^{13}C NMR spectrum (125 MHz, $(CD_3)_2SO$) of stachybocin G (**2**).

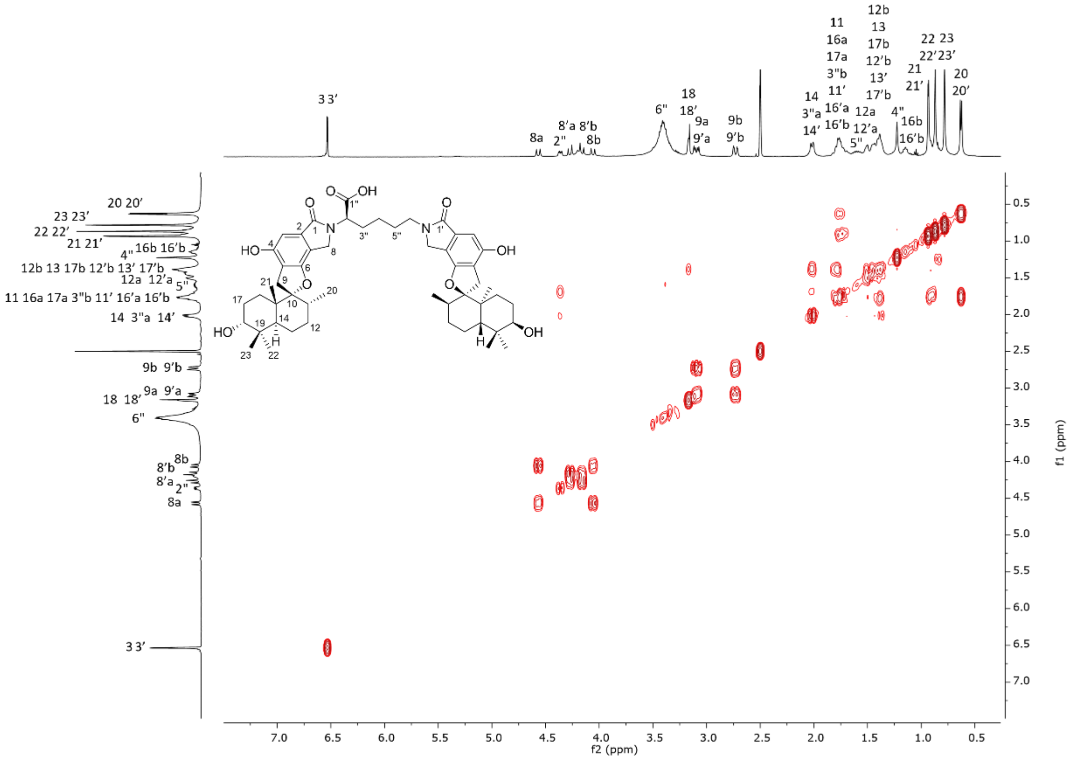

Figure S18. ^{1}H-^{1}H COSY NMR spectrum (500/500 MHz, $(CD_3)_2SO$) of stachybocin G (**2**).

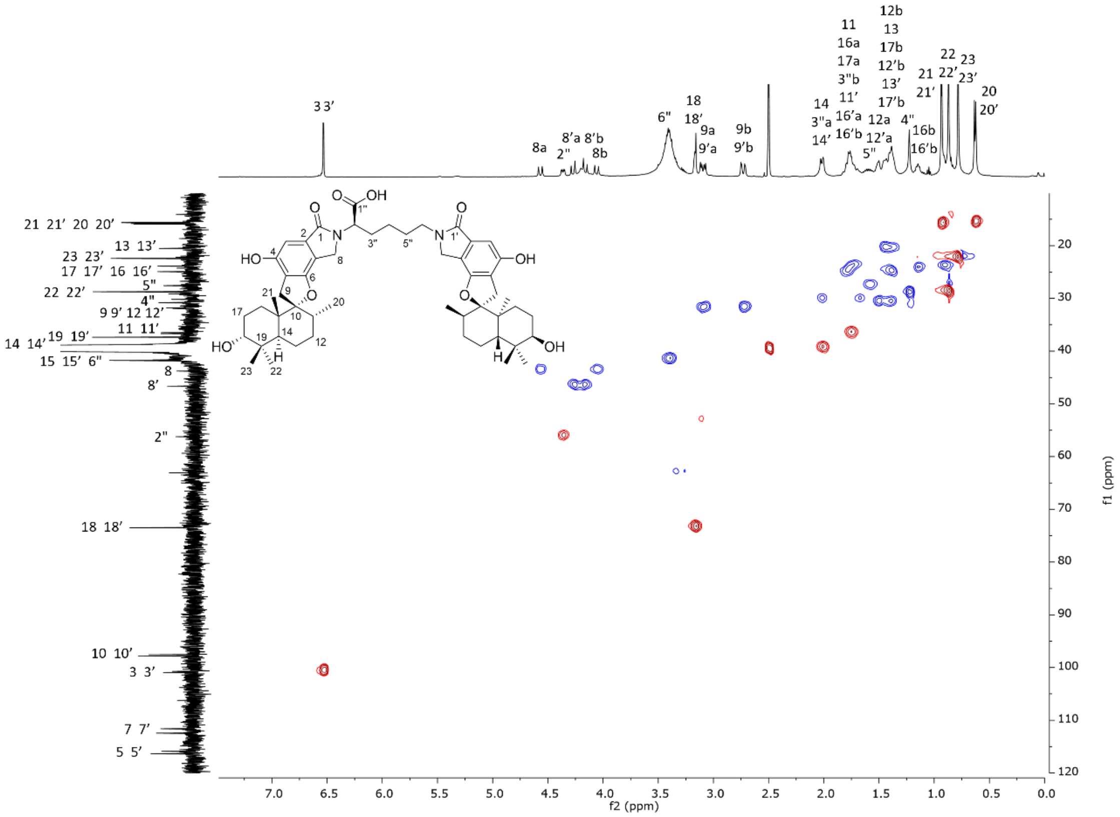

Figure S19. ^{1}H-^{13}C HSQC NMR spectrum (500/125 MHz, $(CD_3)_2SO$) of stachybocin G (**2**).

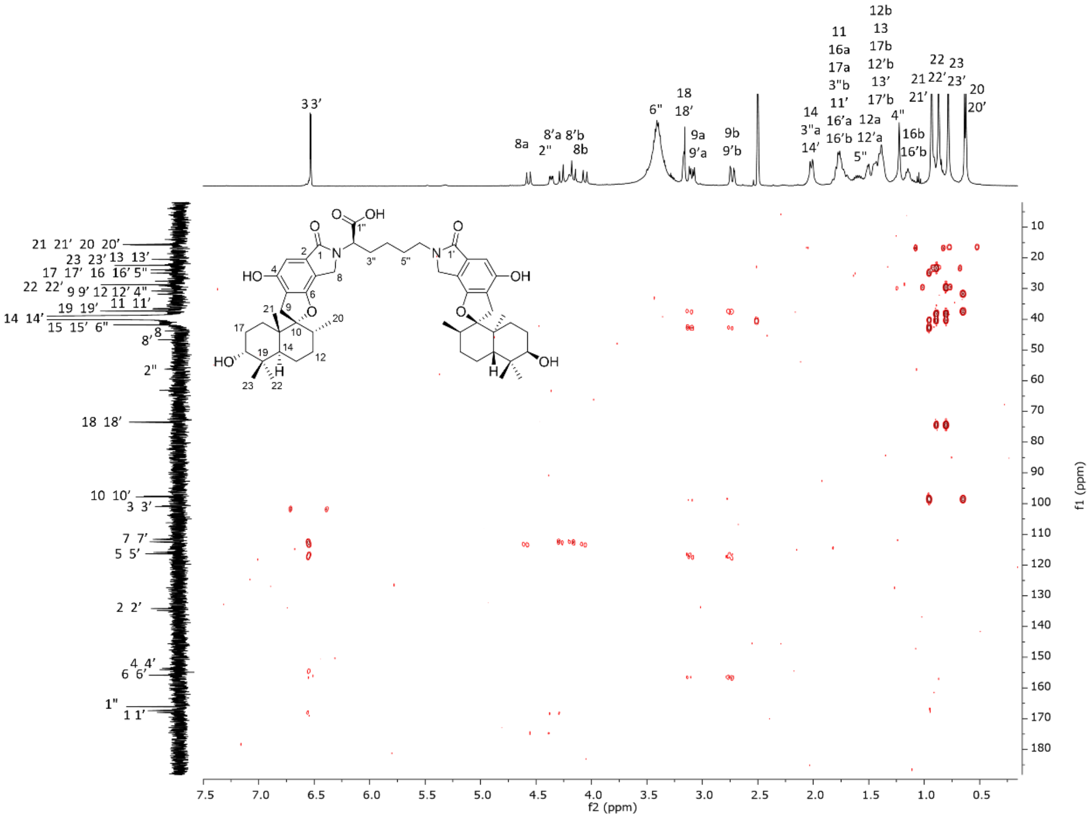

Figure S20. ^{1}H-^{13}C HMBC NMR spectrum (500/125 MHz, $(CD_3)_2SO$) of stachybocin G (**2**).

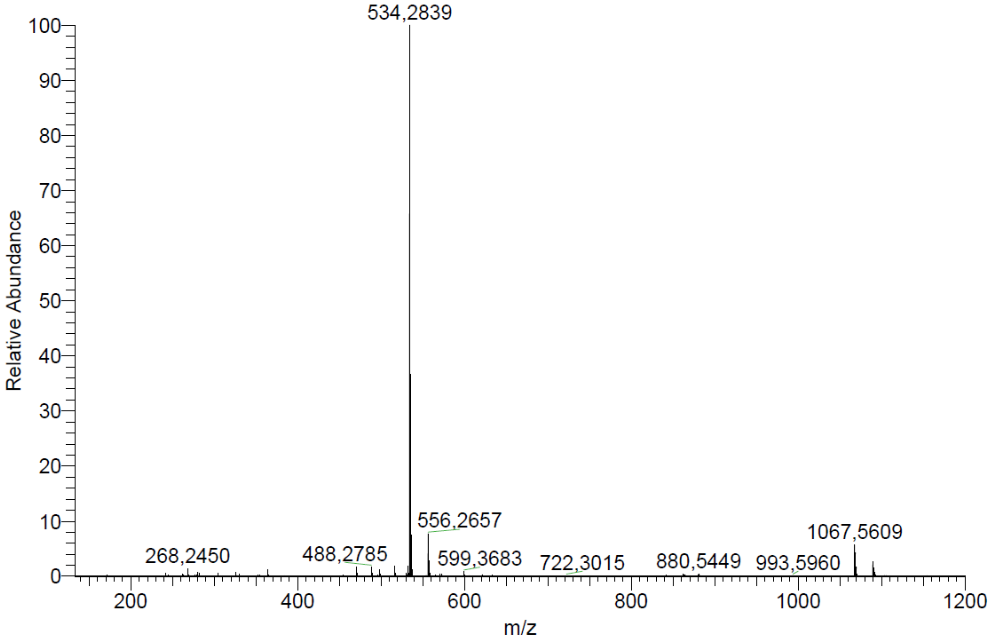

Figure S21. HRESI(+)MS spectrum of stachybotrin I (**3**).

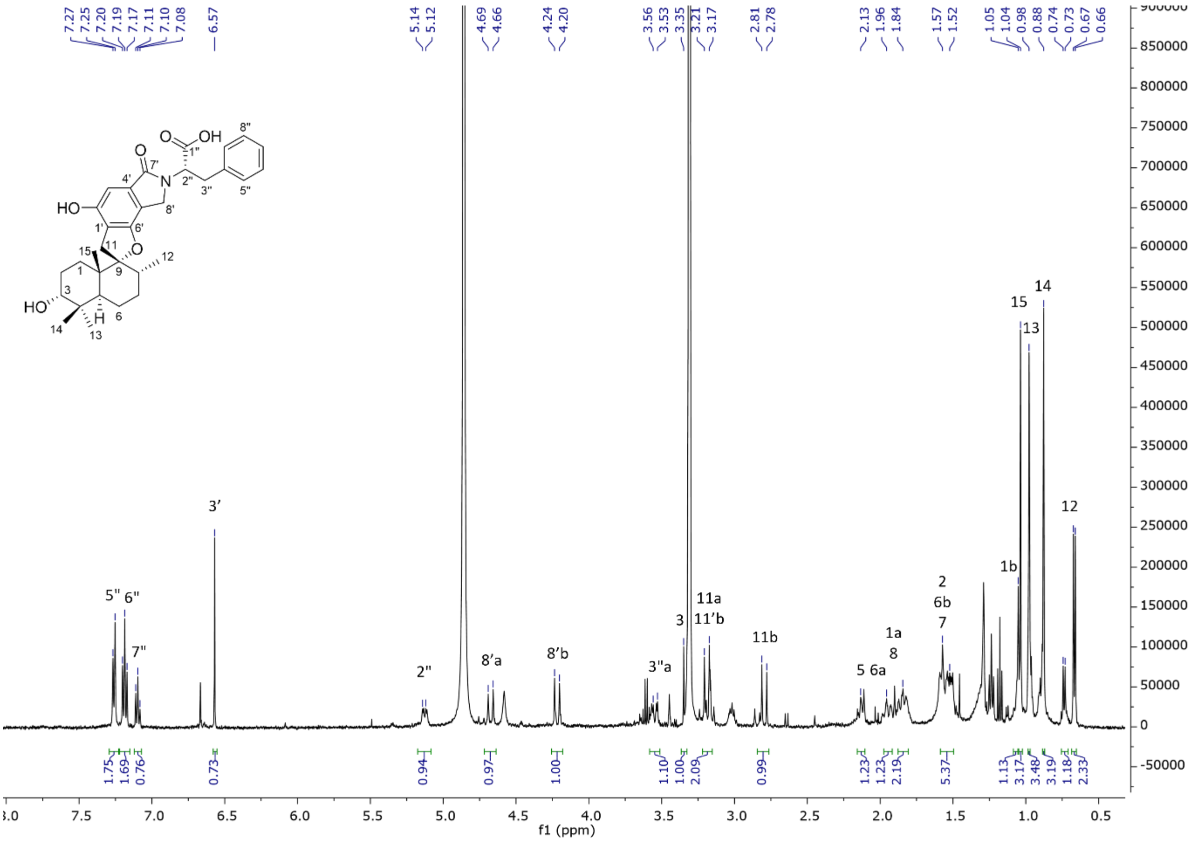

Figure S22. ^{1}H NMR spectrum (500 MHz, CD_3OD) of stachybotrin I (**3**).

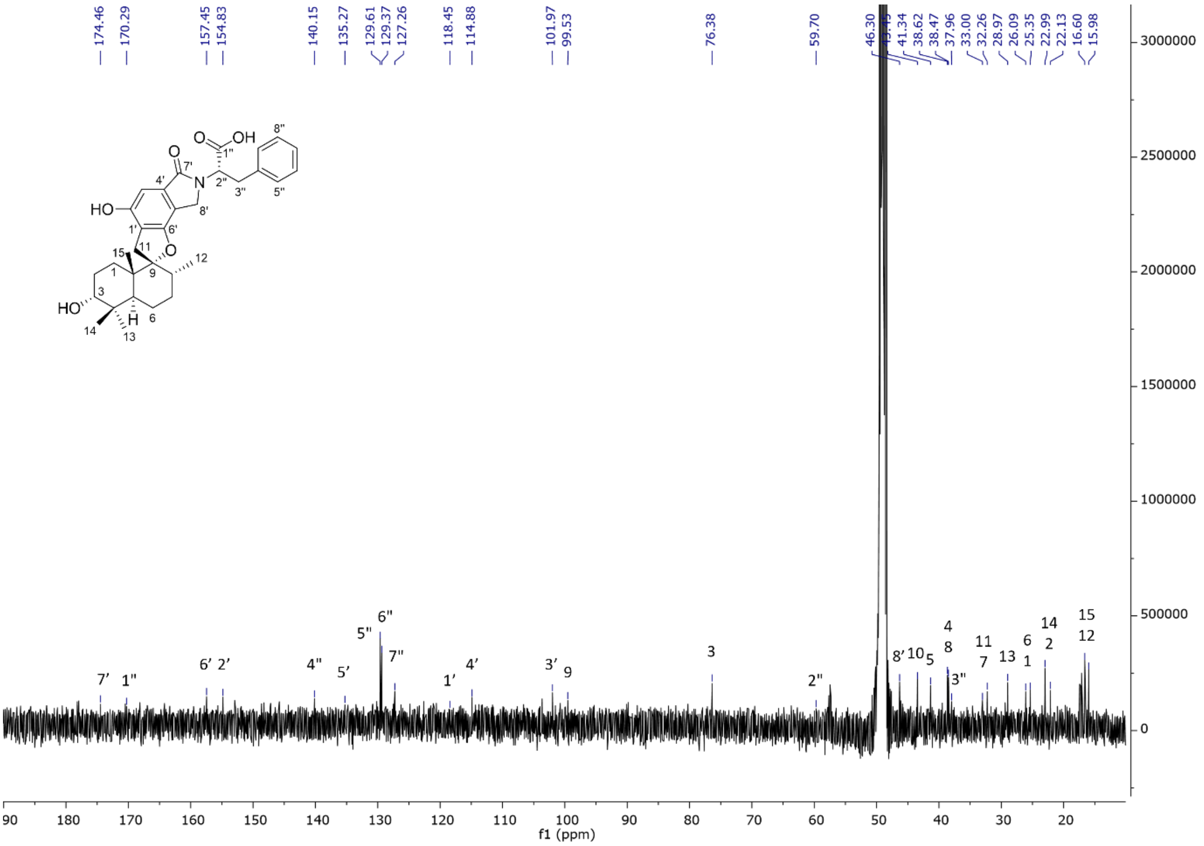

Figure S23. ^{13}C NMR spectrum (125 MHz, CD_3OD) of stachybotrin I (**3**).

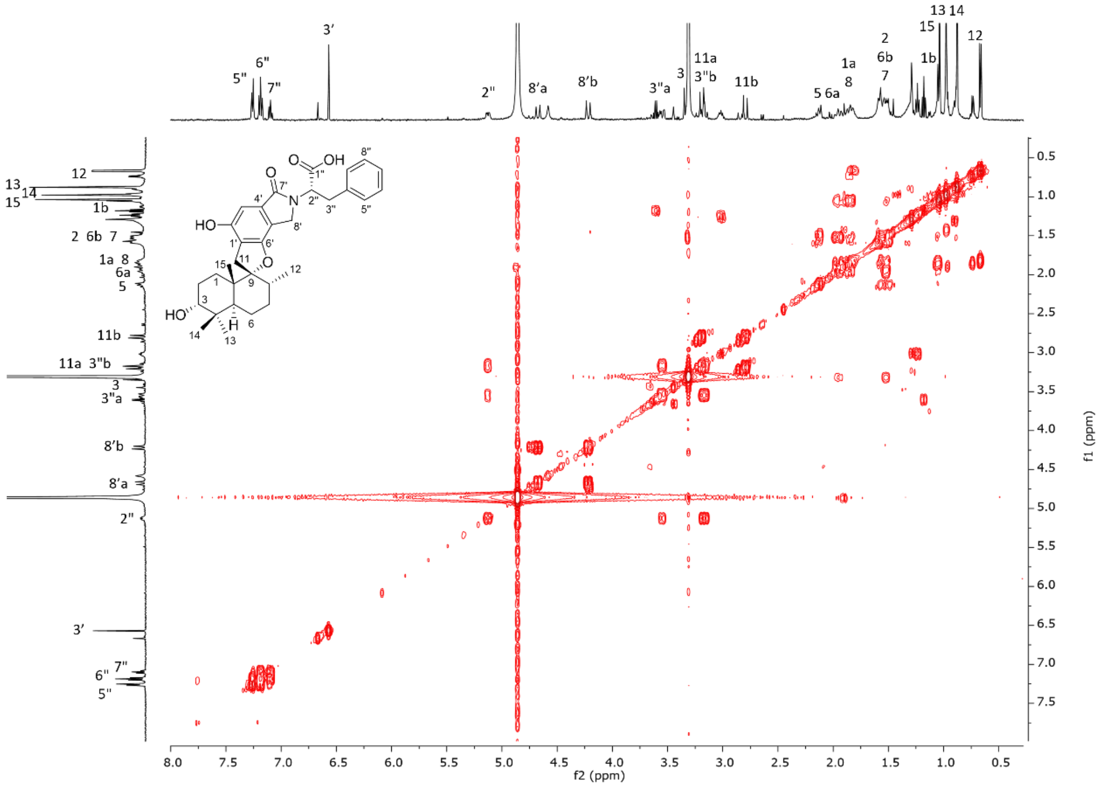

Figure S24. ^{1}H-^{1}H COSY NMR spectrum (500/500 MHz, CD_3OD) of stachybotrin I (**3**).

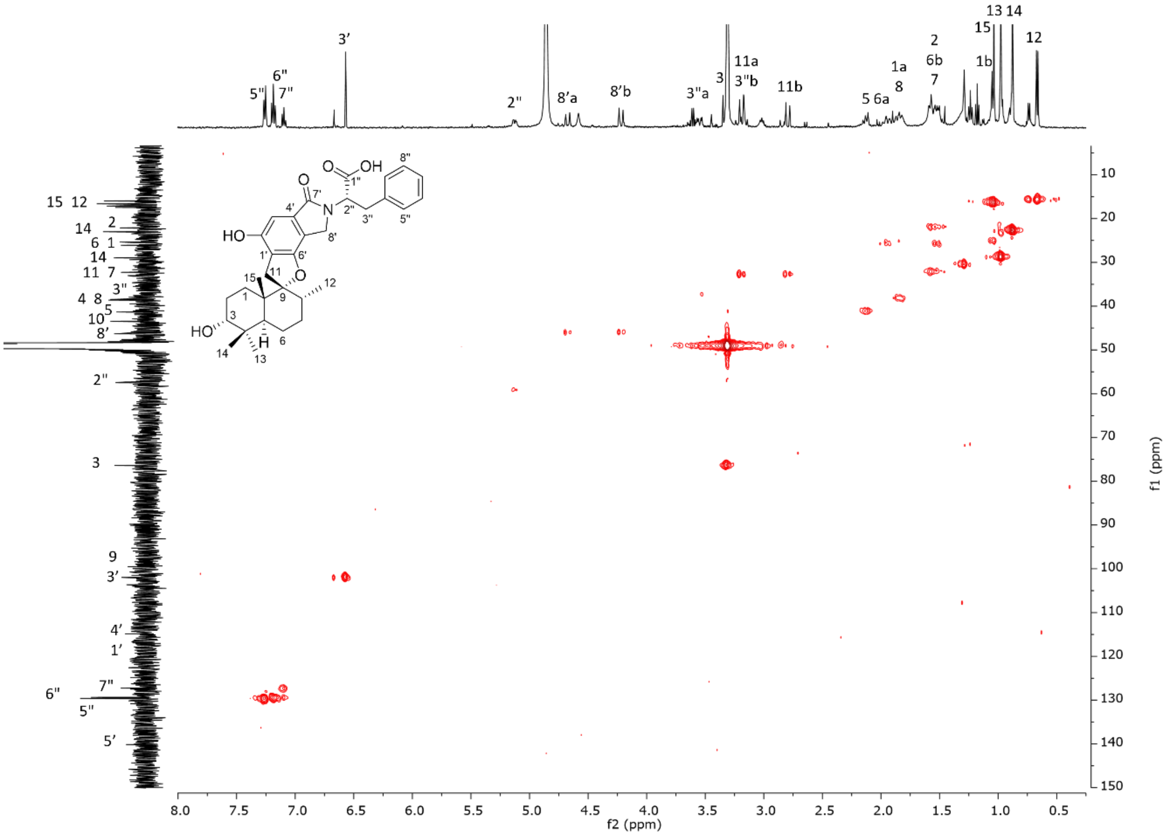

Figure S25. ^{1}H-^{13}C HSQC NMR spectrum (500/125 MHz, CD_3OD) of stachybotrin I (**3**).

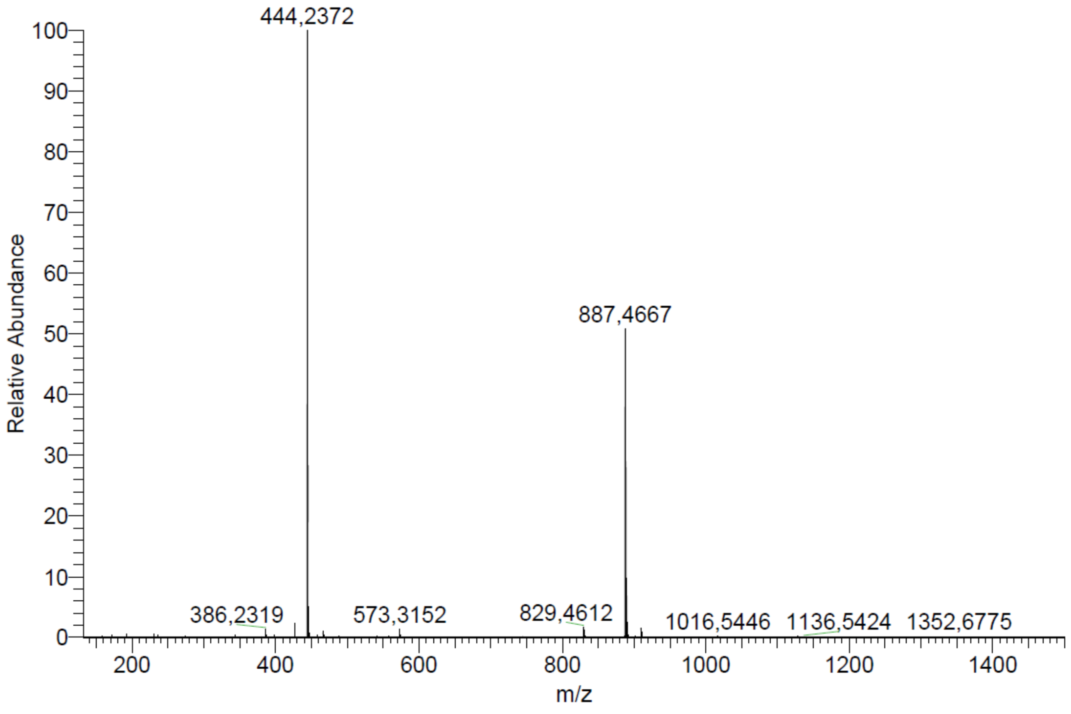

Figure S26. HRESI(+)MS spectrum of stachybotrin H (**4**).

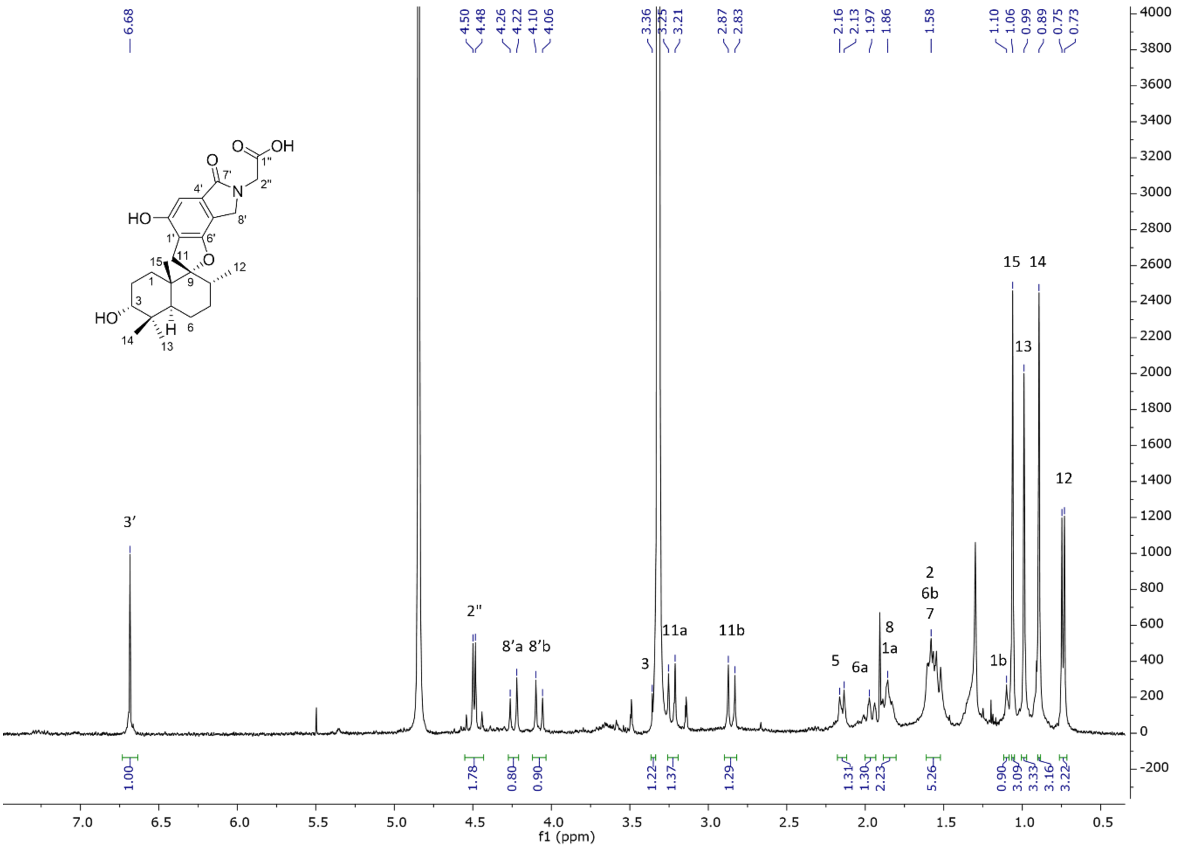

Figure S27. ^{1}H NMR spectrum (500 MHz, CD_3OD) of stachybotrin H (**4**).

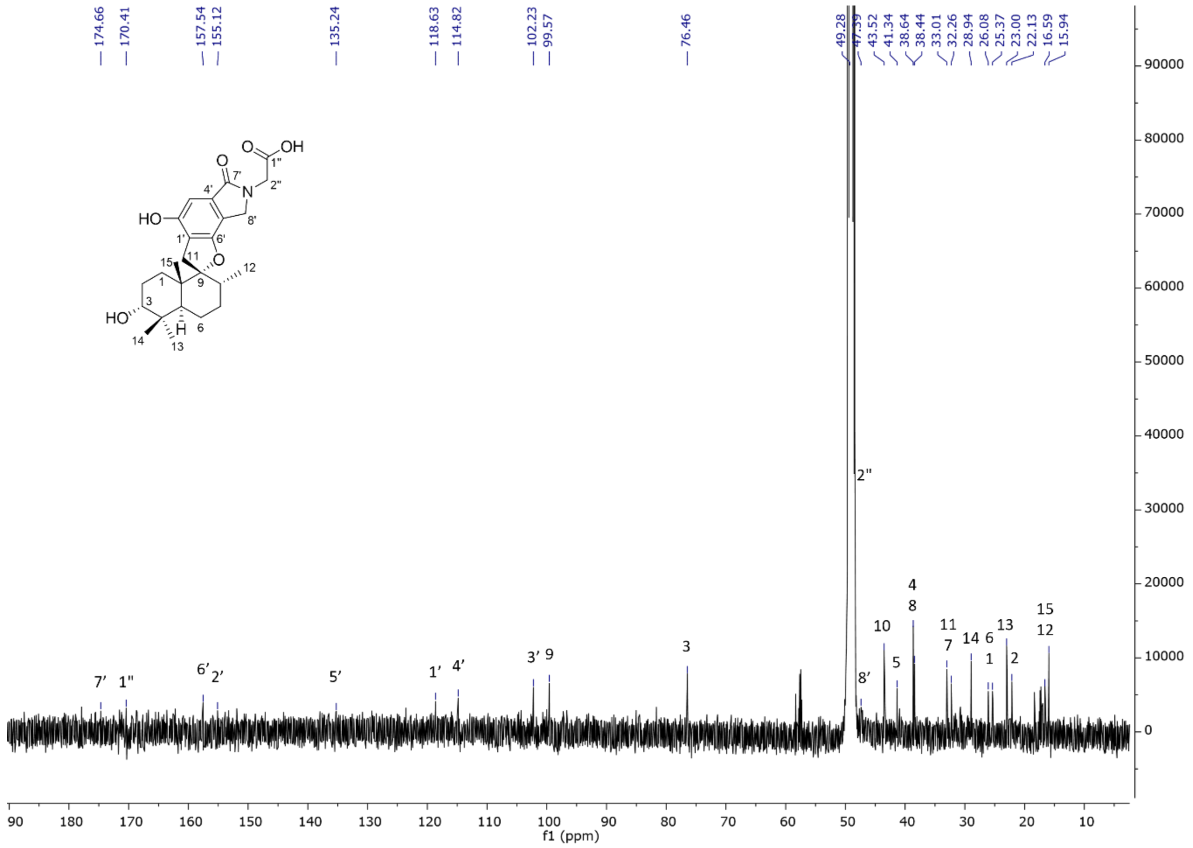

Figure S28. ^{13}C NMR spectrum (125 MHz, CD_3OD) of stachybotrin H (**4**).

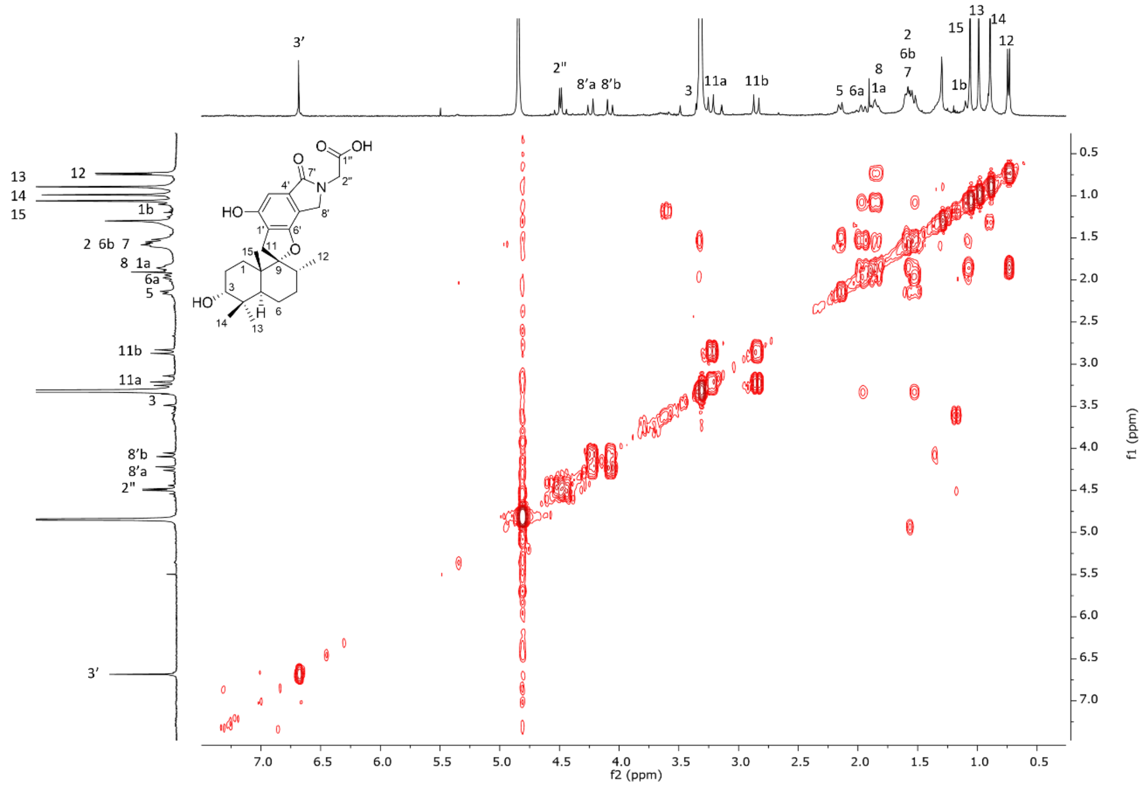

Figure S29. ^{1}H-^{1}H COSY NMR spectrum (500/500 MHz, CD_3OD) of stachybotrin H (**4**).

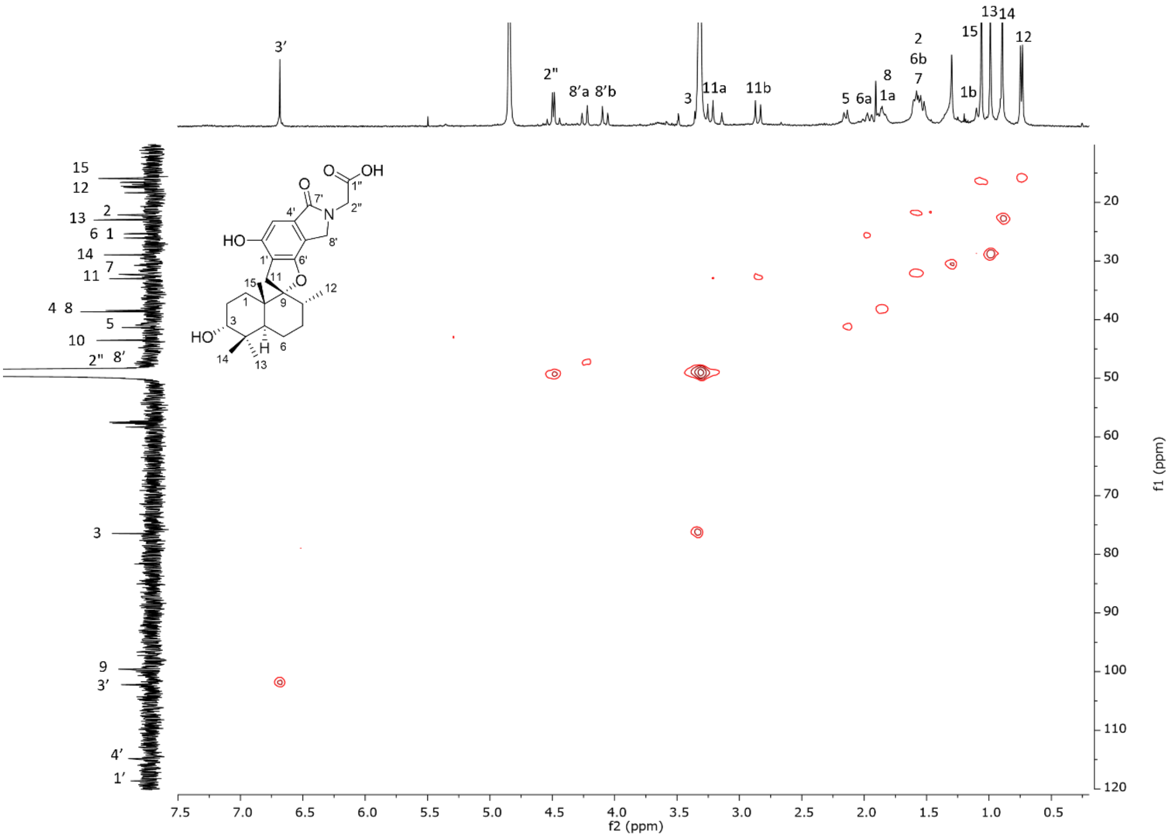

Figure S30. ^{1}H-^{13}C HSQC NMR spectrum (500/125 MHz, CD_3OD) of stachybotrin H (**4**).

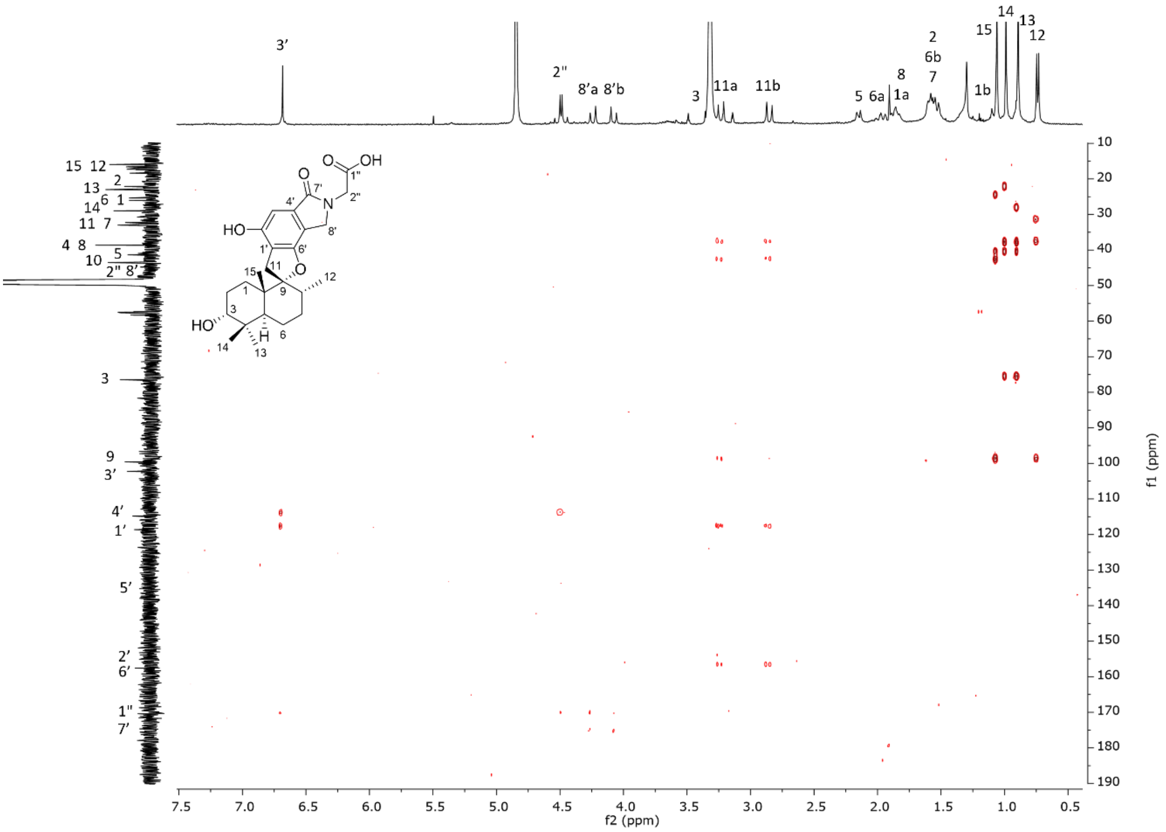

Figure S31. ^{1}H-^{13}C HMBC NMR spectrum (500/125 MHz, CD_3OD) of stachybotrin H (**4**).

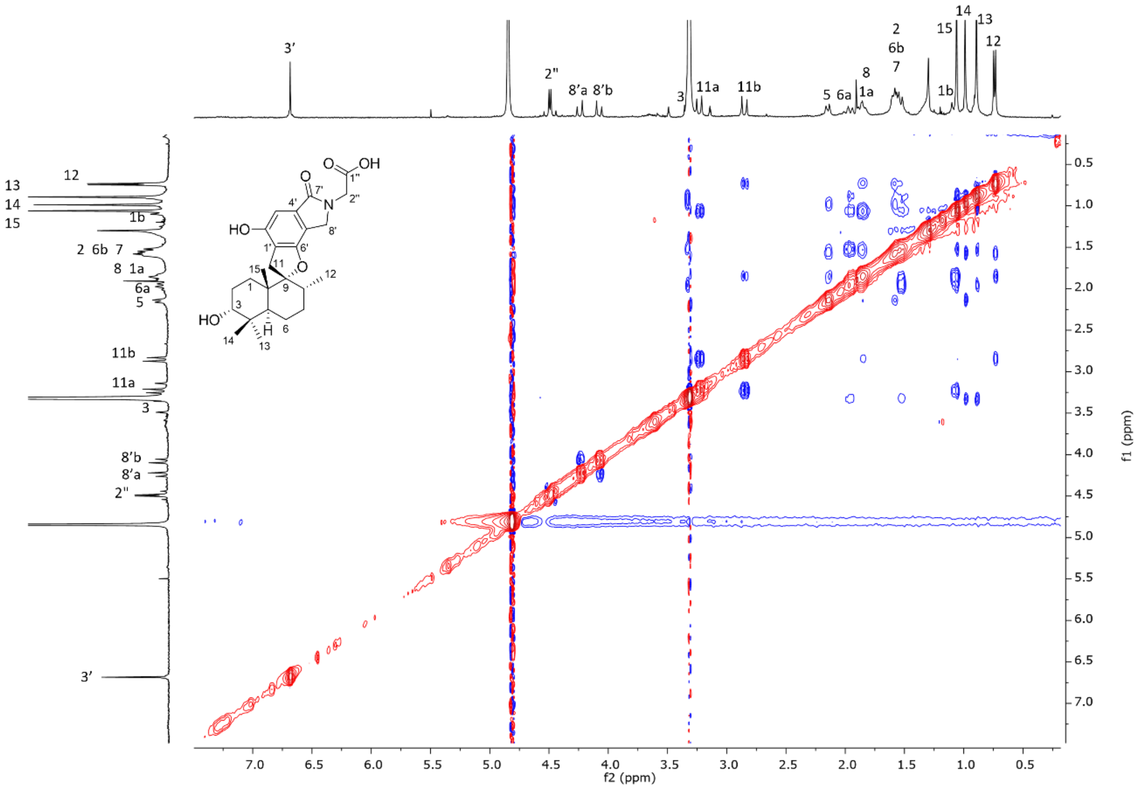

Figure S32. ^{1}H-^{1}H NOESY NMR spectrum (500/500 MHz, CD_3OD) of stachybotrin H (**4**).

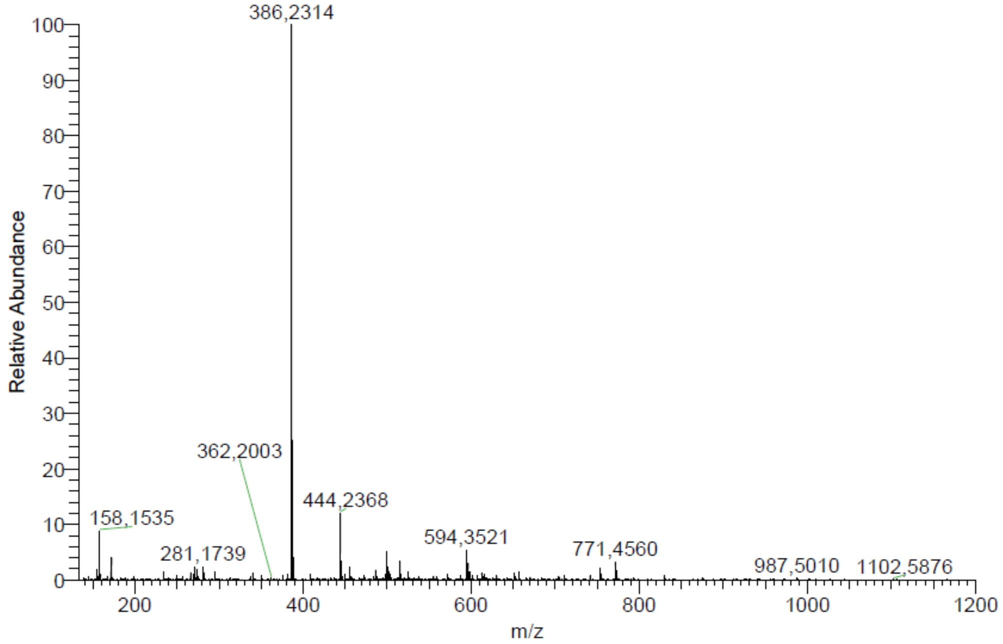

Figure S33. HRESI(+)MS spectrum of stachybotrylactam (**5**).

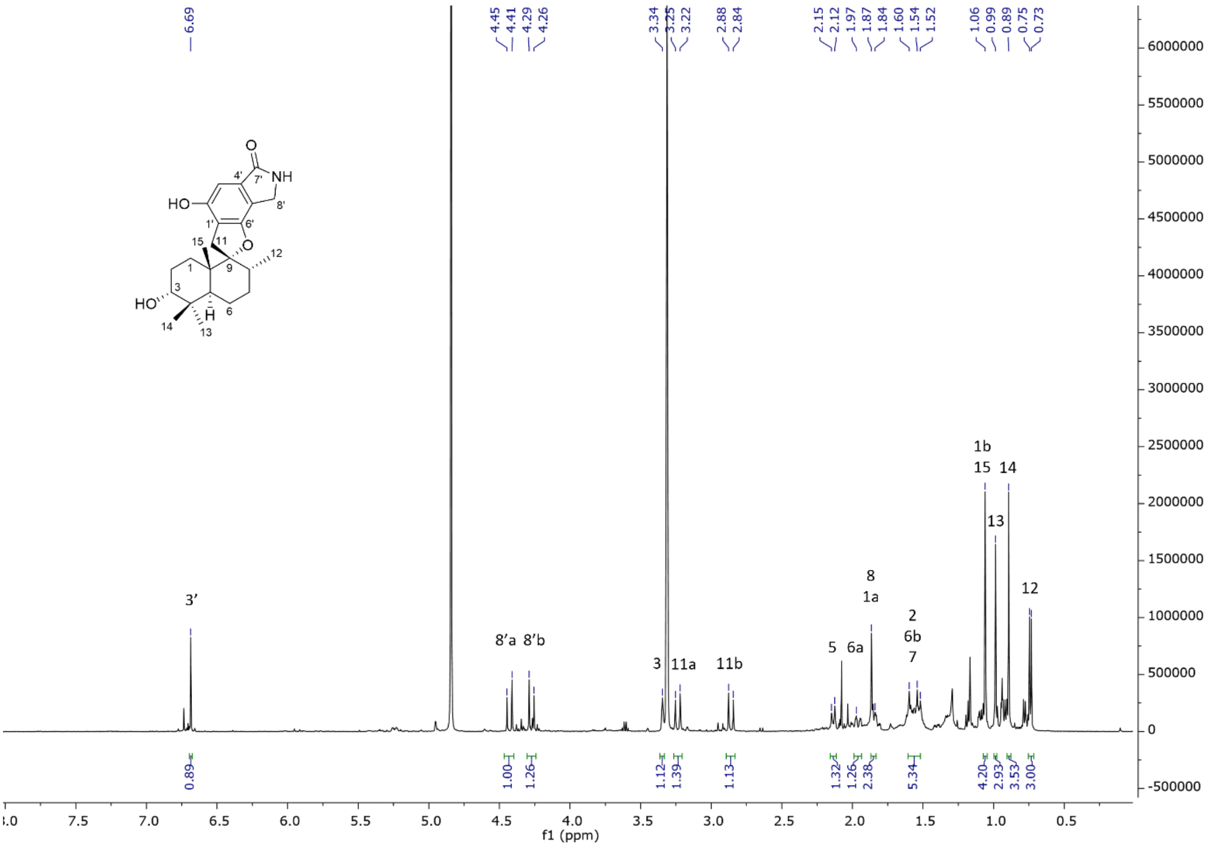

Figure S34. ^{1}H NMR spectrum (500 MHz, CD_3OD) of stachybotrylactam (**5**).

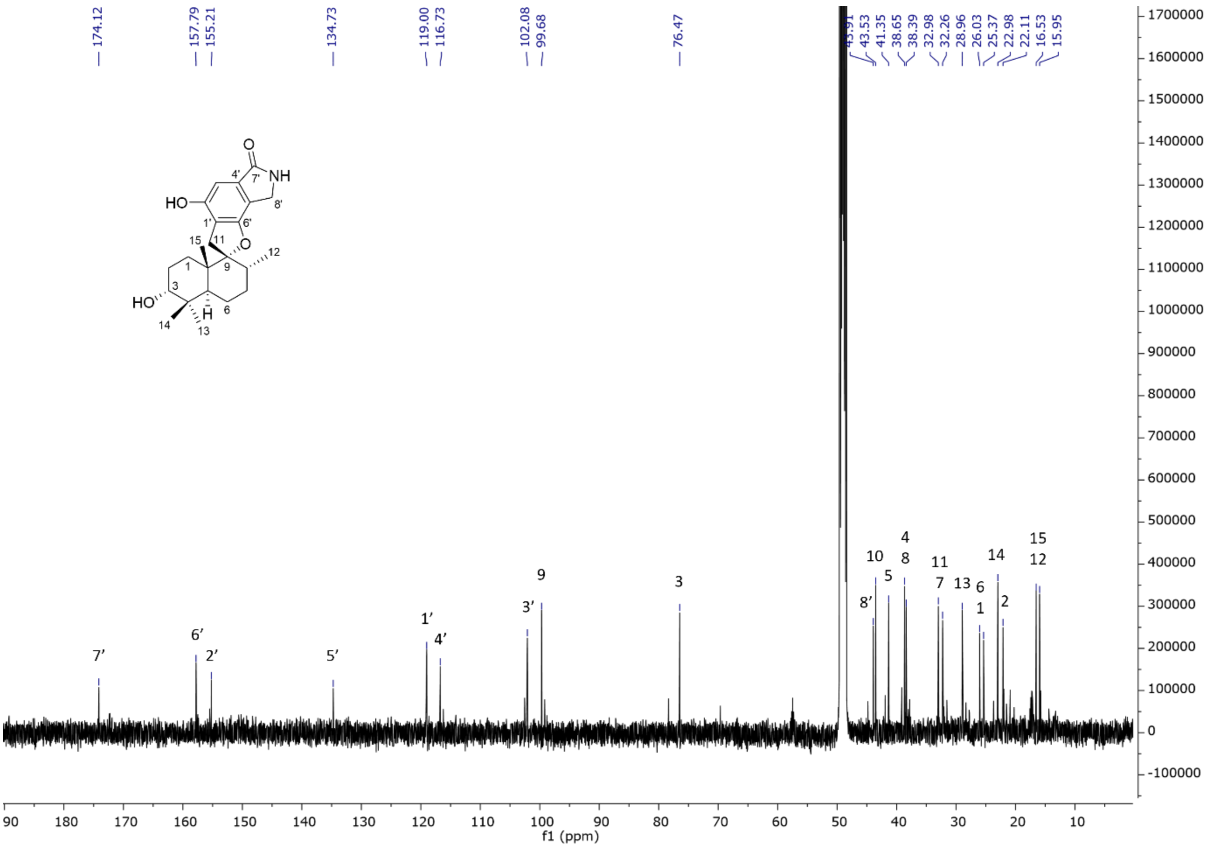

Figure S35. ^{13}C NMR spectrum (125 MHz, CD_3OD) of stachybotrylactam (**5**).

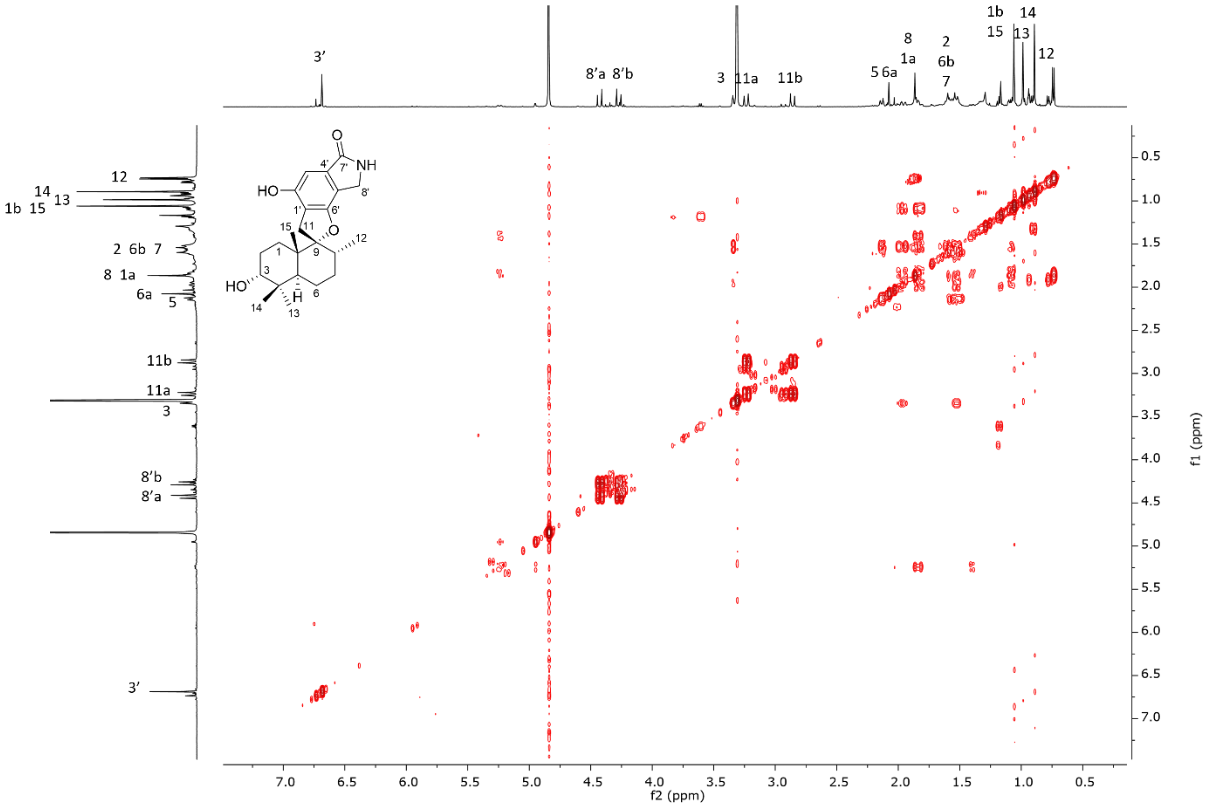

Figure S36. ¹H-¹H COSY NMR spectrum (500/500 MHz, CD_3OD) of stachybotrylactam (**5**).

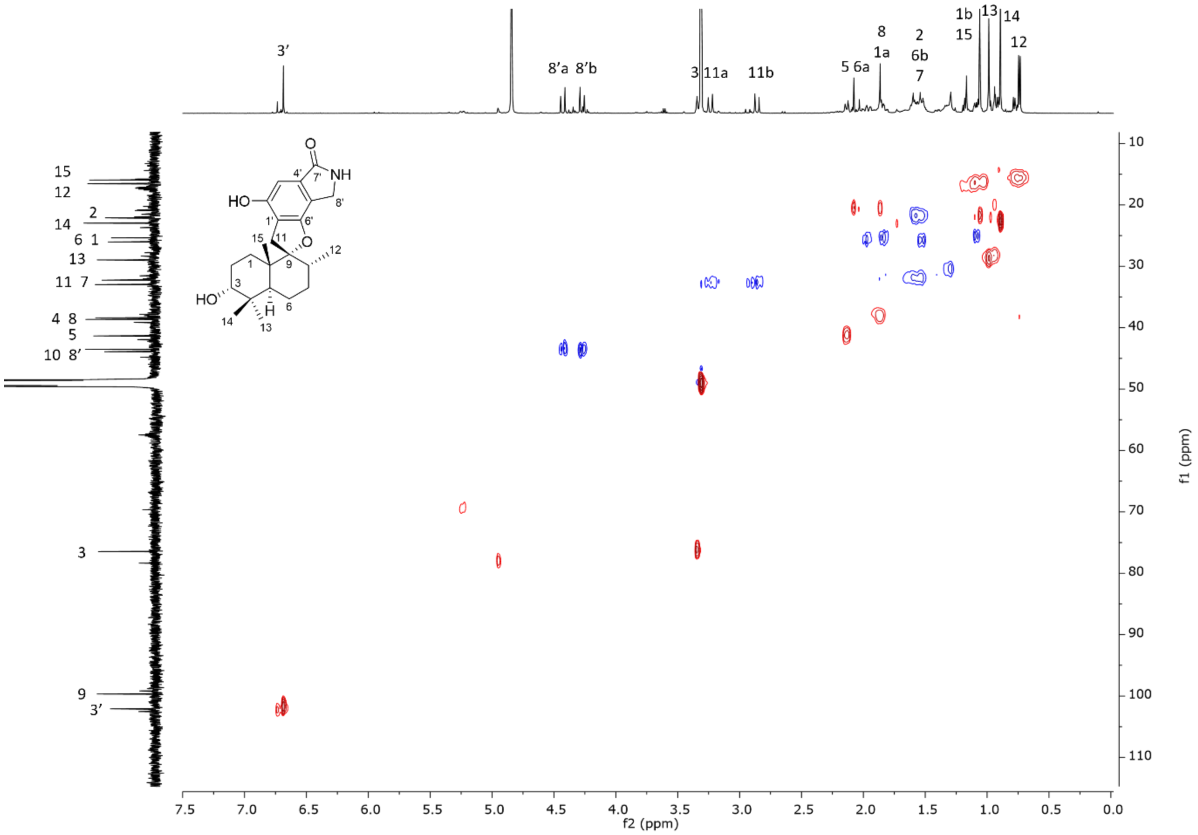

Figure S37. ¹H-¹³C HSQC NMR spectrum (500/125 MHz, CD_3OD) of stachybotrylactam (**5**).

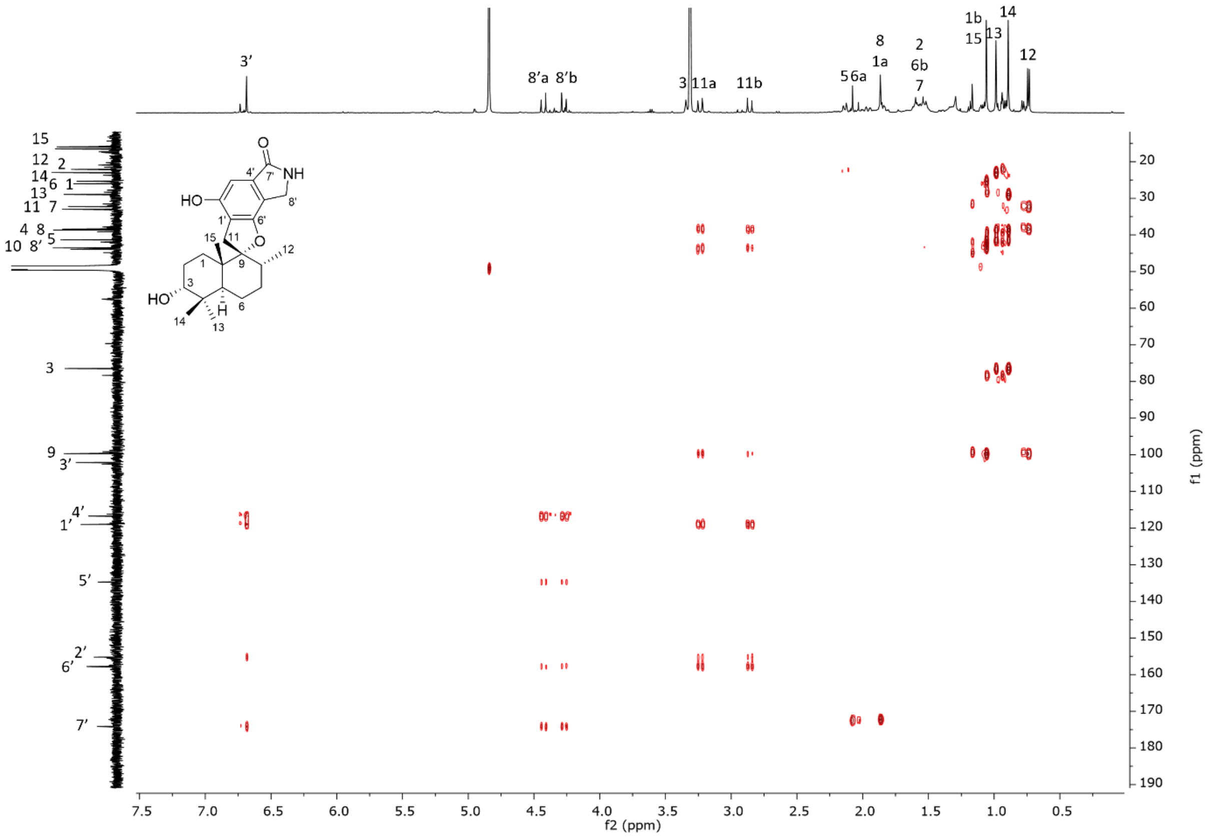

Figure S38. ^{1}H-^{13}C HMBC NMR spectrum (500/125 MHz, CD_3OD) of stachybotrylactam (**5**).

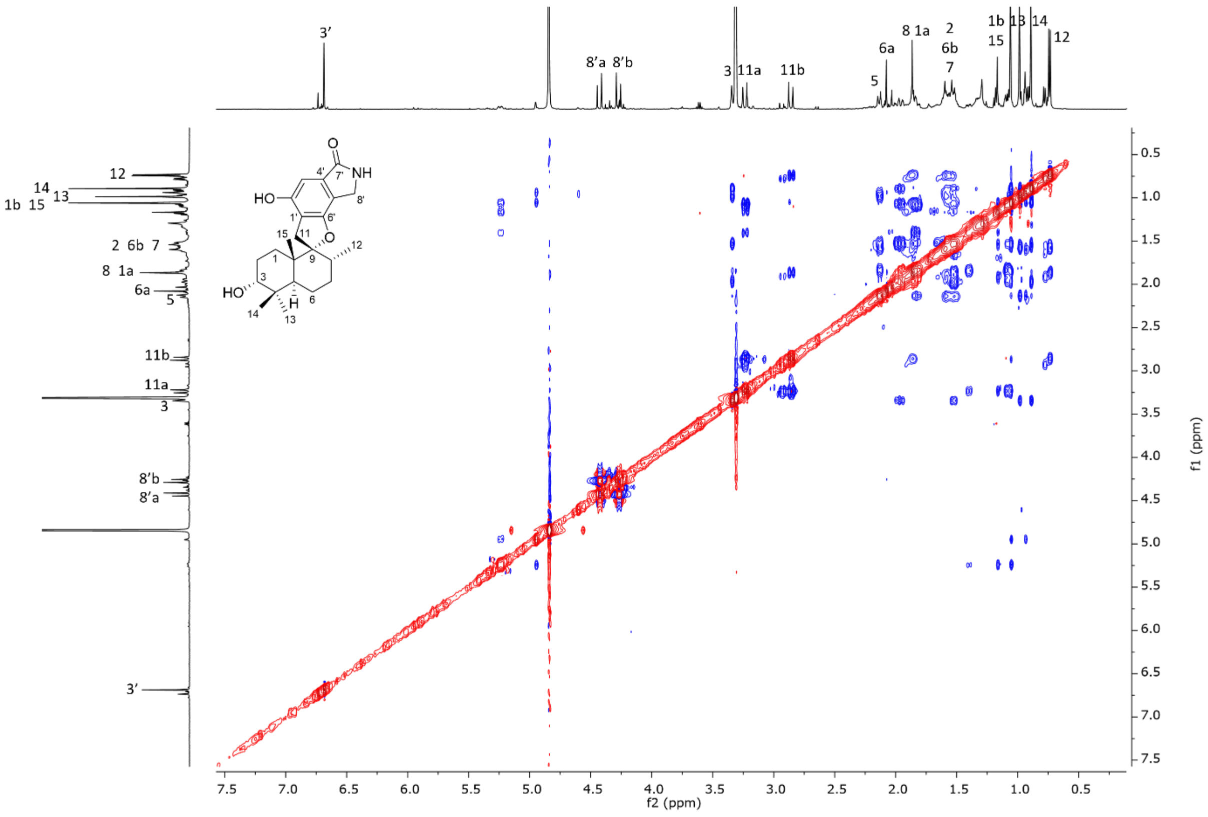

Figure S39. ^{1}H-^{1}H NOESY NMR spectrum (500/500 MHz, CD_3OD) of stachybotrylactam (**5**).

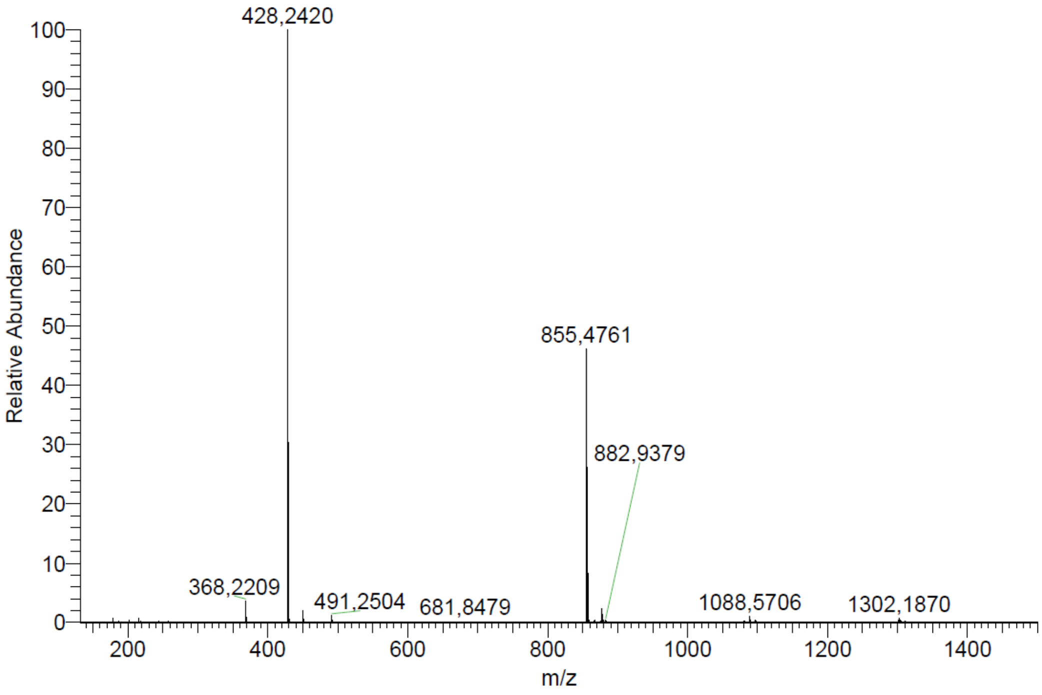

Figure S40. HRESI(+)MS spectrum of stachybotrylactam acetate (**6**).

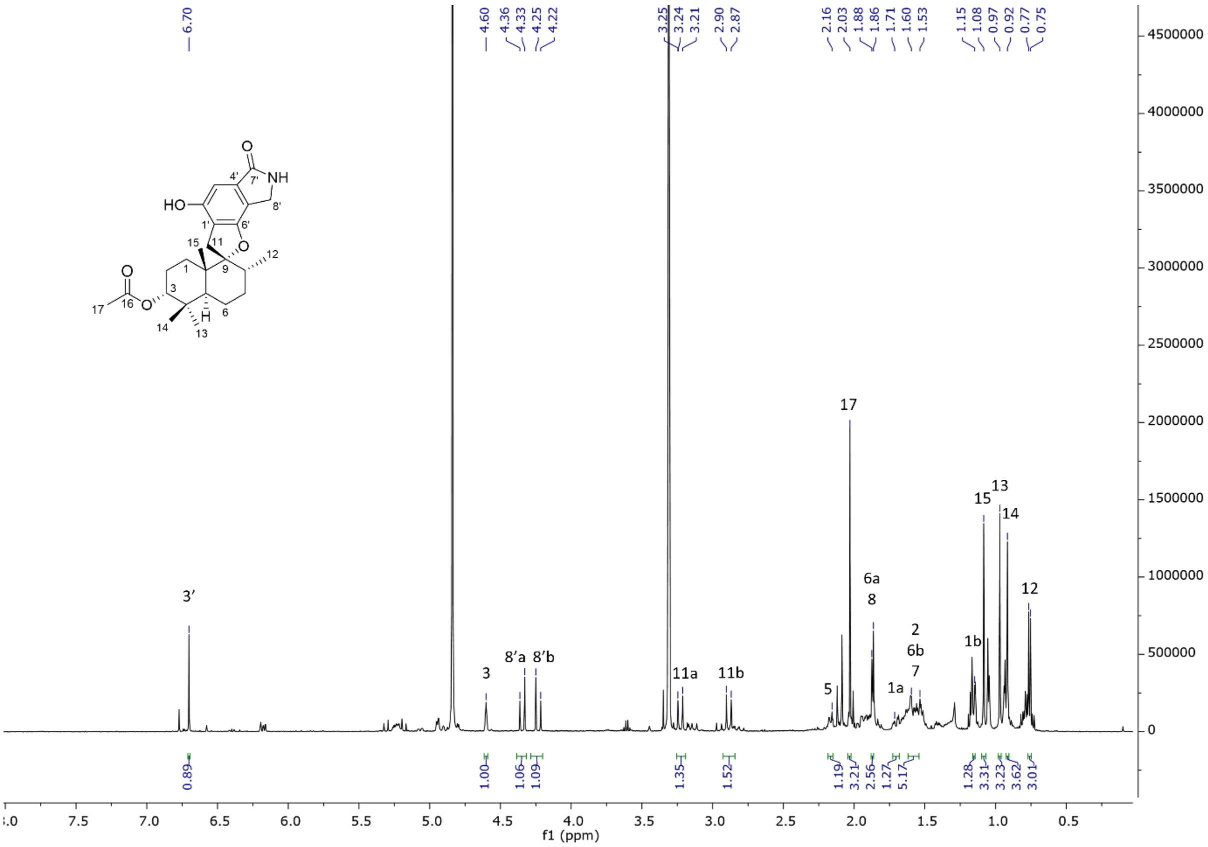

Figure S41. ^{1}H NMR spectrum (500 MHz, CD_3OD) of stachybotrylactam acetate (**6**).

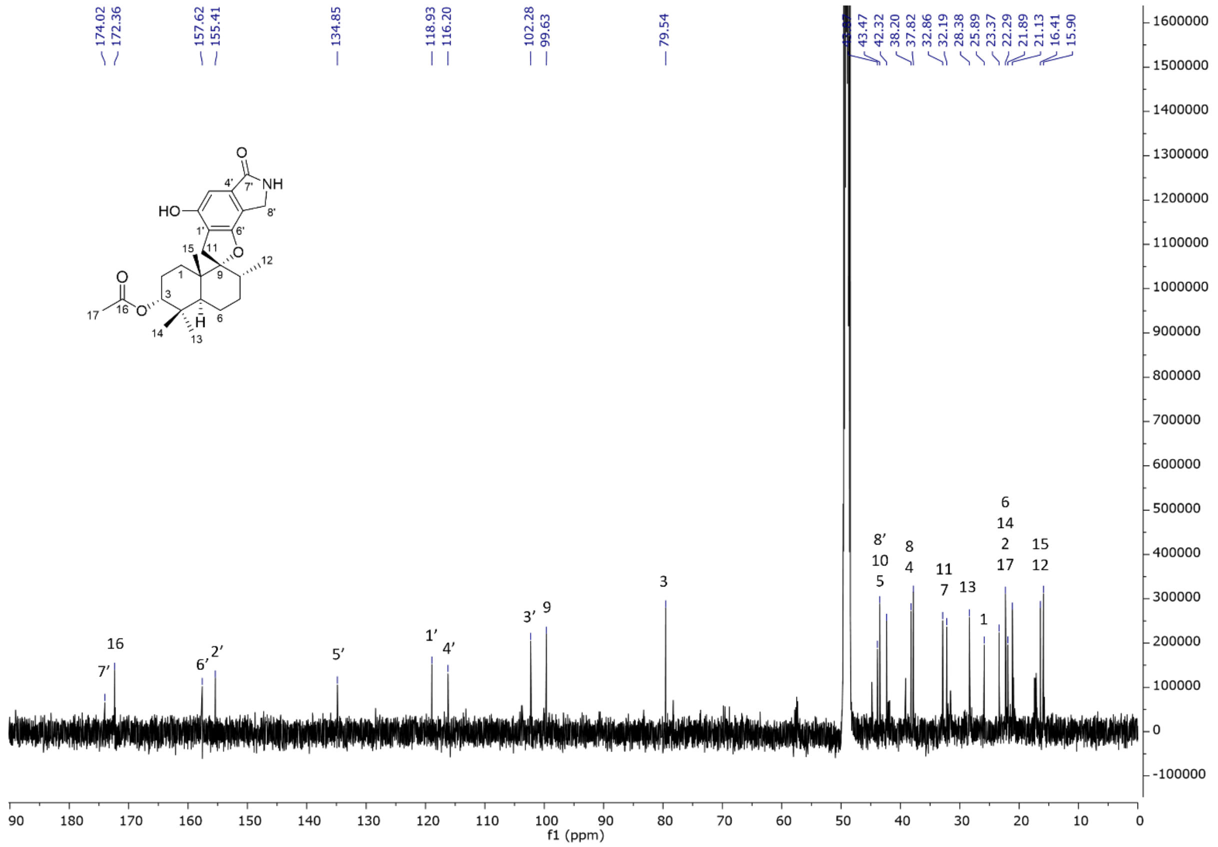

Figure S42. ^{13}C NMR spectrum (125 MHz, CD_3OD) of stachybotrylactam acetate (**6**).

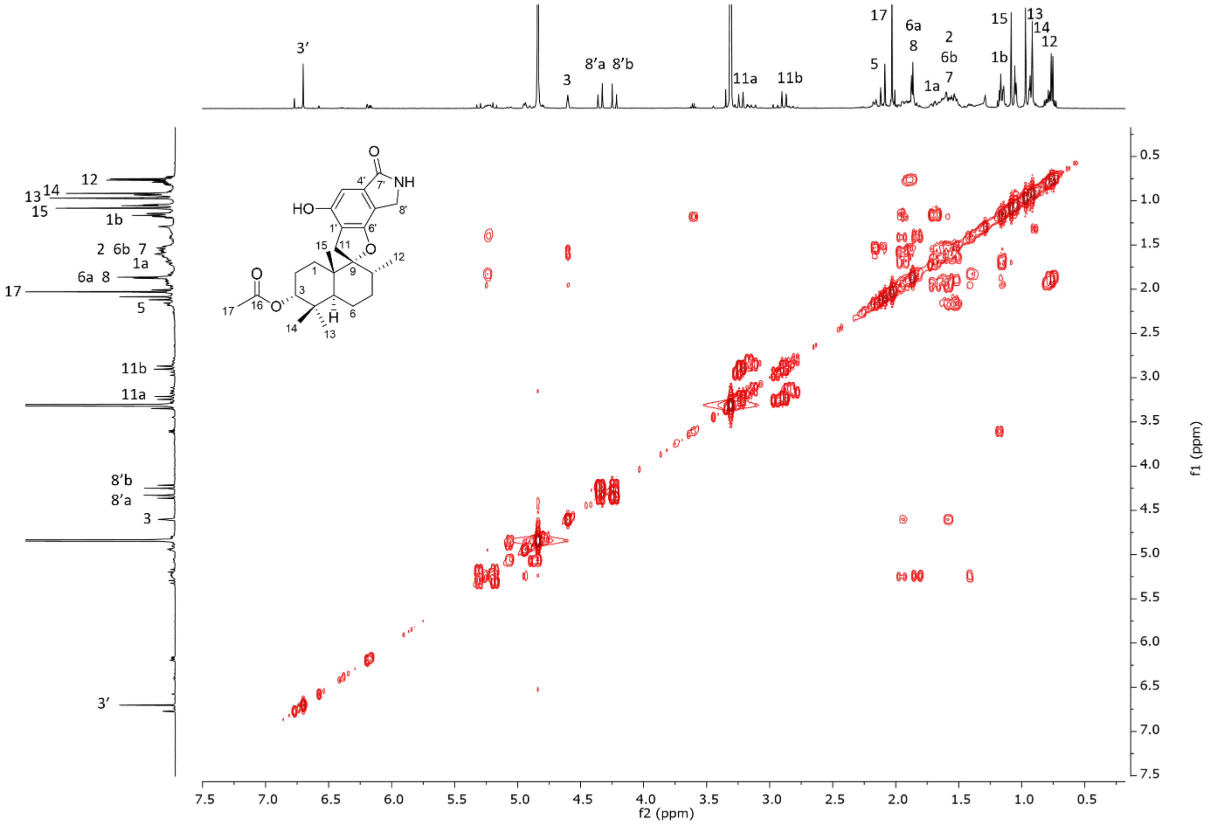

Figure S43. ^1H-^1H COSY NMR spectrum (500/500 MHz, CD_3OD) of stachybotrylactam acetate (**6**).

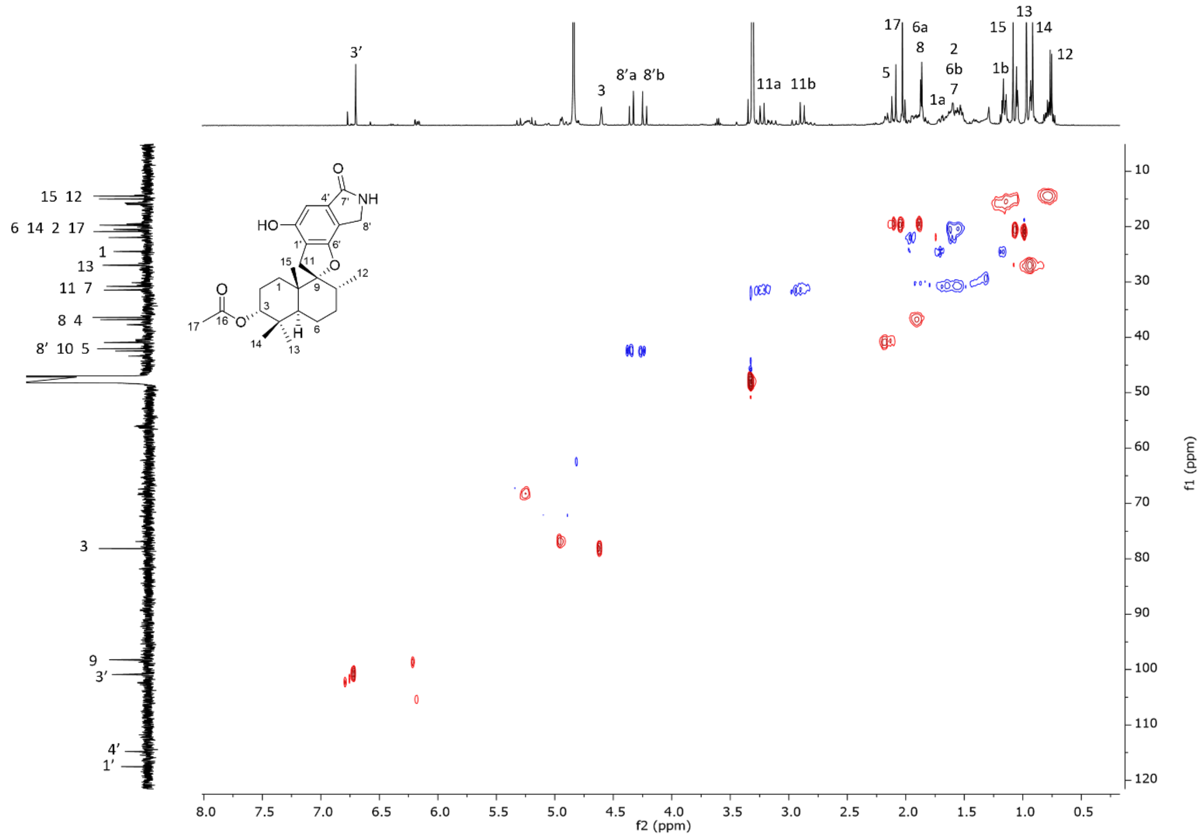

Figure S44. ^{1}H-^{13}C HSQC NMR spectrum (500/125 MHz, CD_3OD) of stachybotrylactam acetate (**6**).

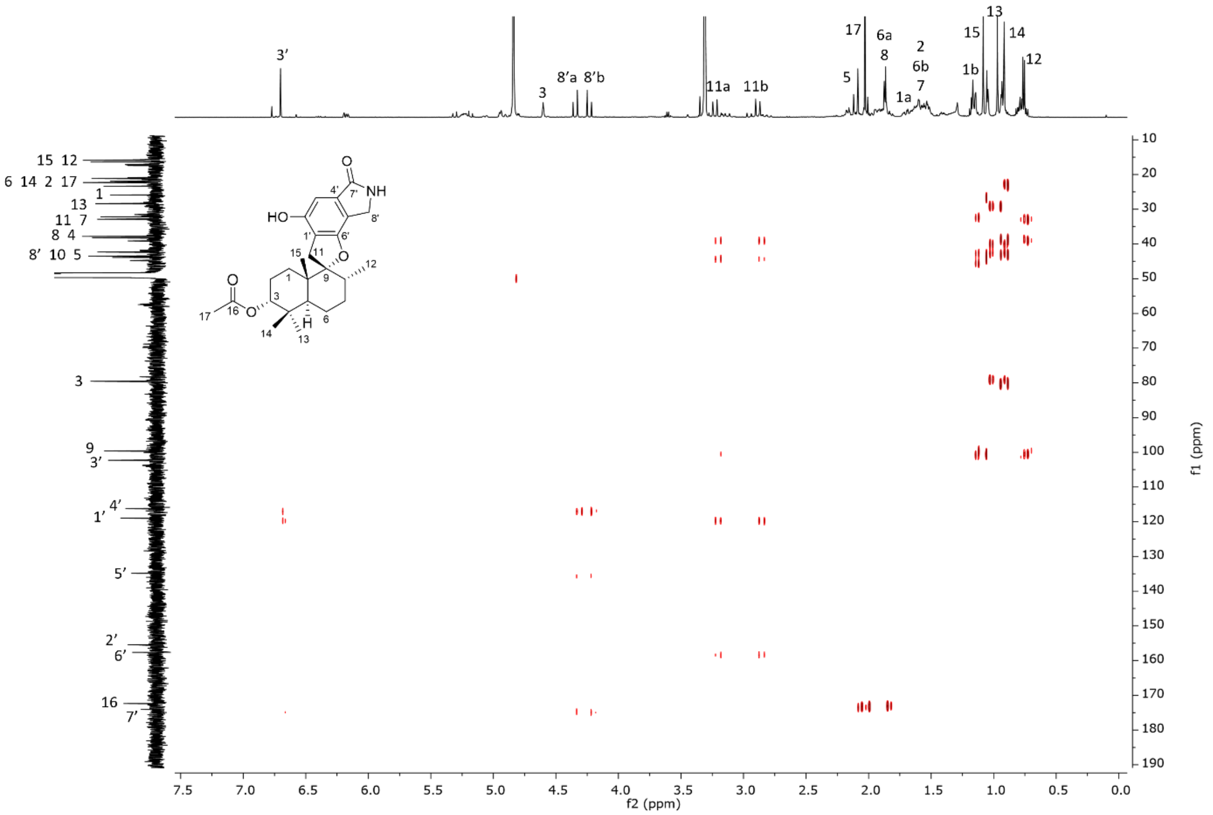

Figure S45. ^{1}H-^{13}C HMBC NMR spectrum (500/125 MHz, CD_3OD) of stachybotrylactam acetate (**6**).

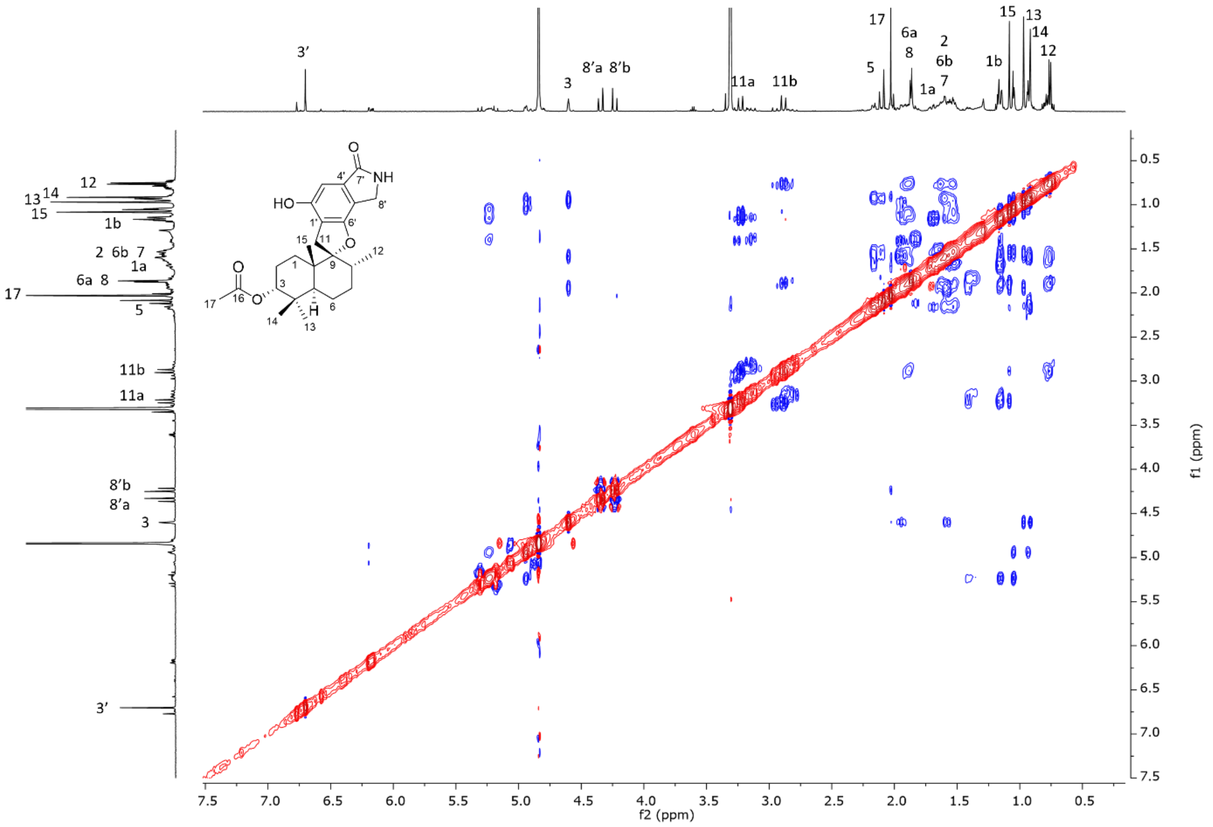

Figure S46. ^{1}H-^{1}H NOESY NMR spectrum (500/500 MHz, CD_3OD) of stachybotrylactam acetate (**6**).

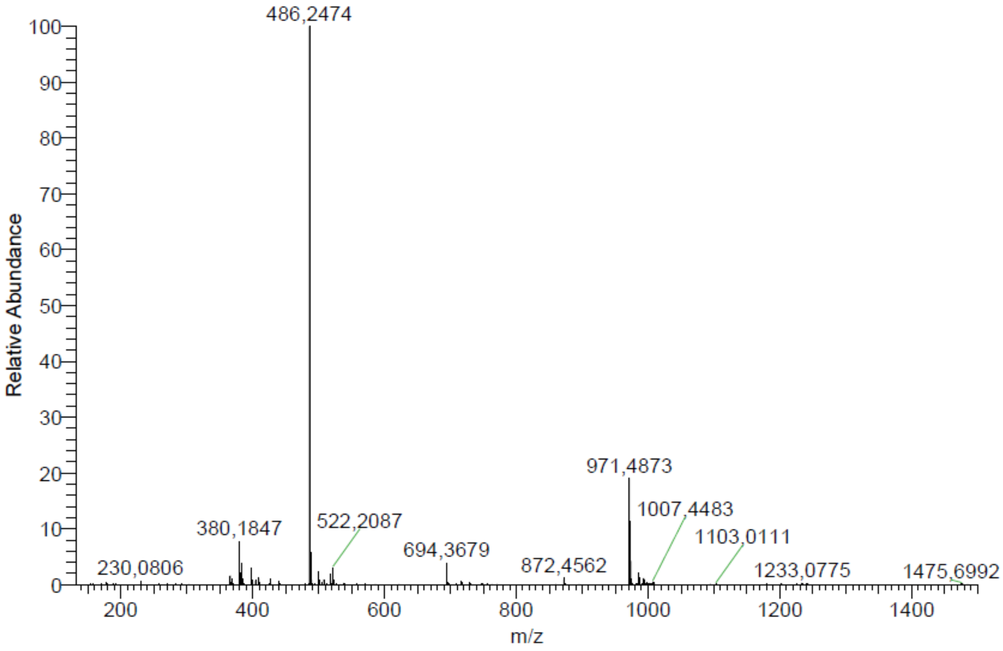

Figure S47. HRESI(+)MS spectrum of 2α-acetoxystachybotrylactam acetate (**7**).

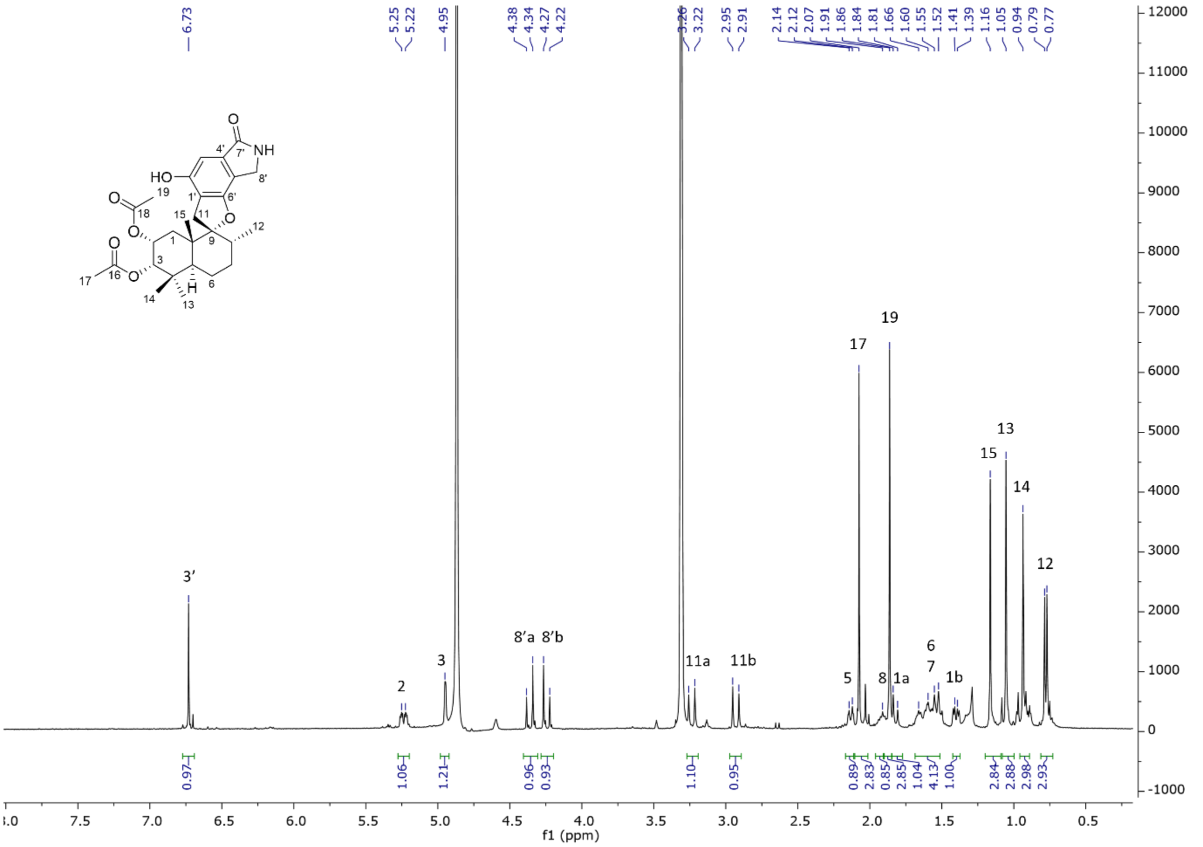

Figure S48. ^{1}H NMR spectrum (500 MHz, CD_3OD) of 2α-acetoxystachybotrylactam acetate (**7**).

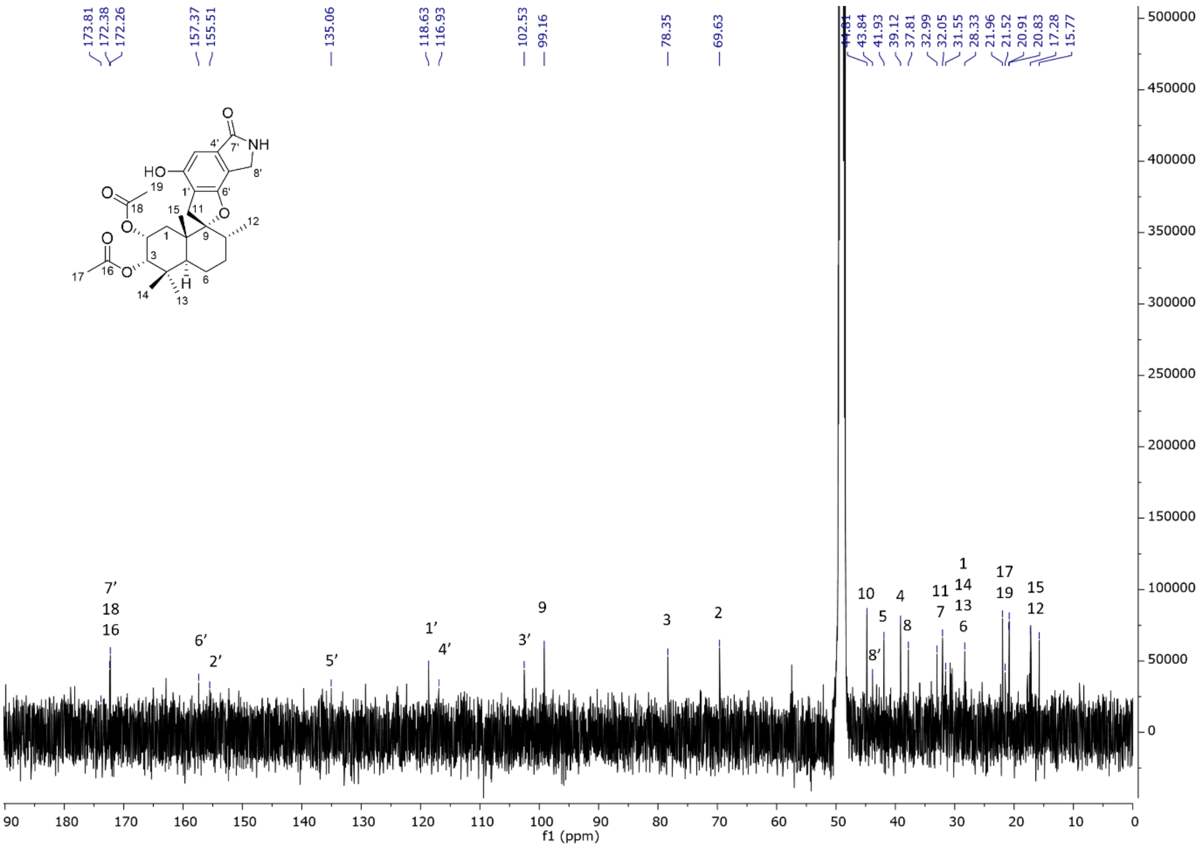

Figure S49. ^{13}C NMR spectrum (125 MHz, CD_3OD) of 2α-acetoxystachybotrylactam acetate (**7**).

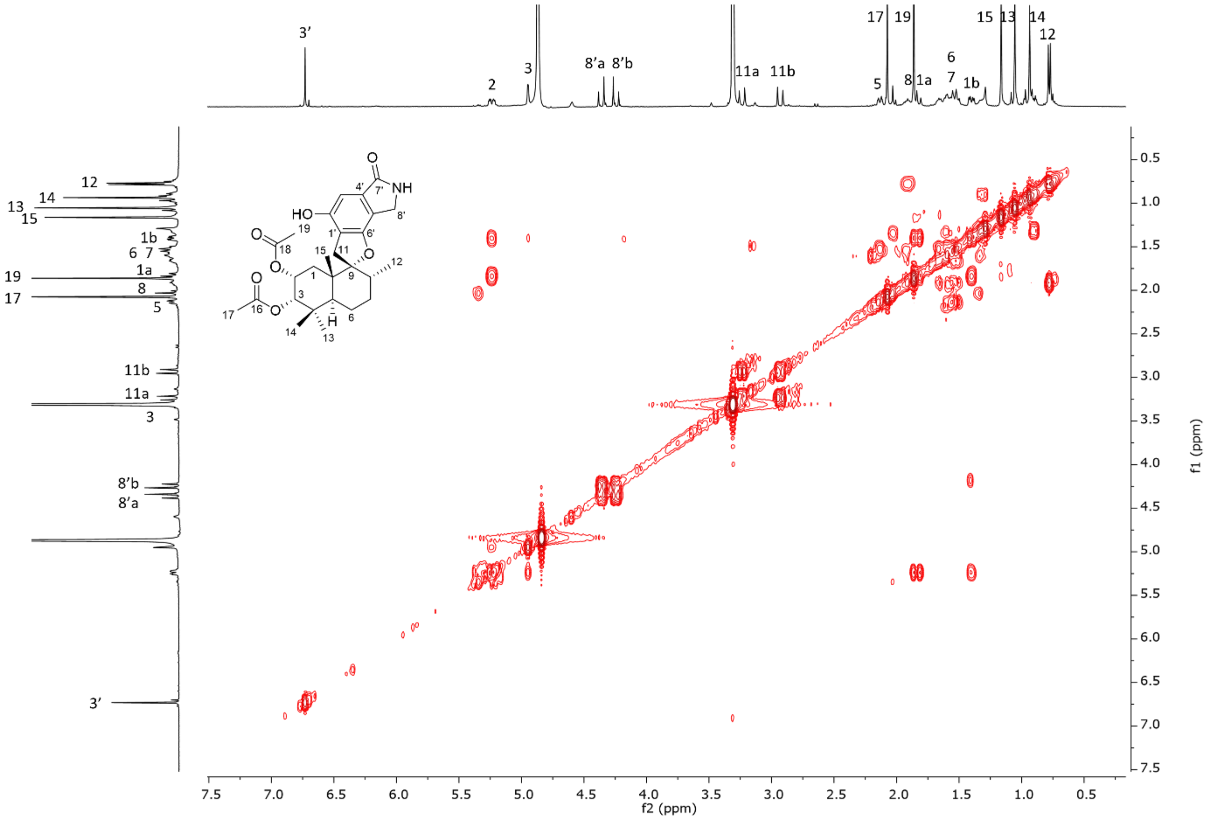

Figure S50. ^{1}H-^{1}H COSY NMR spectrum (500/500 MHz, CD_3OD) of 2*α*-acetoxystachybotrylactam acetate (**7**).

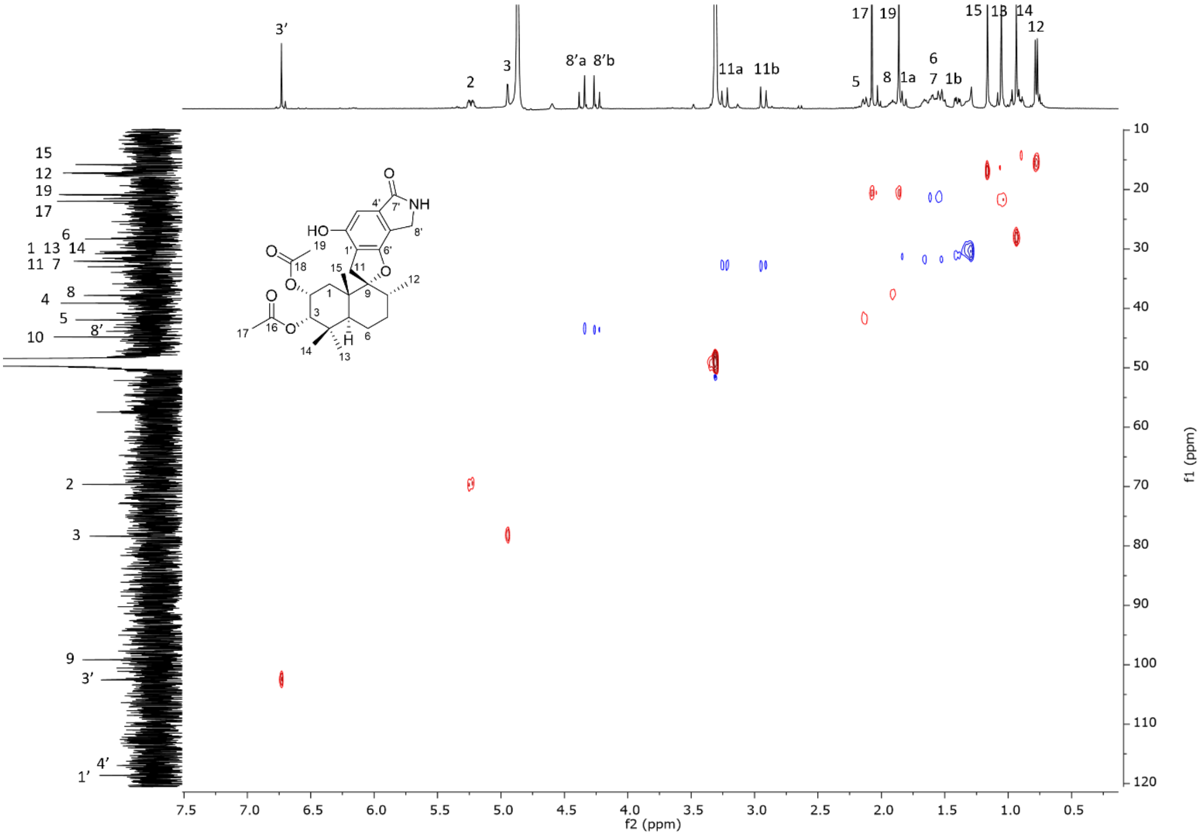

Figure S51. ^{1}H-^{13}C HSQC NMR spectrum (500/125 MHz, CD_3OD) of 2*α*-acetoxystachybotrylactam acetate (**7**).

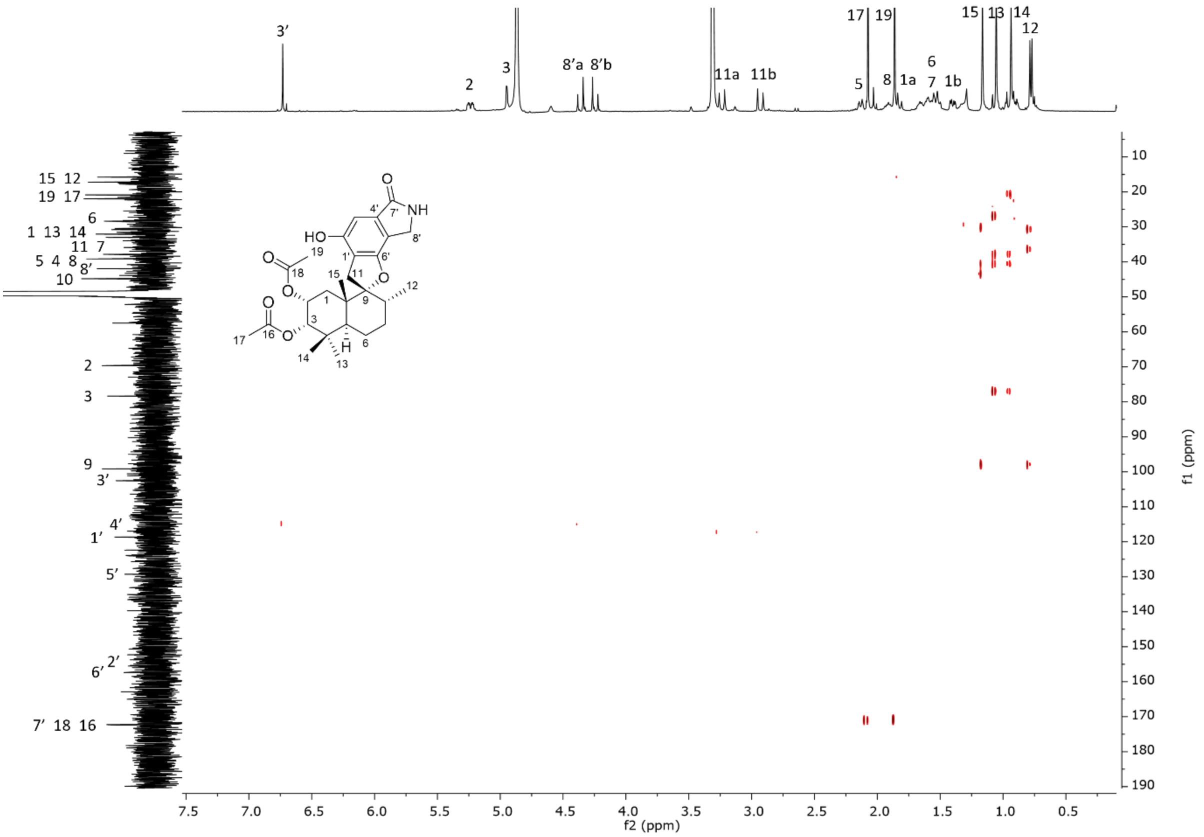

Figure S52. ^{1}H-^{13}C HMBC NMR spectrum (500/125 MHz, CD_3OD) of 2*α*-acetoxystachybotrylactam acetate (**7**).

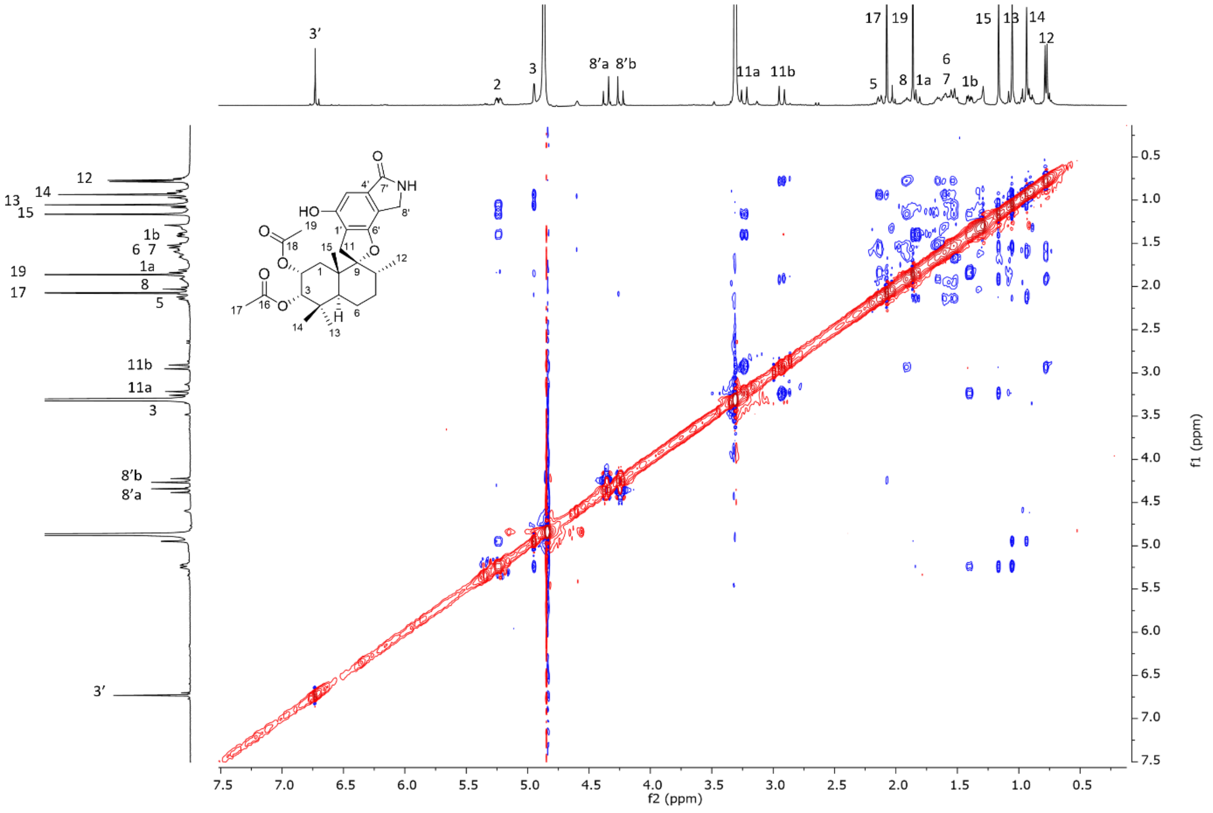

Figure S53. ^{1}H-^{1}H NOESY NMR spectrum (500/500 MHz, CD_3OD) of 2*α*-acetoxystachybotrylactam acetate (**7**).

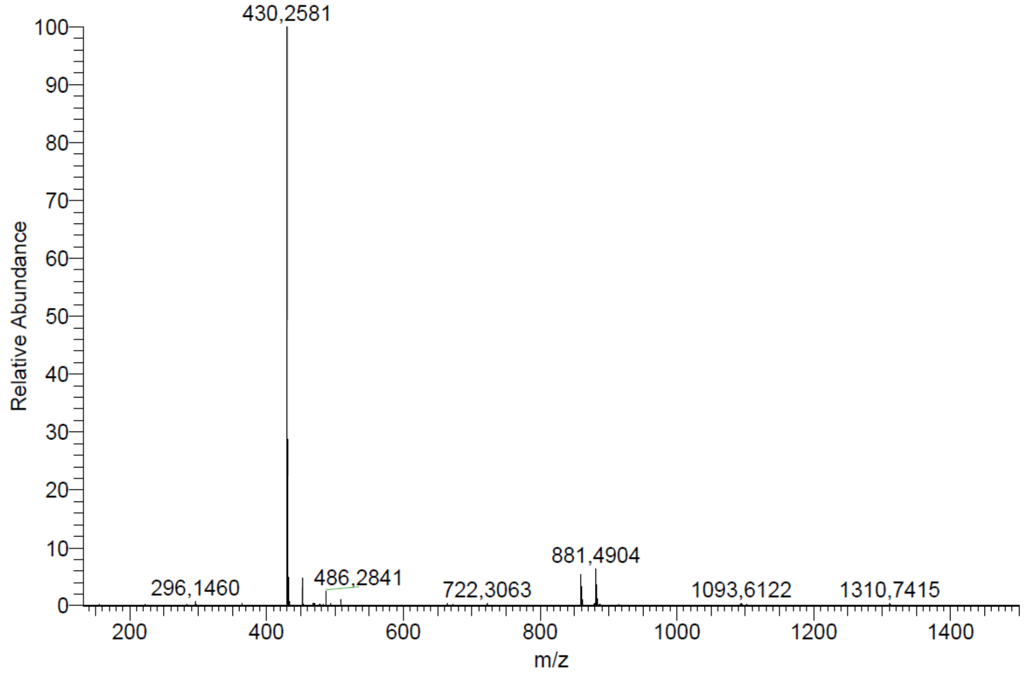

Figure S54. HRESI(+)MS spectrum of stachybotramide (**8**).

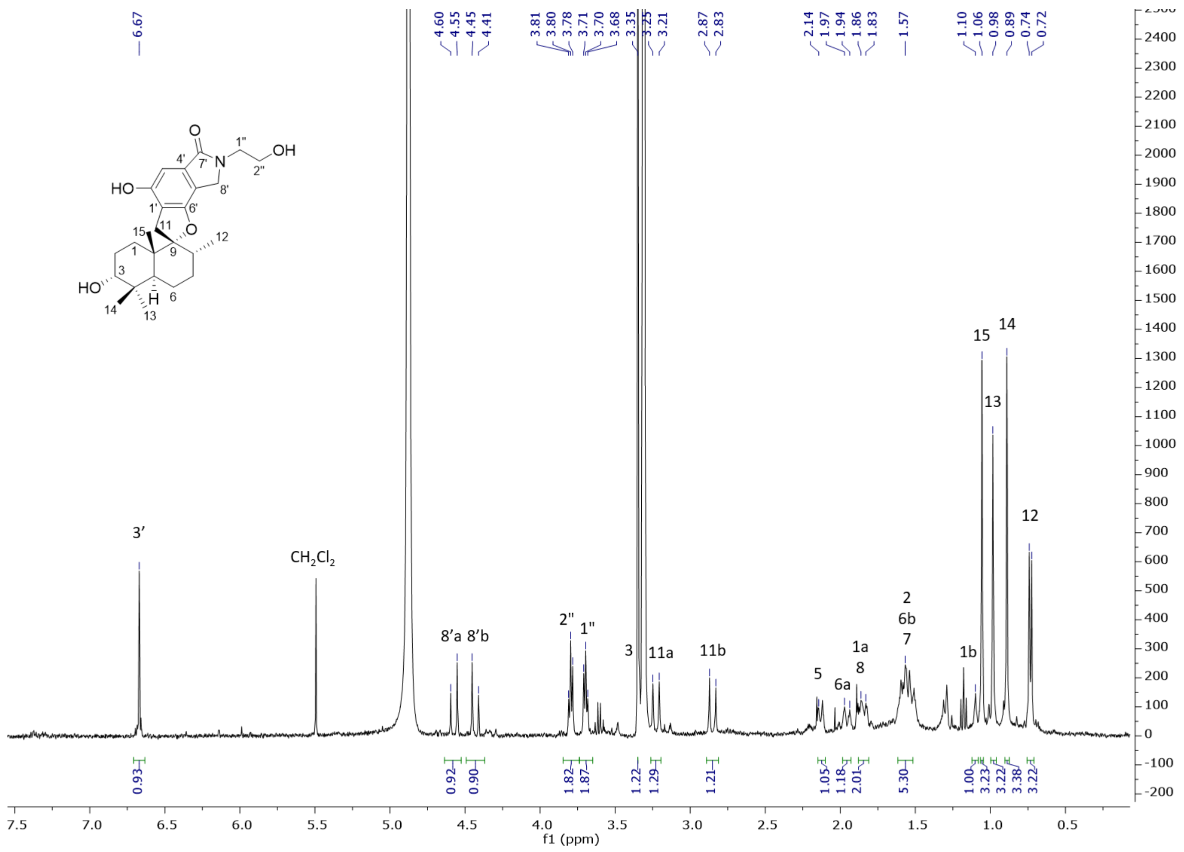

Figure S55. ^{1}H NMR spectrum (500 MHz, CD_3OD) of stachybotramide (**8**).

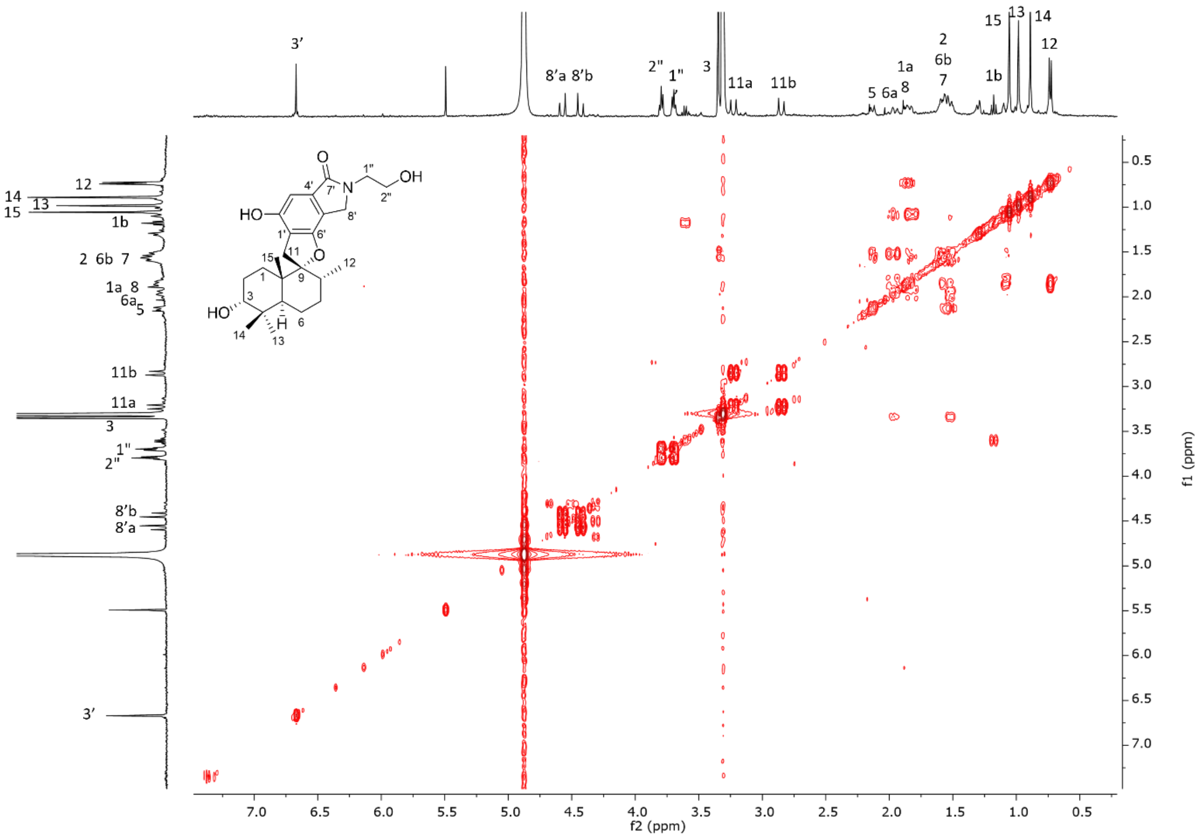

Figure S56. ^1H-^1H COSY NMR spectrum (500/500 MHz, CD_3OD) of stachybotramide (**8**).

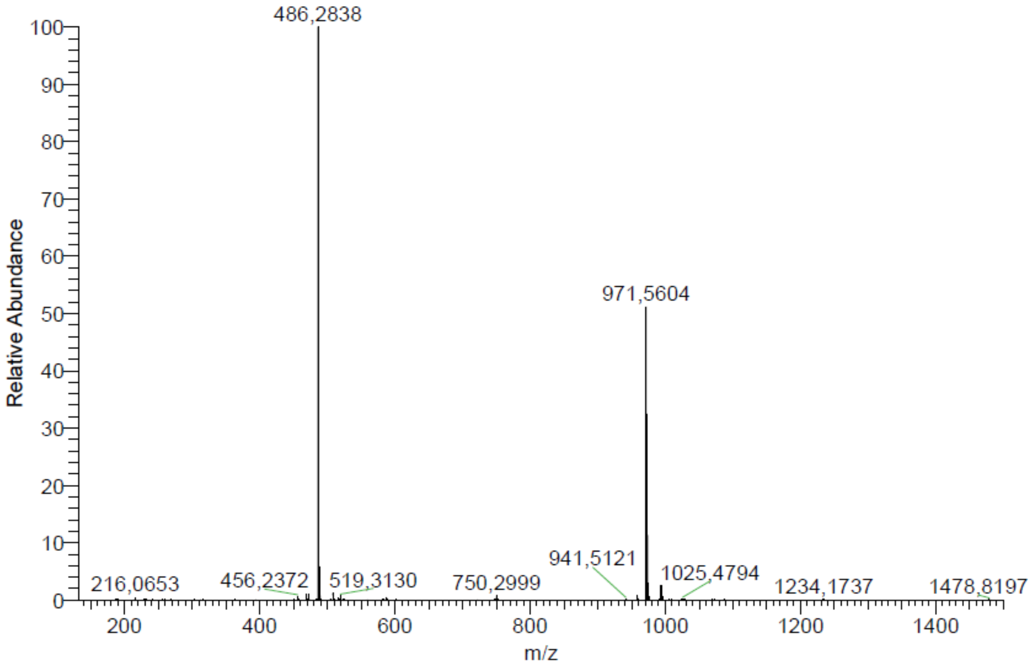

Figure S57. HRESI(+)MS spectrum of chartarlactam B (**9**).

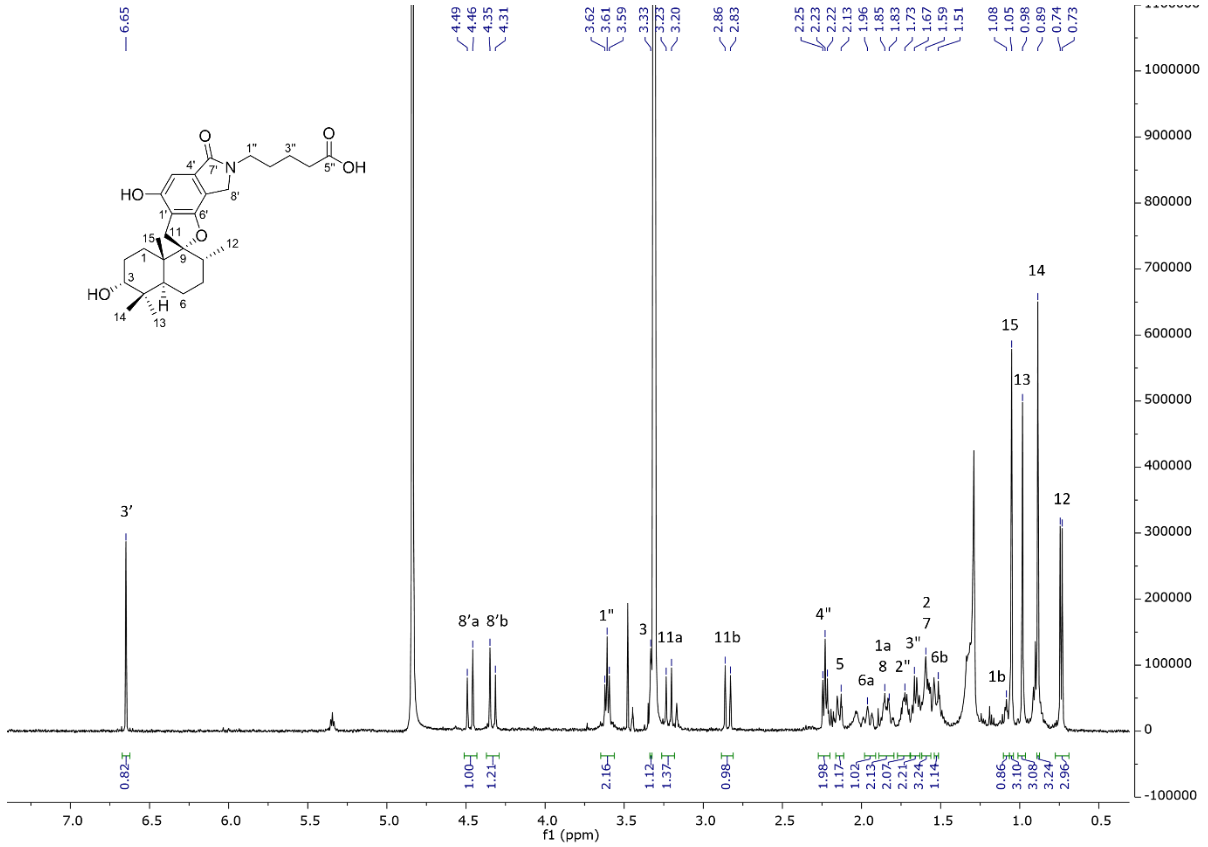

Figure S58. ^{1}H NMR spectrum (500 MHz, CD_3OD) of chartarlactam B (**9**).

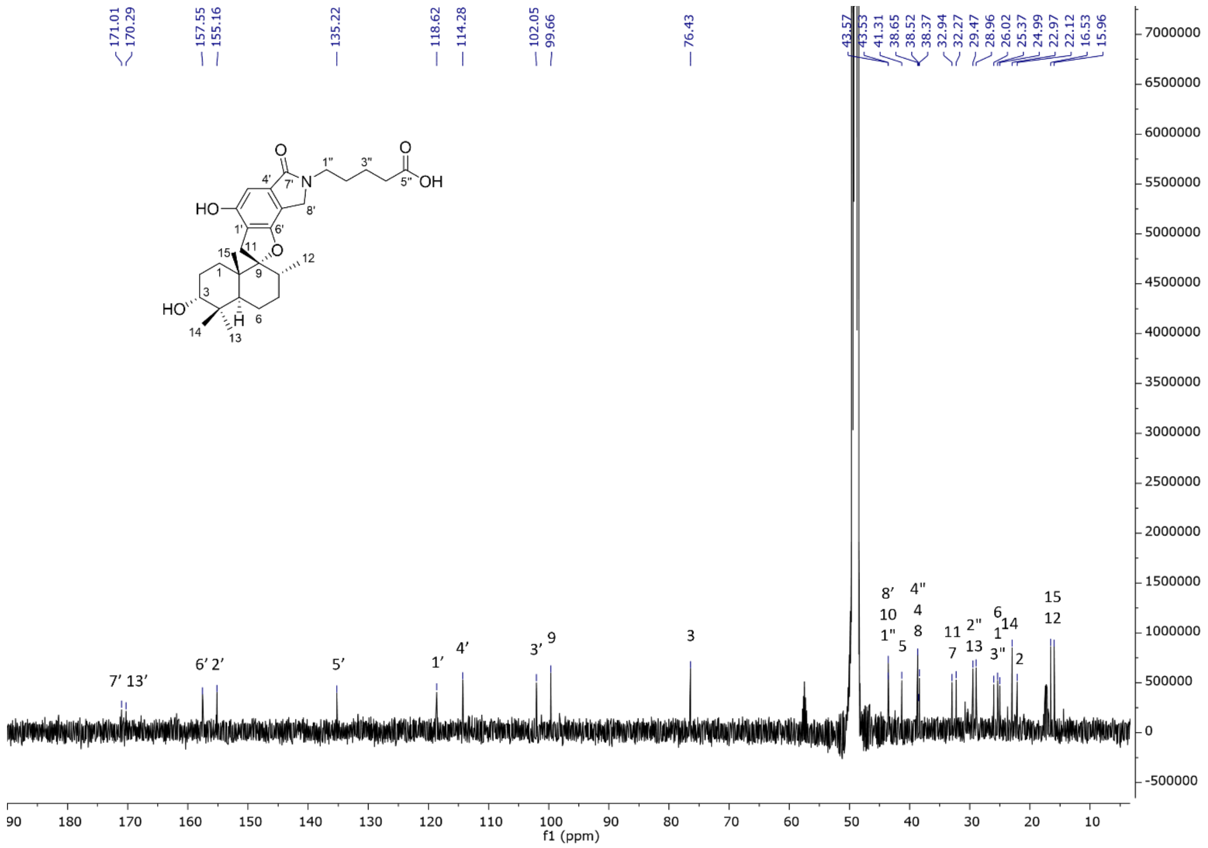

Figure S59. ^{13}C NMR spectrum (125 MHz, CD_3OD) of chartarlactam B (**9**).

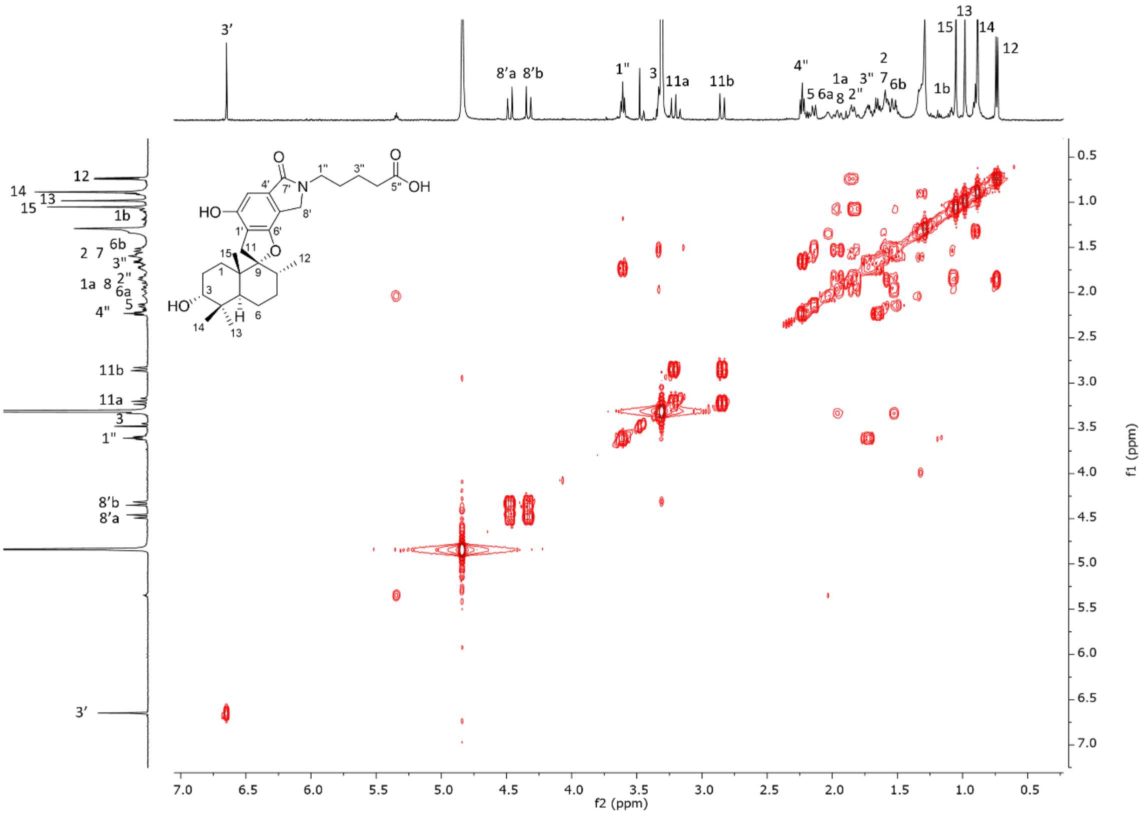

Figure S60. ^{1}H-^{1}H COSY NMR spectrum (500/500 MHz, CD_3OD) of chartarlactam B (**9**).

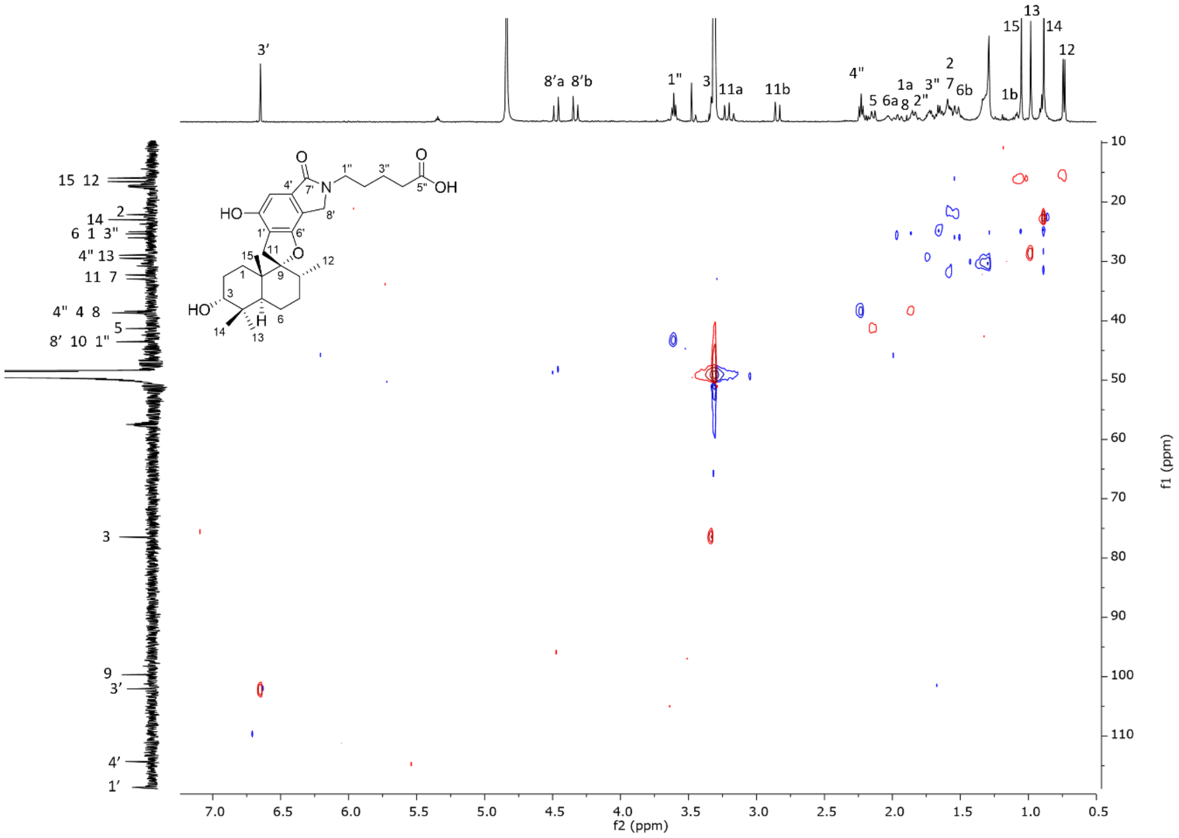

Figure S61. ^{1}H-^{13}C HSQC NMR spectrum (500/125 MHz, CD_3OD) of chartarlactam B (**9**).

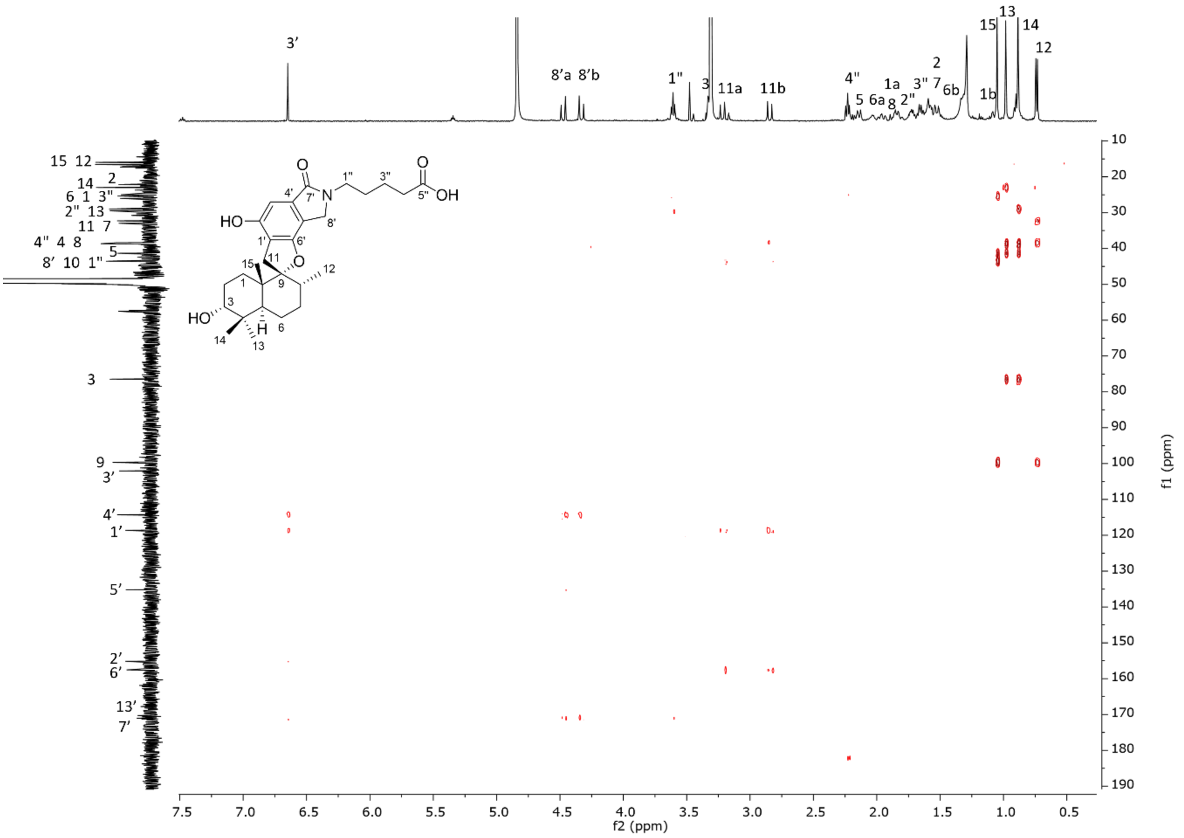

Figure S62. ^{1}H-^{13}C HMBC NMR spectrum (500/125 MHz, CD_3OD) of chartarlactam B (**9**).

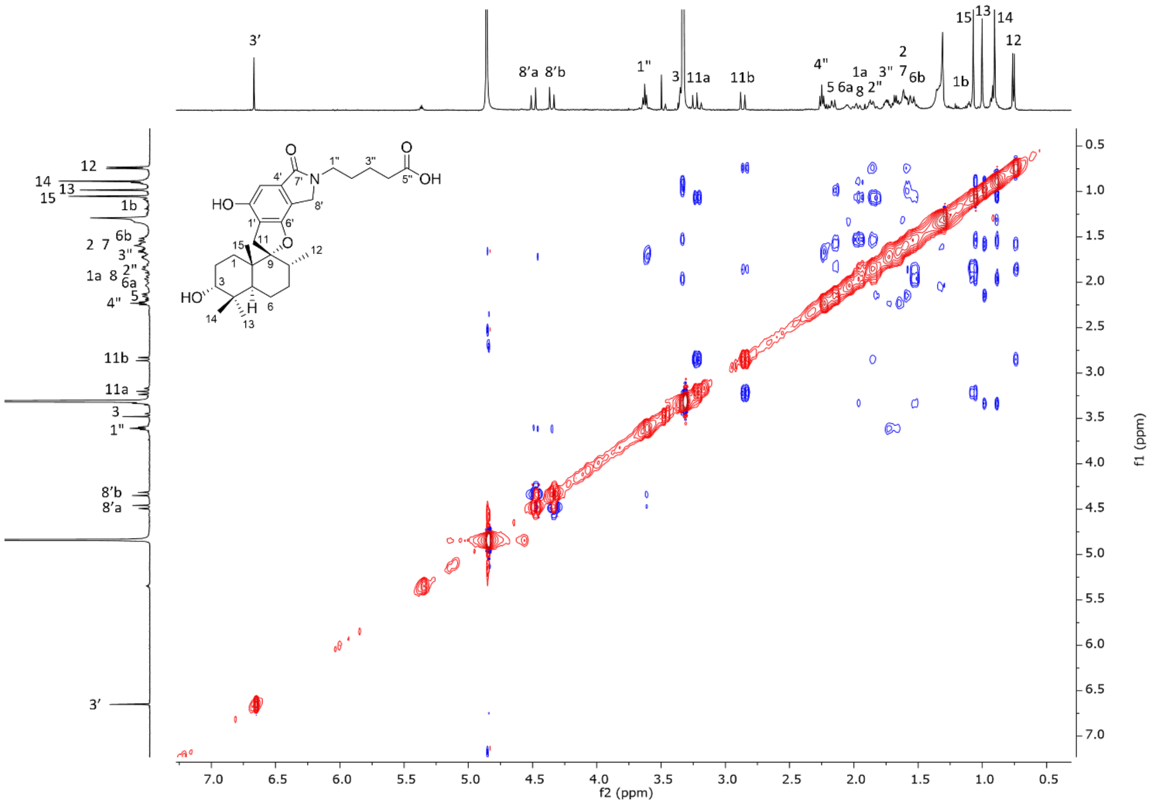

Figure S63. ^{1}H-^{1}H NOESY NMR spectrum (500/500 MHz, CD_3OD) of chartarlactam B (**9**).

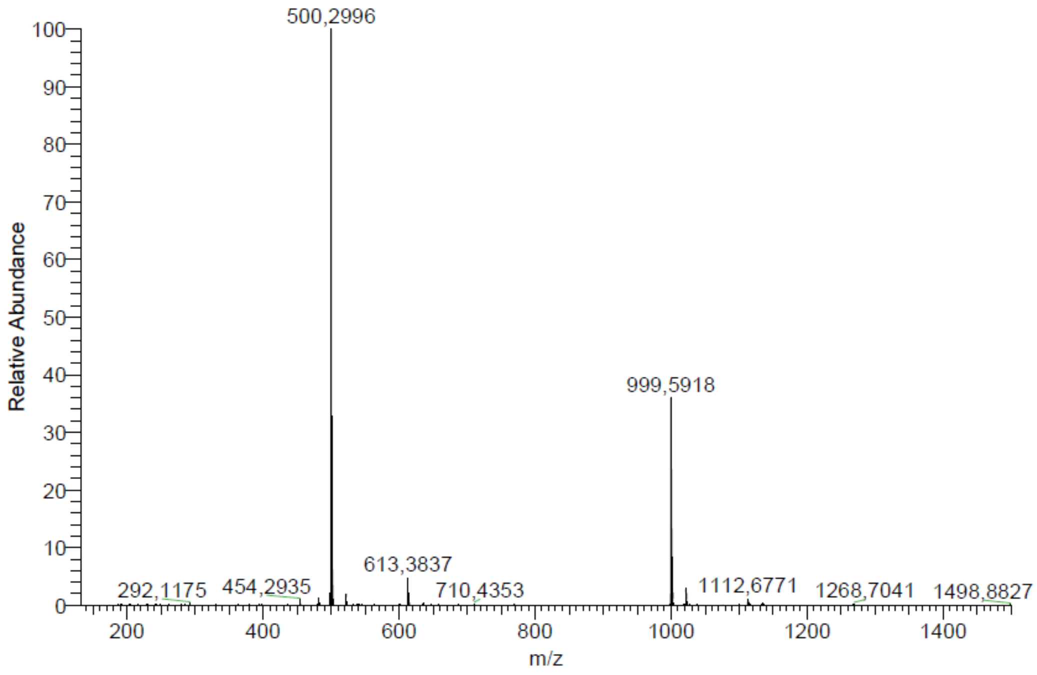

Figure S64. HRESI(+)MS spectrum of F1839-J (**10**).

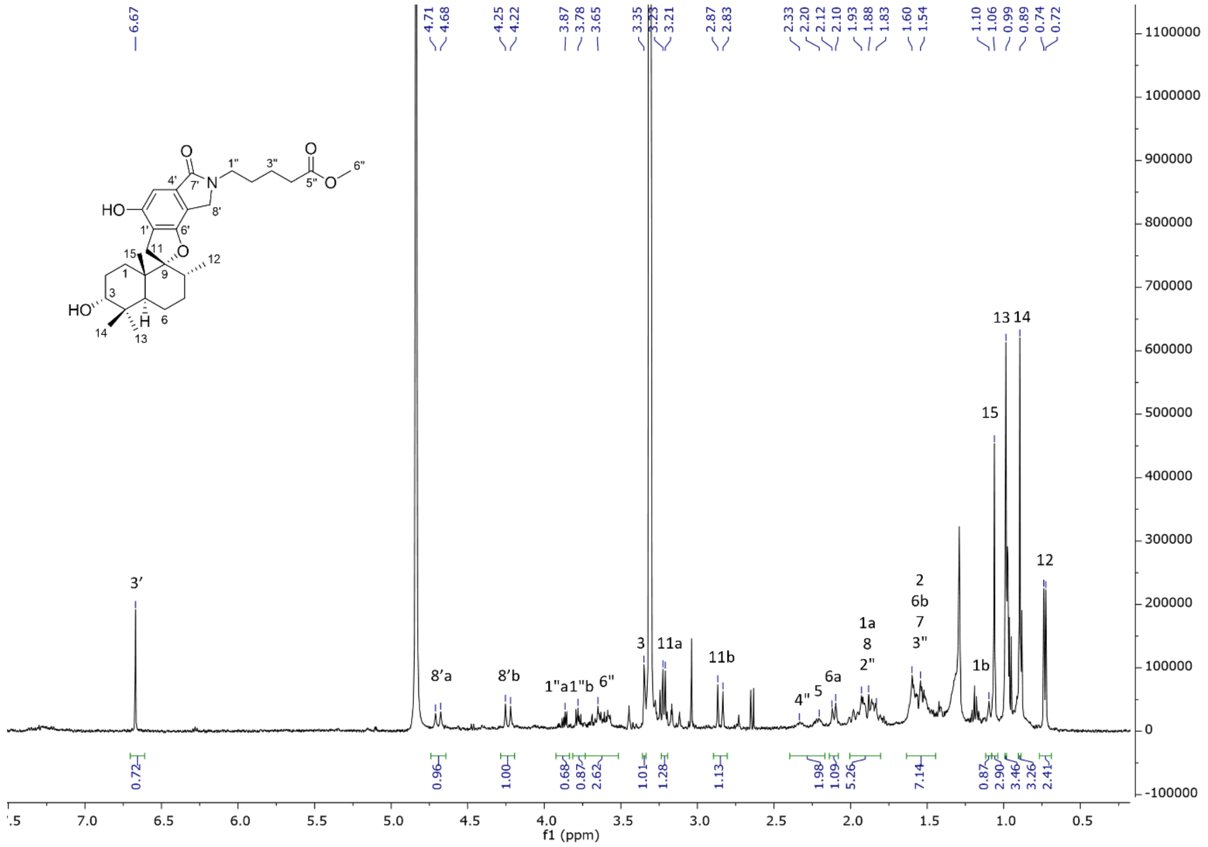

Figure S65. ^{1}H NMR spectrum (500 MHz, CD_3OD) of F1839-J (**10**).

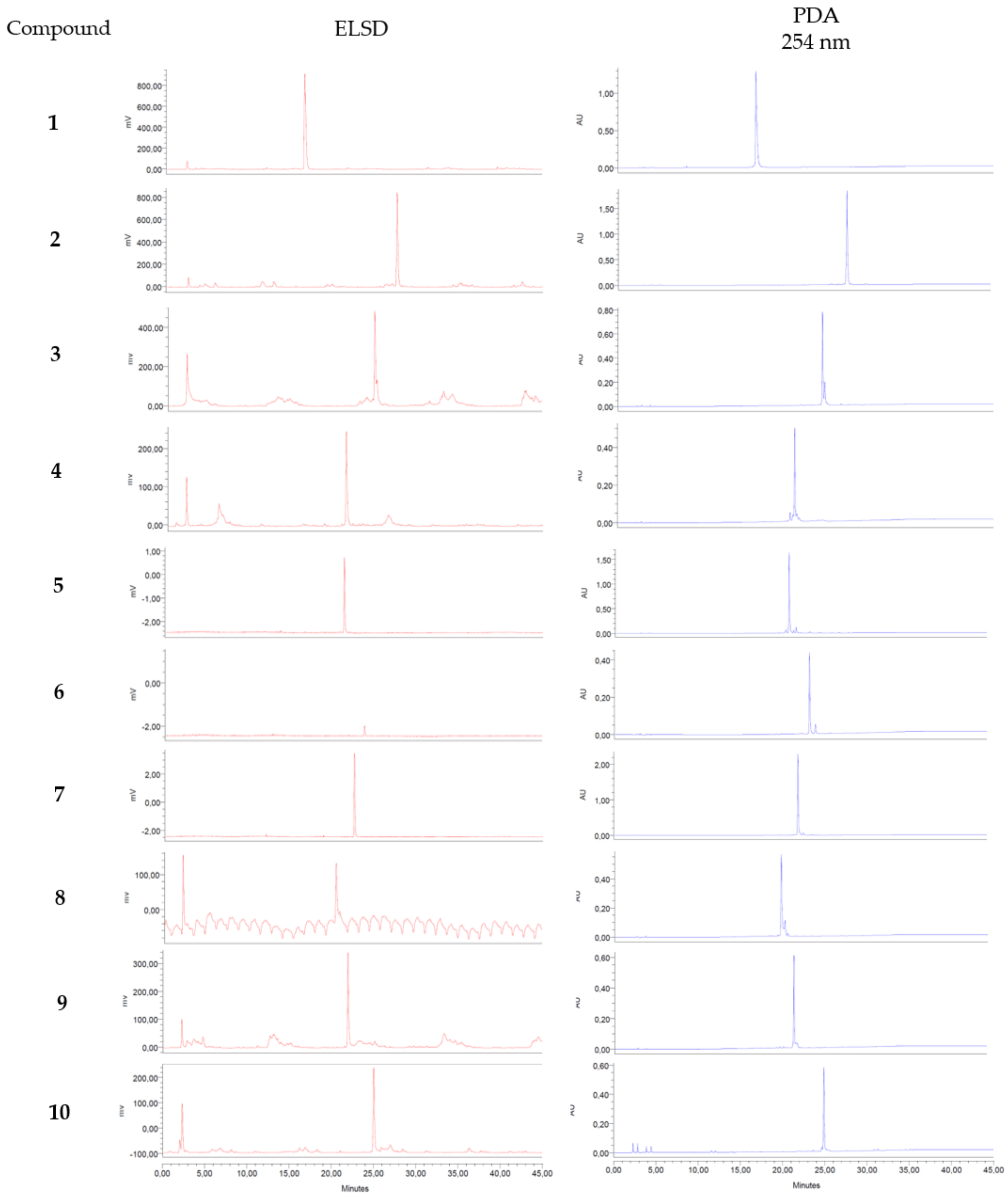

Figure S66. HPLC-PDA-ELSD chromatograms of compounds **1** – **10**.

2. NMR tables

Table S1. ^{1}H and ^{13}C NMR data of **1** (500/125 MHz, CD_3OD) and synthetic stachybotry-arginine (600/150 MHz, (CD_3OD)[32].

	**1**		**Stachybotry-arginine**	
N°	δ_C (ppm)/mult.	δ_H (ppm)/mult./*J*(Hz)	δ_C (ppm)/mult.	δ_H (ppm)/mult./*J*(Hz)
1*α*	25.3, CH_2	1.09, m	25.3, CH_2	1.07, m
1*β*		1.90, m		
2	22.1, CH_2	1.56, m	22.1, CH_2	1.52, m
3	76.3, CH	3.33, s	76.3, CH	3.33, s
4	38.5, C	-	38.4, C	-
5	41.3, CH	2.15, m	41.3, CH	2.13, m
6*α*	26.1, CH	1.90, m	26.1, CH	1.55, m
6*β*		1.56, m		
7	32.3, CH_2	1.56, m	32.3, CH_2	1.59, m
8	38.6, CH	1.90, m	38.6, CH	1.88, m
9	99.7, C	-	99.7, C	-
10	43.5, C	-	43.5, C	-
11*α*	33.0, CH_2	3.24, d, *16.9*	33.0, CH_2	3.22, d, *16.7*
11*β*		2.86, d, *16.9*		2.84, d, *16.7*
12	16.0, CH_3	0.74, d, *6.5*	16.0, CH_3	0.73, d, *6.6*
13	29.0, CH_3	0.98, s	29.0, CH_3	0.97, s
14	23.0, CH_3	0.89, s	23.0, CH_3	0.88, s
15	16.6, CH_3	1.06, s	16.6, CH_3	1.05, s
1′	118.7, C	-	118.7, C	-
2′	155.1, C	-	155.1, C	-
3′	102.1, CH	6.69, s	102.1, CH	6.67, s
4′	114.9, C	-	114.9, C	-
5′	135.1, C	-	135.1, C	-
6′	157.6, C	-	157.6, C	-
7′	171.7, C	-	171.7, C	-
8′a	45.9, CH_2	4.74, d, *17.2*	45.9, CH_2	4.72, d, *17.2*
8′b		4.29, d, *17.2*		4.28, d, *17.2*
1′′	170.3, C	-	177.4, C	-
2′′	57.6, CH	4.76, m	57.7, CH	4.75, m
3′′a	28.8, CH_2	2.15, m	28.8, CH_2	2.15, m
3′′b		1.90, m		
4′′	27.2, CH_2	1.56, m	27.2, CH_2	1.55, m
5′′	41.9, CH_2	3.24, m	41.9, CH_2	3.24, m
6′′	158.6, CH	-	158.6, CH	-

Table S2. ^{1}H and ^{13}C NMR data of **2** (500/125 MHz, $(CD_3)_2SO$) and stachybocin A (400/100 MHz, $(CD_3)_2SO$)[33].

	**2**		**Stachybocine A**	
N°	δ_C (ppm)/mult.	δ_H (ppm)/mult./*J*(Hz)	δ_C (ppm)/mult.	δ_H (ppm)/mult./*J*(Hz)
1	167.9, C	-	168.3, C	-
2	134.1, C	-	133.2, C	-
3	100.9, CH	6.54, s	100.9, CH	6.59
4	154.0, C	-	153.8, C	-
5	116.4, C	-	116.9, C	-
6	155.9, C	-	155.9, C	-
7	112.4, C	-	112.3, C	-
8a<br>8b	43.8, CH_2	4.57, d, *16.6*<br>4.06, d, *16.6*	43.9, CH_2	4.28
9*α*<br>9*β*	31.9, CH_2	3.10, dd, *16.6*, *6.9*<br>2.73, dd, *16.6*, *6.9*	31.7, CH_2	3.11<br>2.75
10	97.8, C	-	98.3, C	-
11	36.7, CH	1.76, m	36.6, CH	1.77
12*α*<br>12*β*	30.8, CH_2	1.41, m<br>1.50, m	30.7, CH_2	1.36<br>1.51
13	20.5, CH_2	1.41, m	20.4, CH_2	1.46<br>1.38
14	39.4, CH	2.02, m	39.4, CH	2.01
15	41.8, C	-	41.8, C	-
16*α*<br>16*β*	23.9, CH_2	1.15, m<br>1.76, m	23.8, CH_2	0.92<br>1.75
17*α*<br>17*β*	25.0, CH_2	1.41, m<br>1.76, m	24.9, CH_2	1.39<br>1.75
18	73.5, CH	3.16, m	73.5, CH	3.18
19	37.4, C	-	37.3, C	-
20	15.7, CH_3	0.63, d, *6.4*	15.5, CH_3	0.64
21	15.9, CH_3	0.93, s	15.8, CH_3	0.94
22	28.8, CH_3	0.87, s	28.6, CH_3[a]	0.88[a]
23	22.5, CH_3	0.78, s	22.4, CH_3 [a]	0.79[a]
1''	166.1, C	-	172.8, C	-
2''	56.2, CH	4.36, dd, *10.9*, *4.5*	53.5, CH	4.71
3''a<br>3''b	24.3, CH_2	2.02, m<br>1.76, m	28.5, CH_2	2.03<br>1.98
4''	29.1, CH_2	1.23, m	23.4, CH_2	1.23
5''	27.6, CH_2	1.60, m	27.2, CH_2	1.64
6''a<br>6''b	41.7, CH_2	3.41, m	41.4, CH_2	3.43<br>3.38

	2		Stachybocine A	
N°	δC (ppm)/mult.	δH (ppm)/mult./*J*(Hz)	δC (ppm)/mult.	δH (ppm)/mult./*J*(Hz)
1′	167.4, C	-	167.4, C	-
2′	134.8, C	-	134.1, C	-
3′	100.7, CH	6.53, s	100.8, CH	6.53
4′	153.6, C	-	153.7, C	-
5′	115.9, C	-	116.4, C	-
6′	155.8, C	-	155.8, C	-
7′	111.6, C	-	111.9, C	-
8′a	46.6, CH_2	4.27, d, *16.9*	46.6, CH_2	4.18
8′b		4.16, d, *16.9*		
9′*α*	31.8, CH_2	3.10, dd, *16.6*, *6.9*	31.7, CH_2	3.11
9′*β*		2.73, dd, *16.6*, *6.9*		2.75
10′	97.6, C	-	97.8, C	-
11′	36.5, CH	1.76, m	36.5, CH	1.77
12′*α*	30.8, CH_2	1.41, m	30.8, CH_2	1.36
12′*β*		1.50, m		1.51
13	20.5, CH_2	1.41, m	20.4, CH_2	1.46
				1.38
14′	39.4, CH	2.02, m	39.4, CH	2.01
15′	41.8, C	-	41.8, C	-
16′*α*	23.9, CH_2	1.15, m	23.8, CH_2	0.92
16′*β*		1.76, m		1.75
17′*α*	24.9, CH_2	1.41, m	24.8, CH_2	1.39
17′*β*		1.76, m		1.75
18′	73.5, CH	3.16, m	73.5, CH	3.18
19′	37.3, C	-	37.3, C	-
20′	15.6, CH_3	0.63, d, *6.4*	15.5, CH_3	0.64
21′	15.8, CH_3	0.93, s	15.8, CH_3	0.94
22′	28.8, CH_3	0.87, s	28.6, CH_3[b]	0.88[b]
23′	22.4, CH_3	0.78, s	22.4, CH_3[b]	0.79[b]

[a] ^1H and ^{13}C chemical shifts for 22 and 23 were probably inverted by Ogawa *et al.*[6].
[b] ^1H and ^{13}C chemical shifts for 22′ and 23′ were probably inverted by Ogawa *et al.*[6].

Table S3. ^1H and ^{13}C NMR data of **3** (500/125 MHz, CD_3OD) and stachybotrin I (400/100 MHz, $(CD_3)_2SO$)[35].

	**3**		**Stachybotrin I**	
N°	δ_C (ppm)/mult.	δ_H (ppm)/mult./J(Hz)	δ_C (ppm)/mult.	δ_H (ppm)/mult./J(Hz)
1α	25.4, CH_2	1.09, m	23.8[a], CH_2	0.92[c]
1β		1.84, m		1.73[c]
2	22.1, CH_2	1.55, m	24.8[a], CH_2	1.40<br>1.81[c]
3	76.4, CH	3.35, s	73.3[a], CH	3.18[c]
4	38.6, C	-	n.d[b]	-
5	41.3, CH	2.13, m	39.3[a], CH	2.01[c]
6α	26.1, CH	1.96, m	20.4[a], CH	1.46[c]
6β		1.55, m		1.4[c]
7	32.3, CH_2	1.55, m	30.7[a], CH_2	1.39<br>1.53
8	38.5, CH	1.84, m	36.4[a], CH	1.77[c]
9	99.5, C	-	n.d[b]	-
10	43.5, C	-	n.d[b]	-
11α	33.0, CH_2	3.17, d, *16.8*	31.6[a], CH_2	3.10[c]
11β		2.80, d, *16.8*		2.73[c]
12	16.0, CH_3	0.67, d, *6.5*	15.5[a], CH_3	0.59[c]
13	29.0, CH_3	0.98, s	28.6[a], CH_3	0.88[c]
14	23.0, CH_3	0.88, s	22.3[a], CH_3	0.79[c]
15	16.6, CH_3	1.04, s	15.8[a], CH_3	0.94[c]
1′	118.5, C	-	n.d[b]	-
2′	154.8, C	-	n.d[b]	-
3′	102.0, CH	6.57, s	100.8[a], CH	6.49[c]
4′	114.9, C	-	n.d.	-
5′	135.3, C	-	n.d.	-
6′	157.5, C	-	n.d.	-
7′	174.5, C	-	n.d.	-
8′a	46.3, CH_2	4.67, d, *17.0*	44.30[a], CH_2	4.26[c]
8′b		4.22, d, *17.0*		4.26[c]
1″	170.3, C	-	n.d[b]	-
2″	59.7, CH	5.13, m	54.5[a], CH	5.11[c]
3″a	38.0, CH_2	3.54, m	34.4[a], CH_2	3.33[c]
3″b		3.21, m		3.27[c]
4″	140.2, C	-	n.d[b]	-
5″	129.6, CH	7.26, d, *7.3*	128.2[a], CH	7.22[c]
6″	129.4, CH	7.19, t, *7.3*	128.4[a], CH	7.25[c]
7″	127.3, CH	7.10, t, *7.3*	126.4[a], CH	7.14[c]

[a] ^{13}C chemical shifts were determined from the HMQC spectrum.
[b] ^{13}C chemical shifts were not determined for quaternary carbons.
[c] Multiplicity were not reported for the protons in the ^1H NMR spectrum.

Table S4. ^{1}H and ^{13}C NMR data of **4** (500/125 MHz, CD_3OD) and stachybotrin H (400/100 MHz, $(CD_3)_2SO$)[37].

	**4**		**Stachybotrin H**	
N°	δ_C (ppm)/mult.	δ_H (ppm)/mult./*J*(Hz)	δ_C (ppm)/mult.	δ_H (ppm)/mult./*J*(Hz)
1*α*	25.4, CH_2	1.09, m	24.3, CH_2	0.93, m
1*β*		1.86, m		1.76, m
2	22.1, CH_2	1.64, m	25.3, CH_2	1.35, m<br>1.73, m
3	76.5, CH	3.33, s	73.9, CH	3.17, bs
4	38.6, C	-	36.9, C	-
5	41.3, CH	2.14, m	40.5, CH	2.02, d, *8.9*
6*α*	26.1, CH_2	1.96, m	20.9, CH	1.44, m
6*β*		1.59, m		1.40, m
7	32.3, CH_2	1.59, m	31.3, CH_2	1.53, m<br>1.37, m
8	38.4, CH	1.86, m	37.7, CH	1.78, m
9	99.6, C	-	98.3, C	-
10	43.5, C	-	42.2, C	-
11*α*	33.0, CH_2	3.22, d, *16.8*	32.1, CH_2	3.12, d, *17.0*
11*β*		2.84, d, *16.8*		2.76, d, *17.0*
12	15.9, CH_3	0.73, d, *6.5*	15.9, CH_3	0.65, d, *6.4*
13	28.9, CH_3	0.98, s	29.1, CH_3	0.87, s
14	23.0, CH_3	0.88, s	22.8, CH_3	0.79, s
15	16.6, CH_3	1.05, s	16.2, CH_3	0.95, s
1′	118.6, C	-	117.1, C	-
2′	155.1, C	-	154.2, C	-
3′	102.2, CH	6.68, s	101.4, CH	6.57, s
4′	114.8, C	-	112.6, C[a]	-
5′	135.2, C	-	133.9, C[a]	-
6′	157.5, C	-	156.3, C	-
7′	174.7, C	-	168.2, C[b]	-
8′a	47.4, CH_2	4.23, d, *17.0*	47.8, CH_2	4.30, s
8′b		4.07, d, *17.0*		
1′′	170.4, C	-	171.4, C[b]	-
2′′	49.3, CH_2	4.48, m	44.3, CH_2	4.14, d, *17.6*<br>4.10, d, *17.6*

[a] ^{13}C chemical shifts for 5′ and 6′ were probably inverted by Ma *et al.*[4].
[b] ^{13}C chemical shifts for 7′ and 1′′ were probably inverted by Ma *et al.*[4].

Table S5. ^1H and ^{13}C NMR data of **5** (500/125 MHz, CD_3OD) and stachybotrylactam (($(CD_3)_2CO$, 200 MHz; $CDCl_3$, 50 MHz)[36].

	**5**		**Stachybotrylactam**	
N°	δ_C (ppm)/mult.	δ_H (ppm)/mult./J(Hz)	δ_C (ppm)/mult.	δ_H (ppm)/mult./J(Hz)
1α	25.4, CH_2	1.06, m	24.9, CH_2	1.90-1.05, m
1β		1.84, m		
2	22.1, CH_2	1.54, m	21.7, CH_2	1.90-1.50, m
3	76.5, CH	3.34, s	75.5, CH	3.36, bs
4	38.7, C	-	38.3, C	-
5	41.4, CH	2.13, m	40.6, CH	2.20, m
6α	26.0, CH	1.97, m	25.9, CH	1.90-1.50, m
6β		1.54, m		
7	32.3, CH_2	1.54, m	31.9, CH_2	1.90-1.50, m
8	38.4, CH	1.86, m	37.7, CH	1.90-1.50, m
9	99.7, C	-	99.0, C	-
10	43.5, C	-	43.0, C	-
11α	33.0, CH	3.24, d, *16.9*	32.6, CH	3.18, d, *17.2*
11β		2.86, d, *16.9*		2.83, d, *17.2*
12	16.0, CH_3	0.74, d, *6.5*	16.0, CH_3	0.73, d, *6.0*
13	29.0, CH_3	0.99, s	28.6, CH_3	0.98, s
14	23.0, CH_3	0.89, s	22.8, CH_3	0.87, s
15	16.5, CH_3	1.06, s	16.4, CH_3	1.03, s
1′	119.0, C	-	118.0, C	-
2′	155.2, C	-	153.9, C	-
3′	102.1, CH	6.69, s	102.3, CH	6.80, s
4′	116.7, C	-	116.0, C	-
5′	134.7, C	-	135.0, C	-
6′	157.8, C	-	154.7, C	-
7′	174.1, C	-	172.1, C	-
8′a	43.9, CH	4.43, d, *17.4*	43.3, CH	4.35, d, *16.5*
8′b		4.27, d, *17.4*		4.25, d, *16.5*

Table S6. ^{1}H and ^{13}C NMR data of **6** (500/125 MHz, CD_3OD) and stachybotrylactam acetate (200/50 MHz, $CDCl_3$)[36].

	**6**		**Stachybotrylactam acetate**	
N°	δ_C (ppm)/mult.	δ_H (ppm)/mult./*J*(Hz)	δ_C (ppm)/mult.	δ_H (ppm)/mult./*J*(Hz)
1α	25.9, CH_2	1.15, m	24.9, CH_2	1.90-1.40, m
1β		1.71, m		
2	21.9, CH_2	1.55, m	20.8, CH_2	1.90-1.40, m
3	79.5, CH	4.60, s	77.9, CH	4.58, s
4	37.8, C	-	36.8, C	-
5	42.3, CH	2.16, m	40.9, CH	2.00, m
6α	23.4, CH_2	1.87, m	22.3, CH_2	1.90-1.40, m
6β		1.55, m		
7	32.2, CH	1.55, m	30.8, CH	1.90-1.40, m
8	38.2, CH	1.87, m	37.0, CH	1.90-1.40, m
9	99.6, C	-	98.6, C	-
10	43.5, C	-	42.0, C	-
11α	32.9, CH	3.23, d, *17.0*	31.8, CH	3.19, d, *17.0*
11β		2.88, d, *17.0*		2.84, d, *17.0*
12	15.9, CH_3	0.76, d, *6.5*	15.5, CH_3	0.70, d, *6.0*
13	28.4, CH_3	0.97, s	27.8, CH_3	0.97, s
14	22.3, CH_3	0.92, s	21.2, CH_3	0.89, s
15	16.4, CH_3	1.08, s	16.0, CH_3	1.23, s
16	172.4, C	-	170.4, C	-
17	21.1, CH_3	2.03, s	21.7, CH_3	1.99, s
1′	118.9, C	-	117.8, C	-
2′	155.4, C	-	153.3, C	-
3′	102.3, CH	6.70, s	102.3, CH	6.90, s
4′	116.2, C	-	115.3, C	-
5′	134.9, C	-	135.0, C	-
6′	157.6, C	-	156.4, C	-
7′	174.0, C	-	172.4, C	-
8′a	43.9, CH	4.35, d, *17.2*	43,0, CH	4.38, d, *15.0*
8′b		4.23, d, *17.2*		4.22, d, *15.0*

Table S7. ^{1}H and ^{13}C NMR data of **7** (500/125 MHz, CD_3OD) and 2*α*-acetoxystachybotrylactam acetate (200/50 MHz, $CDCl_3$)[36].

	**7**		**2*α*-acetoxystachybotrylactam acetate**	
N°	δ_C (ppm)/mult.	δ_H (ppm)/mult./*J*(Hz)	δ_C (ppm)/mult.	δ_H (ppm)/mult./*J*(Hz)
1*α*	31.6, CH	1.40, m	30.5, CH	1.80-1.40, m
1*β*		1.82, m		
2	69.6, CH	5.23, m	68.2, CH	5.21, bd, *11.0*
3	78.4, CH	4.95, s	76.7, CH	4.95, bs
4	39.1, C	-	38.1, C	-
5	41.9, CH	2.13, m	40.5, CH	2.05, m
6	21.5, CH_2	1.66-1.54, m	20.4, CH_2	1.80-1.40, m
7	32.1, CH	1.66-1.54, m	30.7, CH	1.80-1.40, m
8	37.8, CH	1.91, m	36.7, CH	1.80-1.40, m
9	99.2, C	-	98.1, C	-
10	44.8, C	-	43.5, C	-
11*α*	33.0, CH	3.24, d, *17.1*	32.0, CH	3.24, d, 16.5
11*β*		2.93, d, *17.1*		2.90, d, 16.5
12	15.8, CH_3	0.78, d, *6.5*	15.4, CH_3	0.71, d, 6.4
13	28.3, CH_3	1.05, s	27.7, CH_3	1.02, s
14	22.0, CH_3	0.94, s	20.9, CH_3	0.93, s
15	17.3, CH_3	1.16, s	16.9, CH_3	1.10, s
16	172.3, C	-	170.3, C	-
17	20.9, CH_3	2.07, s	21.0, CH_3	2.07, s
18	172.4, C	-	170.3, C	-
19	20.8, CH_3	1.86, s	21.6, CH_3	1.90, s
1′	118.6, C	-	117.6, C	-
2′	155.5, C	-	153.4, C	-
3′	102.5, CH	6.73, s	102.5, CH	7.01, s
4′	116.9, C	-	115.3, C	-
5′	135.1, CH	-	133.0, CH	-
6′	157.4, C	-	156.1, C	-
7′	173.8, C	-	172.0, C	-
8′a	43.8, CH	4.36, d, *17.2*	43.5, CH	4.42, d, *16.5*
8′b		4.24, d, *17.2*		4.25, d, *16.5*

Table S8. ^{1}H NMR data of **8** (500 MHz, CD_3OD) and stachybotramide (50 MHz, C_5D_6N)[36].

	**8**	**Stachybotramide**
**N°**	δ_H (ppm)/mult./*J*(Hz)	δ_H (ppm)/mult./*J*(Hz)
1α	1.10, m	1.90-1.20, m
1β	1.86, m	1.90-1.20, m
2	1.57, m	1.90-1.20, m
3	3.35, s	3.60, bs
4	-	-
5	2.14, m	2.05, m
6α	1.97, m	1.90-1.20, m
6β	1.57, m	1.90-1.20, m
7	1.57, m	1.90-1.20, m
8	1.86, m	1.90-1.20, m
9	-	-
10	-	-
11α	3.23, d, *16.9*	3.52, d, *17.0*
11β	2.85, d, *16.9*	3.10, d, *17.0*
12	0.73, d, *6.5*	0.84, d, *5.5*
13	0.98, s	0.98, s
14	0.89, s	0.91, s
15	1.06, s	1.22, s
1′	-	-
2′	-	-
3′	6.67, s	7.35, s
4′	-	-
5′	-	-
6′	-	-
7′	-	-
8′a	4.57, d, *17.2*	4.35, d, *16.8*
8′b	4.43, d, *17.2*	4.07, d, *16.8*
1′′a<br>1′′b	3.70, t, *5.4*	3.65, m
2′′	3.80, t, *5.4*	3.90, m

Table S9. ^{1}H and ^{13}C NMR data of **9** (500/125 MHz, CD_3OD) and chartarlactam B (500/125 MHz, $(CD_3)_2SO$)[16].

	9		Chartarlactame B	
N°	δ_C (ppm)/mult.	δ_H (ppm)/mult./*J*(Hz)	δ_C (ppm)/mult.	δ_H (ppm)/mult./*J*(Hz)
1α	25.4, CH_2	1.08, m	24.3, CH_2	0.91, m
1β		1.84, m		1.75, m
2	22.1, CH_2	1.59, m	25.3, CH_2	1.78, m<br>1.38, m
3	76.4, CH	3.33, s	73.9, CH	3.17, bs
4	38.5, C	-	37.8, C	-
5	41.3, CH	2.13, m	39.8, CH	2.01, brd, *9.8*
6α	26.0, CH_2	1.96, m	20.9, CH_2	1.49, m
6β		1.51, m		1.45, m
7	32.3, CH_2	1.59, m	31.2, CH_2	1.52, m<br>1.39, m
8	38.4, CH	1.84, m	36.9, CH	1.78, m
9	99.7, C	-	98.3, C	-
10	43.5, C	-	42.3, C	-
11α	32.9, CH_2	3.22, d, *16.9*	32.1, CH_2	3.11, d, *17.0*
11β		2.85, d, *16.9*		2.76, d, *17.0*
12	16.0, CH_3	0.74, d, *6.5*	16.0, CH_3	0.64, d, *6.1*
13	29.0, CH_3	0.98, s	29.1, CH_3	0.88, s
14	23.0, CH_3	0.89, s	22.8, CH_3	0.80, s
15	16.5, CH_3	1.05, s	16.3, CH_3	0.95, s
1′	118.6, C	-	116.9, C	-
2′	155.2, C	-	154.1, C	-
3′	102.1, CH	6.65, s	101.3, CH	6.55, s
4′	114.3, C	-	134.5, C	-
5′	135.2, C	-	112.3, C	-
6′	157.6, C	-	156.3, C	-
7′	171.7, C	-	167.9, C	-
8′a	43.6, CH_2	4.47, d, *17.2*	47.0, CH_2	4.32, d, *17.0*
8′b		4.33, d, *17.2*		4.21, d, *17.0*
1′′	43.5, CH_2	3.61, t, *7.0*	41.8, CH_2	3.46, t, *7.0*
2′′	29.5, CH_2	1.73, m	27.7, CH_2	1.60, m
3′′	25.0, CH_2	1.67, m	22.4, CH_2	1.46, m
4′′	38.7, CH_2	2.23, t, *7.3*	33.9, CH_2	2.24, t, *7.0*
5′′	170.3, C	-	175.3, C	-

Table S10. ^1H NMR data of **10** (500 MHz, CD_3OD) and F1839-J (270 MHz, C_5D_6N)[6].

	**10**	**F1839-J**
**N°**	δ_H (ppm)/mult./*J*(Hz)	δ_H (ppm)/mult./*J*(Hz)
1*α*	1.10, m	1.20, dt, *12.2*, *3.3*
1*β*	1.88, m	2.36, m
2	1.54, m	1.77-2.07, m
3	3.35, m	3.63, m
4	-	-
5	2.11, m	2.54, m
6*α*	1.93, m	1.71, m
6*β*	1.54, m	1.44, m
7	1.60, m	2.00, m<br>1.62, m
8	1.83, m	2.00, m
9	-	-
10	-	-
11*α*	3.22, d, *16.9*	3.58, d, *17.1*
11*β*	2.85, d, *16.9*	2.92, d, *17.1*
12	0.73, d, *6.5*	0.92, d, *5.4*
13	0.99, s	1.28, s
14	0.89, s	0.95, s
15	1.06, s	1.04, s
1′	-	-
2′	-	-
3′	6.67, s	7.40, s
4′	-	-
5′	-	-
6′	-	-
7′	-	-
8′a	4.69, d, *16.9*	3.84, d, *16.6*
8′b	4.24, d, *16.9*	4.08, d, *16.6*
1′′a	3.87, m	3.34, m
1′′b	3.78, m	3.58, m
2′′	1.83, m	1.45, m
3′′	1.60, m	1.58, m
4′′	2.33, m	2.35, m
5′′	-	-
6′′	3.65, m	3.61, s

3. TD-DFT and ECD

Table S11. Coordinates (Ångstroms) for conformer (2′′*S*, 3*R*, 5*S*, 8*R*, 9*R*, 10*S*)-**1**_C1 (Energy: -1800.701682 Hartree, Solvent: CH_3OH, Boltzmann %: 58.3).

Symbol	X	Y	Z
C	3.8968710	0.3158420	0.4409050
C	3.7285060	1.4779510	-0.6069280
C	3.6382750	2.8426760	0.0961770
C	2.4712350	2.8774880	1.0857950
C	2.4882380	1.7266010	2.1051020
C	2.6462350	0.3532410	1.4035350
C	3.9311320	-1.0371850	-0.3151310
C	4.9470630	-1.0635700	-1.4622500
C	4.7006390	0.0502270	-2.4801570
C	4.7013110	1.4617910	-1.8396640
O	1.4300620	0.1620990	0.5673040
C	0.6996310	-0.8640950	1.0603200
C	1.2916470	-1.4988390	2.1511410
C	2.6132590	-0.8342380	2.4268770
C	-0.5231480	-1.2908570	0.5664290
C	-1.1338250	-2.3694500	1.2079230
C	-0.5789840	-3.0286110	2.2993650
C	0.6596300	-2.5790090	2.7712350
C	-1.3794400	-0.8079550	-0.5679150
N	-2.5346950	-1.7080240	-0.4790840
C	-2.4189680	-2.6475440	0.5185050
O	3.4316600	-0.1492270	-3.1391220
H	2.7448550	1.2980100	-1.0519130
C	1.2520550	1.8217500	3.0130240
C	6.1603640	1.8533600	-1.5139160
C	5.1719510	0.4390030	1.3131810
O	-3.2377890	-3.5308860	0.7643470
O	1.2055170	-3.2345290	3.8426260
C	4.2026270	2.4733580	-2.8989780
C	-3.6343560	-1.7139460	-1.4199340
C	-4.2699160	-0.3223100	-1.6284330
C	-3.2141110	-2.3535260	-2.7486770
O	-4.2620710	-2.4716300	-3.5846620
O	-2.1003440	-2.7276030	-3.0278530
C	-4.8706750	0.2513700	-0.3416790
C	-5.4946760	1.6274860	-0.5730530
N	-6.0617860	2.1400160	0.6773160
C	-6.7271660	3.3477130	0.7703890
N	-6.3368390	4.3118080	-0.1449430
N	-7.6556000	3.4887530	1.6609610
H	3.4925830	3.6355970	-0.6409690
H	4.5718440	3.0795200	0.6159070
H	2.4687010	3.8276980	1.6302770
H	1.5274830	2.8350830	0.5299200
H	3.3750130	1.8405740	2.7397060

H	2.9406390	-1.2399240	-0.7271110
H	4.1641220	-1.8537440	0.3744500
H	4.8931230	-2.0316430	-1.9739380
H	5.9711940	-0.9877590	-1.0858760
H	5.4915480	0.0275880	-3.2423520
H	3.4429790	-1.5269350	2.2716990
H	2.6990320	-0.4663310	3.4526570
H	-1.0783190	-3.8631210	2.7753760
H	-0.8871390	-0.9073250	-1.5400260
H	-1.6818630	0.2370250	-0.4440260
H	3.4325870	-1.0345110	-3.5195520
H	1.2273900	2.8051420	3.4903980
H	1.2472820	1.0749450	3.8097230
H	0.3302690	1.7116890	2.4361120
H	6.6805620	1.1305010	-0.8874370
H	6.1989140	2.8201840	-1.0072460
H	6.7306220	1.9502820	-2.4431050
H	6.0602530	0.1409880	0.7590730
H	5.3438230	1.4501960	1.6809640
H	5.1160860	-0.2190160	2.1836030
H	2.0498970	-2.8380020	4.0868120
H	4.7352070	2.3181310	-3.8424260
H	3.1353600	2.3638820	-3.0961970
H	4.3928610	3.5015560	-2.5836570
H	-4.3899760	-2.3820230	-0.9947470
H	-3.5224140	0.3653700	-2.0378590
H	-5.0488890	-0.4233860	-2.3866570
H	-3.9561140	-2.8889020	-4.4060820
H	-4.1059680	0.3327180	0.4364550
H	-5.6367190	-0.4362540	0.0356120
H	-6.2582940	1.5649760	-1.3602610
H	-4.7175810	2.3103260	-0.9279530
H	-6.5115540	1.4383740	1.2510190
H	-6.6365590	5.2525340	0.0658980
H	-5.3890920	4.2703980	-0.4921920
H	-7.9900040	4.4495820	1.6859300

Table S12. Coordinates (Ångstroms) for conformer (2′′*S*, 3*R*, 5*S*, 8*R*, 9*R*, 10*S*)-**1**_C2 (Energy: -1800.70038 Hartree, Solvent: CH_3OH, Boltzmann %: 14.6).

Symbol	X	Y	Z
C	4.2545900	-0.2591160	-0.2593760
C	3.9079500	-1.7898800	-0.3751620
C	3.9954600	-2.2644660	-1.8353140
C	3.0531060	-1.4605280	-2.7342970
C	3.2465080	0.0611700	-2.6310640
C	3.2254990	0.5345740	-1.1549640
C	4.1012810	0.1767820	1.2204870
C	4.8642590	-0.7234420	2.1985870
C	4.4468080	-2.1897030	2.0827630
C	4.6121790	-2.7513050	0.6476420
O	1.8608540	0.2440810	-0.6363550
C	1.2181200	1.4061250	-0.3814190
C	2.0016800	2.5384740	-0.5984680
C	3.3693640	2.0914990	-1.0378900
C	-0.0911340	1.5216030	0.0582010
C	-0.5876600	2.8089380	0.2687570
C	0.1592280	3.9633490	0.0620070
C	1.4798150	3.8148990	-0.3781680
C	-1.1479940	0.5003940	0.3636380
N	-2.2774620	1.3464240	0.7662620
C	-1.9955390	2.6933220	0.7269530
O	3.0665900	-2.3461140	2.4762730
H	2.8506240	-1.8480650	-0.0982260
C	2.2212440	0.7740290	-3.5268060
C	6.1165990	-2.9845010	0.3818960
C	5.6819280	0.0983720	-0.7457260
O	-2.7775860	3.5950540	1.0159360
O	2.2142630	4.9535870	-0.5751820
C	3.9364910	-4.1422160	0.5915290
C	-3.5781740	0.8284530	1.1680870
C	-4.2739320	0.0281740	0.0592700
C	-3.4072430	0.0168330	2.4612380
O	-3.1455100	0.8161180	3.5104290
O	-3.4694630	-1.1868670	2.5453250
C	-5.7581240	-0.2256090	0.3468190
C	-6.4258800	-1.0219000	-0.7748630
N	-7.8399750	-1.2325070	-0.4720830
C	-8.7146550	-1.9418820	-1.2659770
N	-8.1349920	-2.8677070	-2.1187210
N	-9.9892060	-1.7683870	-1.1073220
H	3.7240070	-3.3203570	-1.9034480
H	5.0202840	-2.1898240	-2.2121520
H	3.1845680	-1.7600990	-3.7794390
H	2.0158330	-1.6977090	-2.4710030
H	4.2456300	0.3085680	-3.0088970
H	3.0432150	0.1587740	1.4891990

H	4.4474850	1.2059370	1.3528820
H	4.6827410	-0.3821130	3.2245170
H	5.9448930	-0.6432760	2.0498010
H	5.0673710	-2.7984730	2.7545380
H	4.1298360	2.3762900	-0.3079720
H	3.6731890	2.5268940	-1.9932160
H	-0.2558430	4.9486030	0.2327650
H	-0.8554080	-0.1783170	1.1727700
H	-1.4045240	-0.1099710	-0.5082980
H	2.9696420	-1.9815940	3.3629480
H	2.3194350	0.4081780	-4.5525020
H	2.3564770	1.8572410	-3.5551090
H	1.1997020	0.5657290	-3.1985910
H	6.7333670	-2.1013310	0.5408230
H	6.2818520	-3.3248340	-0.6427010
H	6.4899250	-3.7677710	1.0490380
H	6.4322320	-0.1819110	-0.0086460
H	5.9502570	-0.3890140	-1.6826440
H	5.7858130	1.1754360	-0.8973260
H	3.0990870	4.7353970	-0.8907060
H	4.2631810	-4.7499920	1.4410290
H	2.8488080	-4.0689640	0.6320140
H	4.2135570	-4.6793880	-0.3181450
H	-4.1702650	1.7131300	1.4166490
H	-4.1717990	0.6022620	-0.8663790
H	-3.7567780	-0.9241730	-0.0881080
H	-3.0024300	0.2622460	4.2951410
H	-6.2749200	0.7343360	0.4638330
H	-5.8768740	-0.7734990	1.2858880
H	-5.9292740	-1.9932350	-0.8610480
H	-6.2926330	-0.4929350	-1.7295480
H	-8.3012470	-0.4768130	0.0151100
H	-7.1953830	-2.6957240	-2.4448720
H	-8.7420600	-3.2286220	-2.8394910
H	-10.5159280	-2.3872990	-1.7191220

Table S13. Coordinates (Ångstroms) for conformer (2′′*S*, 3*R*, 5*S*, 8*R*, 9*R*, 10*S*)-**1**_C3 (Energy: -1800.700959 Hartree, Solvent: CH_3OH, Boltzmann %: 27.1).

Symbol	X	Y	Z
C	-4.0309710	-0.6435190	0.0704450
C	-4.1629530	0.2597880	-1.2116470
C	-4.2446150	-0.6004280	-2.4838260
C	-3.0132550	-1.4988930	-2.6215090
C	-2.7363270	-2.3642890	-1.3815380
C	-2.7214240	-1.5103110	-0.0875940
C	-3.9021620	0.2721480	1.3148360
C	-5.0006010	1.3377430	1.3978140
C	-5.0440790	2.2235450	0.1525160
C	-5.2276340	1.4129590	-1.1559700
O	-1.5738850	-0.5709930	-0.2123630
C	-0.6322350	-0.8842280	0.7070040
C	-1.0020430	-1.9288650	1.5525400
C	-2.3894410	-2.3742450	1.1779110
C	0.5975070	-0.2596120	0.8442870
C	1.4458260	-0.7259000	1.8496210
C	1.1161540	-1.7650120	2.7129390
C	-0.1364900	-2.3693560	2.5561430
C	1.2483650	0.8669900	0.0956870
N	2.5536480	0.9575500	0.7600430
C	2.6940870	0.0765610	1.8069910
O	-3.8265430	2.9918630	0.0446110
H	-3.2081940	0.7922160	-1.2643130
C	-1.4548260	-3.1839470	-1.5999890
C	-6.6959650	0.9381050	-1.2381640
C	-5.2211650	-1.6138560	0.2786620
O	3.6747520	-0.0034570	2.5437170
O	-0.4614150	-3.3877250	3.4123980
C	-5.0085540	2.3695890	-2.3521980
C	3.5430210	1.9739360	0.4721690
C	3.8505530	2.1132820	-1.0372540
C	3.1408500	3.3188650	1.0881070
O	4.1182970	4.2324730	0.9429300
O	2.0965870	3.5512340	1.6487000
C	4.3938270	0.8354300	-1.6896190
C	5.7966710	0.4497610	-1.2163330
N	6.2701750	-0.7171350	-1.9533370
C	7.4328110	-1.3636240	-1.5953680
N	7.8559380	-2.2963980	-2.5422930
N	8.0461870	-1.0757180	-0.4959180
H	-4.3081790	0.0399600	-3.3665040
H	-5.1519740	-1.2124400	-2.4869730
H	-3.1231360	-2.1565460	-3.4903010
H	-2.1328160	-0.8747350	-2.8132260
H	-3.5658190	-3.0712360	-1.2619880
H	-2.9342290	0.7764120	1.2871840

H	-3.9262310	-0.3242580	2.2315190
H	-4.8298590	1.9687160	2.2780010
H	-5.9831760	0.8822120	1.5501420
H	-5.8867230	2.9238270	0.2339470
H	-3.0945260	-2.1922950	1.9916030
H	-2.4461710	-3.4410650	0.9462600
H	1.7966150	-2.1022560	3.4846650
H	0.6994570	1.8087170	0.1937500
H	1.3609630	0.6477760	-0.9708980
H	-3.7086270	3.4761250	0.8692790
H	-1.5556770	-3.7783140	-2.5122000
H	-1.2448240	-3.8776520	-0.7832310
H	-0.5869950	-2.5314220	-1.7255450
H	-7.0252100	0.3734280	-0.3672420
H	-6.8525720	0.3112410	-2.1187760
H	-7.3573220	1.8049030	-1.3342040
H	-6.0937950	-1.0917590	0.6669690
H	-5.5270940	-2.1195670	-0.6367320
H	-4.9730210	-2.3863570	1.0104500
H	-1.3438740	-3.7245290	3.2177680
H	-5.5902590	3.2854120	-2.2086400
H	-3.9606570	2.6529090	-2.4605090
H	-5.3428170	1.9153360	-3.2874790
H	4.4507720	1.6701530	1.0010150
H	2.9419930	2.4270310	-1.5595070
H	4.5709670	2.9250580	-1.1582510
H	3.8271080	5.0637200	1.3512350
H	4.4166170	1.0027190	-2.7723650
H	3.7118460	-0.0028930	-1.5176610
H	5.7998370	0.1931790	-0.1561460
H	6.4814430	1.3002150	-1.3412210
H	6.0763330	-0.7040760	-2.9454010
H	8.5489020	-2.9520520	-2.2100330
H	7.1241830	-2.7538240	-3.0708380
H	8.8331630	-1.7049510	-0.3530030

Table S14. Coordinates (Ångstroms) for conformer (2′′*S*, 3*R*, 5*S*, 8*R*, 9*R*, 10*S*)-**1**_C4 (Energy: -1800.691933 Hartree, Solvent: CH_3OH, Boltzmann %: $1.91.10^{-2}$).

Symbol	X	Y	Z
C	4.2630870	-0.2515130	-0.2505340
C	3.9159220	-1.7800330	-0.3910560
C	4.0118980	-2.2330900	-1.8575240
C	3.0763720	-1.4146540	-2.7505880
C	3.2701730	0.1051830	-2.6233930
C	3.2402230	0.5562660	-1.1406020
C	4.1011100	0.1628750	1.2346050
C	4.8560690	-0.7529720	2.2044280
C	4.4377740	-2.2167650	2.0638350
C	4.6130860	-2.7569820	0.6217660
O	1.8718270	0.2582620	-0.6351680
C	1.2299770	1.4160160	-0.3612680
C	2.0151060	2.5513730	-0.5548670
C	3.3836960	2.1111610	-0.9982160
C	-0.0802800	1.5253550	0.0768630
C	-0.5756700	2.8085070	0.3113440
C	0.1727890	3.9658850	0.1281730
C	1.4942580	3.8241540	-0.3114900
C	-1.1362180	0.4983580	0.3647630
N	-2.2671140	1.3362290	0.7844290
C	-1.9830200	2.6866950	0.7664090
O	3.0544860	-2.3774610	2.4445900
H	2.8569700	-1.8415180	-0.1211000
C	2.2508660	0.8322520	-3.5144690
C	6.1192010	-2.9868460	0.3628650
C	5.6937020	0.1116990	-0.7228650
O	-2.7651840	3.5829680	1.0668160
O	2.2305740	4.9650840	-0.4845940
C	3.9375280	-4.1466650	0.5401170
C	-3.5756780	0.8295780	1.1762070
C	-4.2876040	0.0615770	0.0498250
C	-3.4343670	0.0766840	2.5185200
O	-3.2401690	-1.2586210	2.4835200
O	-3.4524060	0.6427420	3.5823870
C	-5.7585020	-0.2394950	0.3644030
C	-6.4515880	-0.9603120	-0.7929010
N	-7.8487370	-1.2223370	-0.4581360
C	-8.7349050	-1.9000970	-1.2681560
N	-8.1645760	-2.7489090	-2.2034730
N	-10.0057090	-1.7729100	-1.0506550
H	3.7394300	-3.2874610	-1.9429970
H	5.0391810	-2.1544780	-2.2267480
H	3.2145910	-1.6986490	-3.7991960
H	2.0371070	-1.6547020	-2.4979400
H	4.2717890	0.3575370	-2.9911680
H	3.0413930	0.1432010	1.4965510

H	4.4484500	1.1892590	1.3842500
H	4.6677110	-0.4267460	3.2340130
H	5.9378120	-0.6720500	2.0644780
H	5.0526890	-2.8362010	2.7310420
H	4.1424920	2.3839240	-0.2620220
H	3.6894900	2.5624100	-1.9455280
H	-0.2413330	4.9480110	0.3179520
H	-0.8377880	-0.1923680	1.1613030
H	-1.3913590	-0.0951350	-0.5195050
H	2.9500440	-2.0217530	3.3340210
H	2.3547320	0.4816480	-4.5449060
H	2.3872880	1.9156250	-3.5259210
H	1.2272290	0.6201440	-3.1953060
H	6.7351990	-2.1064080	0.5388690
H	6.2913110	-3.3122360	-0.6654540
H	6.4877100	-3.7799500	1.0209970
H	6.4391200	-0.1795050	0.0149550
H	5.9675140	-0.3626980	-1.6648420
H	5.7993370	1.1906990	-0.8586570
H	3.1175440	4.7516230	-0.7973800
H	4.2575370	-4.7669290	1.3831000
H	2.8495730	-4.0737350	0.5733110
H	4.2214660	-4.6705480	-0.3751770
H	-4.1585730	1.7197780	1.4190070
H	-4.2231800	0.6862410	-0.8457070
H	-3.7577860	-0.8662710	-0.2011310
H	-3.2719780	-1.6097160	1.5825500
H	-6.2799720	0.7022680	0.5687300
H	-5.8495070	-0.8546010	1.2655450
H	-5.9420690	-1.9119270	-0.9719180
H	-6.3602460	-0.3553510	-1.7063160
H	-8.3098670	-0.5108510	0.0917900
H	-8.7921610	-3.0828340	-2.9197520
H	-7.2478950	-2.5219680	-2.5600090
H	-10.5410870	-2.3592590	-1.6865160

Table S15. Coordinates (Ångstroms) for conformer (2′′*R*, 10*R*, 10′*R*, 13*R*, 13′*R*, 14*S*, 14′*S*, 15*S*, 15′*S*, 18*R*, 18′*R*)-**2**_C1 (Energy: -2885.29118 Hartree, Solvent: CH_3OH, Boltzmann %: 44.8).

Symbol	X	Y	Z
C	5.0835480	-3.0947280	0.9635610
C	4.1784870	-3.5986850	-0.2214480
C	5.0301690	-4.2420340	-1.3287400
C	6.0703960	-3.2567750	-1.8669680
C	6.9554330	-2.6331060	-0.7757310
C	6.1009610	-2.0413790	0.3744610
C	4.1826770	-2.4119080	2.0245560
C	2.9928060	-3.2766490	2.4556380
C	2.1168690	-3.6875650	1.2717700
C	2.9082850	-4.4354390	0.1685780
O	5.3056420	-0.9309820	-0.2163460
C	5.7166700	0.2468760	0.3067950
C	6.7030170	0.1115890	1.2824480
C	6.9883090	-1.3538280	1.4688580
C	5.2378330	1.4971190	-0.0519540
C	5.7810660	2.6044070	0.6011460
C	6.7670540	2.5134380	1.5775440
C	7.2295180	1.2373630	1.9194620
C	4.2030990	1.9365330	-1.0465970
N	4.2103450	3.3925300	-0.8676230
C	5.1302520	3.8237690	0.0584420
O	1.5026350	-2.5224600	0.6800450
C	3.2077600	-5.8682350	0.6641920
H	3.7620770	-2.6819040	-0.6505040
C	7.9256050	-1.6249930	-1.4115510
C	1.9916090	-4.5826700	-1.0692770
C	5.9044990	-4.2177480	1.6463610
O	5.3471000	4.9967920	0.3565490
O	8.1971590	1.1507450	2.8850010
N	-2.2956200	3.3691790	1.0212390
C	-2.8254100	2.0109110	0.8892370
C	-4.3078420	2.2425690	0.9433370
C	-4.5703500	3.6054090	1.0933650
C	-3.2674950	4.3238380	1.1415590
C	-5.3954680	1.3864560	0.8756030
C	-6.6984350	1.8761690	0.9553760
C	-6.9319610	3.2444750	1.1056370
C	-5.8533200	4.1341210	1.1783180
O	-5.3296670	0.0427620	0.7273570
C	-6.7026580	-0.5289740	0.7596910
C	-7.6503350	0.7186870	0.8206520
C	-6.7924690	-1.3886280	2.0466520
C	-5.8219800	-2.5797940	1.9932780
C	-5.9324120	-3.4095620	0.7118190
C	-5.7944150	-2.5223230	-0.5368130
C	-6.8901310	-1.3932000	-0.5481070

C	-5.5996560	-3.3291450	-1.8701190
C	-5.4545930	-2.3423490	-3.0566970
C	-6.5710030	-1.2985910	-3.1010330
C	-6.6721700	-0.5004040	-1.7966350
O	-4.1694930	-1.6924440	-2.9542110
H	-4.8507450	-1.9796980	-0.4227080
C	-6.7388890	-4.3218360	-2.1947700
C	-4.3020740	-4.1667520	-1.7760480
C	-6.5629220	-0.5934100	3.3414500
C	-8.3434330	-1.9312330	-0.5584280
O	-8.1947920	3.7703400	1.1849810
O	-3.0781800	5.5349500	1.2681080
C	3.4206170	4.3126500	-1.6586760
C	1.9171050	3.9596410	-1.6923290
C	1.2679070	3.9767410	-0.3046410
C	-0.2297400	3.6566560	-0.3597100
C	-0.8698760	3.6598550	1.0355460
C	4.0034650	4.4603070	-3.0684280
O	4.9359620	3.8351650	-3.5143160
O	3.3498320	5.4057660	-3.7689030
H	4.3917910	-4.5599170	-2.1561900
H	5.5290390	-5.1460270	-0.9656660
H	6.7151000	-3.7539980	-2.5994120
H	5.5574420	-2.4490320	-2.4016300
H	7.5557970	-3.4299210	-0.3210650
H	3.7974990	-1.4758690	1.6154250
H	4.7669110	-2.1563550	2.9133350
H	2.3804180	-2.7205890	3.1752890
H	3.3262120	-4.1733140	2.9858540
H	1.3191210	-4.3558350	1.6238530
H	6.7201590	-1.6818030	2.4751890
H	8.0429820	-1.6041000	1.3285280
H	7.1704630	3.3928750	2.0635660
H	3.2149250	1.5169320	-0.8316640
H	4.4684620	1.6732050	-2.0749810
H	1.0194780	-2.0597210	1.3734440
H	2.2697600	-6.4203460	0.7796580
H	3.8165080	-6.4109160	-0.0623350
H	3.7254770	-5.9033890	1.6214810
H	7.3841790	-0.7998590	-1.8812530
H	8.6360270	-1.2041720	-0.6969680
H	8.5075290	-2.1241020	-2.1911820
H	2.4091230	-5.2915590	-1.7875790
H	1.8343300	-3.6295040	-1.5761010
H	1.0126410	-4.9657560	-0.7649900
H	6.6970170	-3.7986470	2.2709900
H	6.3751560	-4.8967110	0.9359530
H	5.2800080	-4.8195060	2.3041120
H	8.4549350	0.2318500	3.0239240
H	-2.5059780	1.5518390	-0.0527440

H	-2.4648330	1.3768840	1.7077930
H	-6.0247990	5.1968510	1.2955980
H	-8.2580890	0.8111910	-0.0817710
H	-8.3463000	0.6384070	1.6597180
H	-7.8168490	-1.7785950	2.0738000
H	-5.9981760	-3.2147850	2.8680210
H	-4.7987530	-2.1981590	2.0871890
H	-5.1476270	-4.1693840	0.7209850
H	-6.8817510	-3.9540890	0.7039150
H	-5.4695310	-2.9301510	-3.9847170
H	-6.3830440	-0.6116600	-3.9345910
H	-7.5175670	-1.7932790	-3.3367750
H	-5.7498340	0.0698300	-1.6692460
H	-7.4855960	0.2243660	-1.8944220
H	-4.0930700	-1.0730360	-3.6883250
H	-7.6855960	-3.8411290	-2.4365190
H	-6.9151160	-4.9991870	-1.3562830
H	-6.4559100	-4.9362760	-3.0552060
H	-4.0371210	-4.5566720	-2.7637240
H	-3.4602960	-3.5750100	-1.4137710
H	-4.4331280	-5.0243020	-1.1124990
H	-6.6699440	-1.2606440	4.2010730
H	-7.2753880	0.2232770	3.4748830
H	-5.5538050	-0.1746020	3.3722090
H	-8.6253550	-2.2899450	-1.5467050
H	-8.5014660	-2.7516660	0.1410680
H	-9.0549310	-1.1428170	-0.3016390
H	-8.8551820	3.0689750	1.1416660
H	3.5364760	5.2919910	-1.1838110
H	1.4211080	4.6870270	-2.3383930
H	1.7787160	2.9800590	-2.1624510
H	1.7703210	3.2580920	0.3518850
H	1.4144780	4.9645950	0.1481440
H	-0.3778270	2.6775780	-0.8285990
H	-0.7454860	4.3911910	-0.9874970
H	-0.3751590	2.9250250	1.6792930
H	-0.7503310	4.6408550	1.4997410
H	3.7543710	5.4722080	-4.6488630

Table S16. Coordinates (Ångstroms) for conformer (2′′*R*, 10*R*, 10′*R*, 13*R*, 13′*R*, 14*S*, 14′*S*, 15*S*, 15′*S*, 18*R*, 18′*R*)-**2**_C2 (Energy: -2885.28789 Hartree, Solvent: CH_3OH, Boltzmann %: 1.4).

Symbol	X	Y	Z
C	5.0698560	3.0748670	-0.9665490
C	4.1874890	3.5845050	0.2329480
C	5.0612680	4.2244590	1.3247820
C	6.1058810	3.2345220	1.8460590
C	6.9686220	2.6041370	0.7406720
C	6.0911770	2.0161850	-0.3936950
C	4.1479720	2.3964720	-2.0120320
C	2.9563690	3.2679250	-2.4244780
C	2.1020350	3.6837050	-1.2266750
C	2.9151140	4.4275260	-0.1365580
O	5.2998450	0.9113300	0.2131020
C	5.6852990	-0.2685910	-0.3241040
C	6.6526330	-0.1405380	-1.3196350
C	6.9538770	1.3218990	-1.5036650
C	5.1986370	-1.5146980	0.0381100
C	5.7106430	-2.6249600	-0.6349860
C	6.6758780	-2.5409830	-1.6323310
C	7.1498170	-1.2691160	-1.9747690
C	4.1812350	-1.9474130	1.0537380
N	4.1640100	-3.4012490	0.8652040
C	5.0537530	-3.8387270	-0.0871490
O	1.4916730	2.5217830	-0.6249920
C	3.2137980	5.8587800	-0.6370590
H	3.7736350	2.6703630	0.6700190
C	7.9426940	1.5902500	1.3611320
C	2.0194640	4.5792720	1.1161090
C	5.8855470	4.1931640	-1.6633540
O	5.2446380	-5.0123280	-0.3991220
O	8.0982680	-1.1893560	-2.9594940
N	-2.3490660	-3.4306480	-0.9812550
C	-2.8543550	-2.0607600	-0.8757080
C	-4.3409950	-2.2680750	-0.9073530
C	-4.6281820	-3.6293790	-1.0215530
C	-3.3382580	-4.3710140	-1.0677180
C	-5.4131620	-1.3922160	-0.8457060
C	-6.7249690	-1.8615100	-0.8968590
C	-6.9833070	-3.2288090	-1.0105500
C	-5.9208110	-4.1381580	-1.0767000
O	-5.3231410	-0.0468050	-0.7294530
C	-6.6868890	0.5471410	-0.7538730
C	-7.6559440	-0.6854790	-0.7778520
C	-6.7802440	1.3835400	-2.0557460
C	-5.7892630	2.5587130	-2.0387250
C	-5.8668910	3.4141970	-0.7717680
C	-5.7264180	2.5481870	0.4914180
C	-6.8414890	1.4390740	0.5395150

C	-5.4983990	3.3759190	1.8064570
C	-5.3520790	2.4079320	3.0082380
C	-6.4866240	1.3863110	3.0892500
C	-6.6225720	0.5659730	1.8017820
O	-4.0810230	1.7324510	2.8965680
H	-4.7939720	1.9870910	0.3744640
C	-6.6159730	4.3934650	2.1294840
C	-4.1887110	4.1897910	1.6784150
C	-6.5816650	0.5597570	-3.3378020
C	-8.2848330	2.0031600	0.5592840
O	-8.2559040	-3.7344480	-1.0594010
O	-3.1713190	-5.5879260	-1.1672460
C	3.3838580	-4.3122730	1.6785310
C	1.8809810	-3.9789200	1.7099170
C	1.2220860	-4.0341490	0.3286300
C	-0.2738890	-3.7069790	0.3867780
C	-0.9282900	-3.7451700	-1.0011500
C	3.9406030	-4.4813620	3.0994600
O	3.3239460	-4.9899760	4.0067930
O	5.2053400	-4.0459660	3.2276000
H	4.4387880	4.5461000	2.1628260
H	5.5582320	5.1257260	0.9525080
H	6.7659080	3.7295020	2.5662610
H	5.5981860	2.4303330	2.3910110
H	7.5661890	3.3965970	0.2749320
H	3.7636620	1.4632140	-1.5957850
H	4.7160570	2.1365370	-2.9099260
H	2.3292810	2.7151310	-3.1339150
H	3.2862800	4.1625240	-2.9603180
H	1.3021390	4.3560890	-1.5657780
H	6.6754540	1.6580600	-2.5044680
H	8.0138510	1.5572370	-1.3778080
H	7.0553210	-3.4224500	-2.1337040
H	3.1938570	-1.5128300	0.8676890
H	4.4758320	-1.6914330	2.0772690
H	0.9951080	2.0614380	-1.3104570
H	2.2767110	6.4150030	-0.7394050
H	3.8351950	6.3990480	0.0805000
H	3.7180870	5.8912940	-1.6015870
H	7.4034830	0.7687000	1.8396210
H	8.6387600	1.1647460	0.6352520
H	8.5403630	2.0857500	2.1310750
H	2.4531690	5.2845440	1.8283910
H	1.8641030	3.6265270	1.6242890
H	1.0382330	4.9690610	0.8280630
H	6.6661340	3.7698470	-2.3000270
H	6.3706600	4.8704620	-0.9611440
H	5.2537100	4.7973100	-2.3118190
H	8.3676570	-0.2734470	-3.0961480
H	-2.5165600	-1.5851530	0.0515320

H	-2.4924960	-1.4523690	-1.7129670
H	-6.1114090	-5.2003900	-1.1655240
H	-8.2562760	-0.7482710	0.1320420
H	-8.3587930	-0.6118770	-1.6117830
H	-7.7982470	1.7902360	-2.0766970
H	-5.9674320	3.1801740	-2.9227360
H	-4.7740870	2.1579870	-2.1399860
H	-5.0692480	4.1597730	-0.8063720
H	-6.8064560	3.9753650	-0.7609630
H	-5.3409410	3.0122990	3.9256200
H	-6.2984700	0.7115150	3.9326620
H	-7.4201440	1.9029030	3.3300150
H	-5.7136220	-0.0248860	1.6723890
H	-7.4486790	-0.1403960	1.9249310
H	-4.0038580	1.1246670	3.6402360
H	-7.5672680	3.9335110	2.3926040
H	-6.7923640	5.0591460	1.2817130
H	-6.3104470	5.0177340	2.9750140
H	-3.9027400	4.5926110	2.6550060
H	-3.3622610	3.5778440	1.3145440
H	-4.3154520	5.0376030	1.0016360
H	-6.6890920	1.2120350	-4.2087780
H	-7.3093380	-0.2472030	-3.4460490
H	-5.5801690	0.1234130	-3.3735390
H	-8.5484500	2.3797200	1.5459910
H	-8.4366240	2.8170890	-0.1491220
H	-9.0133810	1.2244090	0.3214890
H	-8.9034020	-3.0211140	-1.0165080
H	3.5144280	-5.2973650	1.2148330
H	1.4020840	-4.6961160	2.3796410
H	1.7329000	-2.9905690	2.1585620
H	1.7209030	-3.3345900	-0.3512750
H	1.3624380	-5.0342620	-0.0988260
H	-0.4127700	-2.7144810	0.8295790
H	-0.7872210	-4.4206790	1.0401250
H	-0.4288330	-3.0394500	-1.6732990
H	-0.8296090	-4.7422950	-1.4348590
H	5.4958640	-4.2227260	4.1371150

Table S17. Coordinates (Ångstroms) for conformer (2′′*R*, 10*R*, 10′*R*, 13*R*, 13′*R*, 14*S*, 14′*S*, 15*S*, 15′*S*, 18*R*, 18′*R*)-**2**_C3 (Energy: -2885.29129 Hartree, Solvent: CH_3OH, Boltzmann %: 50.2).

Symbol	X	Y	Z
C	7.2709380	-2.4711360	-0.4232750
C	6.8039380	-2.3794050	-1.9232420
C	7.9435710	-1.8744390	-2.8237580
C	8.4424270	-0.5025550	-2.3636330
C	8.8421390	-0.4540100	-0.8798760
C	7.7226850	-1.0269640	0.0265670
C	6.0738080	-2.9351740	0.4455240
C	5.3918370	-4.2000230	-0.0879540
C	4.9071770	-4.0338260	-1.5285420
C	6.0420980	-3.6245790	-2.5018990
O	6.5501060	-0.1227470	-0.1248180
C	6.3187820	0.5162910	1.0443130
C	7.1743550	0.1273560	2.0737770
C	8.0975230	-0.9373470	1.5464060
C	5.3369550	1.4707810	1.2583880
C	5.2439220	2.0219660	2.5371470
C	6.0790720	1.6645820	3.5899770
C	7.0592510	0.6958590	3.3442260
C	4.2937090	2.0655580	0.3582360
N	3.6152410	3.0035290	1.2611020
C	4.1346890	3.0095070	2.5364130
O	3.8697200	-3.0316950	-1.5925100
C	6.9327350	-4.8597180	-2.7655080
H	6.0449840	-1.5909140	-1.9207700
C	9.2726640	0.9735430	-0.5076790
C	5.4028300	-3.2477960	-3.8597490
C	8.4633110	-3.4345670	-0.1964010
O	3.7340170	3.7020650	3.4683510
O	7.8758580	0.3420060	4.3850120
N	-3.5585210	3.1413190	-1.0331170
C	-4.3871830	2.2826800	-0.1851180
C	-5.3935790	1.7485070	-1.1629150
C	-5.1403540	2.2648810	-2.4350090
C	-3.9553720	3.1610390	-2.3416020
C	-6.4680610	0.8838920	-1.0270050
C	-7.2586190	0.5467670	-2.1247130
C	-6.9804560	1.0750890	-3.3866380
C	-5.9042290	1.9546850	-3.5545640
O	-6.8587890	0.2900210	0.1248240
C	-8.0881090	-0.5112130	-0.1212390
C	-8.3161560	-0.4216780	-1.6698510
C	-9.2293960	0.1759550	0.6718240
C	-8.9691420	0.1249640	2.1861140
C	-8.6368290	-1.2747680	2.7085250
C	-7.4717850	-1.8980140	1.9215510
C	-7.8096950	-1.9796480	0.3867940

C	-6.8804440	-3.1936120	2.5834170
C	-5.7069380	-3.7256840	1.7216690
C	-6.0755940	-3.8770820	0.2457080
C	-6.5860500	-2.5663380	-0.3628460
O	-4.5899110	-2.8221840	1.8650810
H	-6.6477580	-1.1798360	1.9771700
C	-7.9003990	-4.3373730	2.7837740
C	-6.3312140	-2.8455720	3.9875230
C	-9.4972200	1.6271910	0.2428480
C	-9.0582070	-2.8395580	0.0652680
O	-7.7270330	0.7654950	-4.4932320
O	-3.4233730	3.8054360	-3.2473640
C	2.4876070	3.8418050	0.8680280
C	1.2996080	3.0358050	0.3289110
C	0.0266280	3.8756020	0.1678100
C	-1.1510890	3.0505450	-0.3620750
C	-2.4232950	3.8945970	-0.5212960
C	2.9988940	4.8771850	-0.1440020
O	2.9688780	4.7486760	-1.3456480
O	3.5371550	5.9466960	0.4683940
H	7.5967790	-1.7897370	-3.8561120
H	8.7757560	-2.5850750	-2.8404900
H	9.3014750	-0.1927930	-2.9681180
H	7.6563230	0.2415780	-2.5360150
H	9.7082160	-1.1103480	-0.7340230
H	5.3320410	-2.1352080	0.4857720
H	6.3984710	-3.1224000	1.4732030
H	4.5348780	-4.4478560	0.5494440
H	6.0595760	-5.0643040	-0.0299060
H	4.4885120	-4.9856490	-1.8831670
H	7.9369440	-1.8865090	2.0618740
H	9.1539820	-0.6867710	1.6727040
H	5.9827290	2.1126050	4.5709100
H	3.5950510	1.3150680	-0.0247360
H	4.7264560	2.5887810	-0.5012400
H	3.1759130	-3.2854330	-0.9739380
H	6.3541920	-5.6230720	-3.2951640
H	7.7819010	-4.5975350	-3.4004240
H	7.3259500	-5.3202150	-1.8604990
H	8.4460650	1.6781640	-0.6295880
H	9.6437960	1.0564550	0.5159680
H	10.0800040	1.2936780	-1.1719470
H	6.1585670	-3.1728750	-4.6446430
H	4.8635070	-2.3009440	-3.8078700
H	4.6920030	-4.0219940	-4.1649060
H	8.9182150	-3.2709460	0.7834450
H	9.2501480	-3.3254620	-0.9421840
H	8.1409040	-4.4740290	-0.2159550
H	8.5181450	-0.3192390	4.1016230
H	-3.7897780	1.4894750	0.2777480

H	-4.8465220	2.8668420	0.6208980
H	-5.6820580	2.3712100	-4.5290990
H	-8.1971970	-1.3922030	-2.1558400
H	-9.3303100	-0.0813870	-1.8942520
H	-10.1337810	-0.4055830	0.4570800
H	-9.8476660	0.5211980	2.7062800
H	-8.1387570	0.8016530	2.4184410
H	-8.3762210	-1.1980600	3.7665990
H	-9.5273320	-1.9092220	2.6607830
H	-5.4117970	-4.7040600	2.1249980
H	-5.1925950	-4.2165770	-0.3085120
H	-6.8139720	-4.6767960	0.1389020
H	-5.7735190	-1.8373880	-0.3430120
H	-6.8316560	-2.7433050	-1.4139830
H	-3.8723860	-3.1506750	1.3121900
H	-8.2553810	-4.7787690	1.8537710
H	-8.7731830	-3.9863030	3.3386850
H	-7.4421550	-5.1389470	3.3715480
H	-5.7237700	-3.6733480	4.3664270
H	-5.7053570	-1.9522040	3.9703250
H	-7.1430490	-2.6851940	4.7003070
H	-10.3288790	2.0305840	0.8270060
H	-9.7670890	1.7198620	-0.8111970
H	-8.6255730	2.2597740	0.4290220
H	-8.8318520	-3.9026010	0.1253980
H	-9.8949510	-2.6480570	0.7363850
H	-9.4088060	-2.6559330	-0.9531380
H	-8.4384840	0.1574500	-4.2603900
H	2.1985270	4.3760530	1.7750060
H	1.5664070	2.5836710	-0.6305240
H	1.1141960	2.2190770	1.0333990
H	-0.2416760	4.3164210	1.1355010
H	0.2156330	4.7111850	-0.5156470
H	-1.3476080	2.2148760	0.3186120
H	-0.8921790	2.6158710	-1.3334580
H	-2.7086720	4.3350040	0.4395520
H	-2.2423190	4.7136390	-1.2204320
H	3.8955430	6.5441900	-0.2085280

Table S18. Coordinates (Ångstroms) for conformer (2′′*R*, 10*R*, 10′*R*, 13*R*, 13′*R*, 14*S*, 14′*S*, 15*S*, 15′*S*, 18*R*, 18′*R*)-**2**_C4 (Energy: -2885.28879 Hartree, Solvent: CH_3OH, Boltzmann %: 3.6).

Symbol	X	Y	Z
C	8.7274890	1.6344310	-0.5874560
C	8.8893730	1.7613880	0.9728830
C	10.0239640	0.8569850	1.4826670
C	9.7679890	-0.6069930	1.1165780
C	9.5042960	-0.8350020	-0.3808640
C	8.4088740	0.1234450	-0.9142040
C	7.5429300	2.5258630	-1.0409250
C	7.6383800	3.9665640	-0.5270500
C	7.7326340	4.0322050	0.9974820
C	8.9235830	3.2165670	1.5624020
O	7.1510510	-0.2340610	-0.2035770
C	6.2634860	-0.7458100	-1.0863010
C	6.7199910	-0.7452300	-2.4034880
C	8.0937330	-0.1319950	-2.4287090
C	5.0058140	-1.2346390	-0.7696420
C	4.2226700	-1.7208940	-1.8173590
C	4.6402810	-1.7395850	-3.1435830
C	5.9149800	-1.2385200	-3.4327040
C	4.2682090	-1.3564670	0.5317580
N	2.9992620	-1.9591270	0.1043810
C	2.9319460	-2.1894020	-1.2520030
O	6.5158560	3.5331980	1.5932280
C	10.2288630	3.9946990	1.2810020
H	7.9616330	1.3416000	1.3741330
C	9.2043710	-2.3212470	-0.6315520
C	8.7766590	3.1483710	3.1011890
C	9.9948040	2.0360410	-1.3836520
O	1.9769510	-2.6821980	-1.8467640
O	6.3203330	-1.2550850	-4.7406590
N	-3.5923350	-1.9222960	1.9160930
C	-4.3173540	-0.8236510	1.2757920
C	-5.4922700	-1.5324290	0.6664290
C	-5.4193640	-2.8984950	0.9443980
C	-4.1915520	-3.1404350	1.7504780
C	-6.5717240	-1.0896930	-0.0811840
C	-7.5450640	-1.9808420	-0.5313350
C	-7.4484550	-3.3422240	-0.2364430
C	-6.3664480	-3.8201400	0.5127760
O	-6.8040620	0.1942230	-0.4411940
C	-8.0244050	0.2596080	-1.2891070
C	-8.5933170	-1.2014770	-1.2782230
C	-7.5567220	0.6878210	-2.7038990
C	-6.9565540	2.1031330	-2.6909190
C	-7.8511600	3.1463870	-2.0172730
C	-8.2526640	2.7000700	-0.6014660
C	-9.0008640	1.3160890	-0.6389690

C	-8.9137470	3.8358750	0.2587420
C	-9.2584300	3.2845650	1.6658760
C	-10.0557370	1.9811100	1.6136830
C	-9.3344400	0.8905900	0.8139600
O	-8.0257060	3.0856870	2.3908300
H	-7.3183500	2.4864040	-0.0729090
C	-10.1924240	4.4480280	-0.3562470
C	-7.9084020	4.9997060	0.4283540
C	-6.5622500	-0.2887900	-3.3511150
C	-10.3067760	1.3325780	-1.4730560
O	-8.3826810	-4.2541270	-0.6531220
O	-3.7805590	-4.2134120	2.1958290
C	1.9057150	-2.2942590	1.0110210
C	1.4235360	-1.0907040	1.8331070
C	0.1427440	-1.3483300	2.6424610
C	-1.1182420	-1.5614810	1.7952560
C	-2.3672230	-1.7197070	2.6741410
C	2.3709940	-3.4544170	1.9026430
O	2.9384140	-3.3298080	2.9631050
O	2.1145040	-4.6488220	1.3410140
H	10.1060190	0.9342060	2.5692050
H	10.9912290	1.1758440	1.0819180
H	10.6197570	-1.2247400	1.4202320
H	8.9021620	-0.9721540	1.6809350
H	10.4161930	-0.5823780	-0.9346690
H	6.6101720	2.0915530	-0.6758770
H	7.4794790	2.5494830	-2.1327030
H	6.7557380	4.5290070	-0.8536550
H	8.4955610	4.4867560	-0.9639090
H	7.8646330	5.0774330	1.3090130
H	8.1006680	0.7941270	-3.0069470
H	8.8447790	-0.7870920	-2.8773040
H	4.0095740	-2.1260500	-3.9341820
H	4.1007960	-0.3860580	1.0093500
H	4.7862490	-1.9986810	1.2519190
H	5.7803980	4.0366800	1.2272080
H	10.2235480	4.9351110	1.8409660
H	11.0996950	3.4246040	1.6120480
H	10.3753640	4.2428570	0.2308500
H	8.2999980	-2.6345050	-0.1037120
H	9.0824500	-2.5616100	-1.6897480
H	10.0330050	-2.9271090	-0.2549370
H	9.6945660	2.7869350	3.5698100
H	7.9548440	2.4976030	3.4031560
H	8.5776130	4.1472560	3.5016170
H	9.9315820	1.6927670	-2.4190390
H	10.9129650	1.6284530	-0.9616950
H	10.1098640	3.1176810	-1.4247140
H	7.2134090	-0.9006970	-4.8232570
H	-4.6120020	-0.0735070	2.0189550

H	-3.6946240	-0.3228910	0.5262580
H	-6.2813470	-4.8746580	0.7437320
H	-9.5600080	-1.2570790	-0.7737460
H	-8.7499540	-1.5651070	-2.2969610
H	-8.4600060	0.7016340	-3.3252610
H	-6.7416830	2.4021860	-3.7223220
H	-5.9926170	2.0666460	-2.1703870
H	-7.3080430	4.0931430	-1.9724490
H	-8.7344340	3.3323640	-2.6362800
H	-9.8465810	4.0497730	2.1908090
H	-10.2284630	1.6280700	2.6372680
H	-11.0487070	2.1825840	1.2017690
H	-8.4072640	0.6392060	1.3327170
H	-9.9556880	-0.0097120	0.8147940
H	-8.2477270	2.7307940	3.2586580
H	-11.0371790	3.7617130	-0.3886530
H	-10.0059860	4.7934400	-1.3754960
H	-10.5016280	5.3185300	0.2308090
H	-8.2642900	5.6908630	1.1986980
H	-6.9214460	4.6446150	0.7282570
H	-7.8046160	5.5699940	-0.4972760
H	-6.3047570	0.0662650	-4.3527280
H	-6.9621780	-1.2991860	-3.4586630
H	-5.6361610	-0.3477990	-2.7736220
H	-11.1154350	1.8211010	-0.9324760
H	-10.1989190	1.8461810	-2.4279920
H	-10.6477760	0.3167320	-1.6864090
H	-9.0774650	-3.8191620	-1.1611530
H	1.1097570	-2.6665940	0.3659420
H	2.2189680	-0.7835100	2.5166360
H	1.2586210	-0.2634340	1.1356050
H	0.2894320	-2.2062810	3.3089180
H	-0.0137220	-0.4846850	3.2978240
H	-1.0233330	-2.4534490	1.1681470
H	-1.2502530	-0.7095860	1.1189640
H	-2.2535670	-2.5839160	3.3318070
H	-2.4967120	-0.8354020	3.3068870
H	2.4745620	-5.3455380	1.9144020

Table S19. Coordinates (Ångstroms) for conformer (2''*S*, 3*R*, 5*S*, 8*R*, 9*R*, 10*S*)-**3**_C1 (Energy: -1748.9471 Hartree, Solvent: CH_3OH, Boltzmann %: 73.9).

Symbol	X	Y	Z
C	3.7146910	-0.5053060	-0.1554090
C	3.8393740	0.4955010	1.0526090
C	3.9998270	-0.2632060	2.3806480
C	2.8192520	-1.2069140	2.6223380
C	2.5509560	-2.1744520	1.4581390
C	2.4555750	-1.4202980	0.1069710
C	3.5034070	0.3086100	-1.4575910
C	4.5453500	1.4150250	-1.6554200
C	4.5847660	2.3912630	-0.4793370
C	4.8452520	1.6889940	0.8779430
O	1.2655220	-0.5316570	0.2007550
C	0.3171980	-0.9541920	-0.6666060
C	0.7163090	-2.0377720	-1.4474240
C	2.1327840	-2.3894660	-1.0823440
C	-0.9450400	-0.4001020	-0.8112210
C	-1.7928210	-0.9730480	-1.7601500
C	-1.4343560	-2.0539930	-2.5582230
C	-0.1515110	-2.5886550	-2.3929150
C	-1.6332640	0.7407500	-0.1199470
N	-2.9530660	0.7311260	-0.7614030
C	-3.0772650	-0.2292760	-1.7388560
O	3.3362090	3.1100830	-0.3874390
H	2.8616030	0.9854390	1.0974840
C	1.3194150	-3.0367340	1.7765900
C	6.3364620	1.2890100	0.9457440
C	4.9452420	-1.4306730	-0.3321640
O	0.2000790	-3.6499440	-3.1841600
C	4.6165960	2.7206190	2.0083880
C	-3.9962320	1.6970160	-0.4998710
C	-4.3983660	1.7905470	0.9963070
C	-3.6376670	3.0689180	-1.0833740
O	-2.6137040	3.3384340	-1.6638820
O	-4.6317120	3.9554830	-0.8928760
O	-4.0704490	-0.4080110	-2.4404770
H	4.0560700	0.4431190	3.2120510
H	4.9364590	-0.8290910	2.3995120
H	2.9864170	-1.7922080	3.5327600
H	1.9144280	-0.6130880	2.7961480
H	3.4113480	-2.8474560	1.3635390
H	3.5285200	-0.3523460	-2.3288030
H	2.5131060	0.7676360	-1.4336430
H	4.3161990	1.9712900	-2.5719880
H	5.5436630	0.9956180	-1.8091670
H	5.3907440	3.1205600	-0.6392390
H	2.8070260	-2.2353180	-1.9273010
H	2.2471820	-3.4324620	-0.7761770

H	-2.1159790	-2.4759050	-3.2859830
H	-1.7165750	0.5802320	0.9592500
H	-1.1316230	1.6987540	-0.2854870
H	3.1726340	3.5275220	-1.2403350
H	0.4233880	-2.4197910	1.8818180
H	1.1220320	-3.7985710	1.0195760
H	1.4760190	-3.5561770	2.7259210
H	6.9578290	2.1899360	0.9586700
H	6.5486640	0.7350890	1.8628370
H	6.6668960	0.6787600	0.1066270
H	4.7145250	-2.2655920	-0.9980470
H	5.3023430	-1.8530830	0.6066490
H	5.7791890	-0.8976020	-0.7851180
H	1.0968090	-3.9411480	-2.9814130
H	5.0016640	2.3530070	2.9620230
H	3.5600470	2.9623610	2.1323310
H	5.1481040	3.6493950	1.7788920
H	-4.8649990	1.3552210	-1.0705930
H	-4.3719480	4.8009480	-1.2932720
H	-5.1546660	2.5731650	1.0805080
H	-3.5402150	2.1186250	1.5880780
C	-4.9431780	0.4898640	1.5489300
C	-6.1756350	-0.0103250	1.1079940
C	-4.2411740	-0.2316920	2.5194430
C	-6.6866060	-1.2023370	1.6168950
H	-6.7456840	0.5388280	0.3651550
C	-4.7497310	-1.4260010	3.0332080
H	-3.2921330	0.1450640	2.8865800
C	-5.9732040	-1.9165210	2.5813060
H	-7.6432390	-1.5716520	1.2643650
H	-4.1897430	-1.9692900	3.7862210
H	-6.3706230	-2.8437050	2.9784330

Table S20. Coordinates (Ångstroms) for conformer (2''*S*, 3*R*, 5*S*, 8*R*, 9*R*, 10*S*)-**3**_C2 (Energy: -1748.9460 Hartree, Solvent: CH_3OH, Boltzmann %: 24.3).

Symbol	X	Y	Z
C	-4.0483540	-0.0097130	-0.0270610
C	-3.8763860	-1.4601650	0.5586650
C	-4.2154640	-1.4911240	2.0583660
C	-3.3333230	-0.5176430	2.8437360
C	-3.3562070	0.9191090	2.2971560
C	-3.0802250	0.9472260	0.7720260
C	-3.6452030	-0.0243500	-1.5239850
C	-4.3459990	-1.1241140	-2.3292750
C	-4.0968570	-2.5162720	-1.7488390
C	-4.5211220	-2.6331580	-0.2625570
O	-1.6929880	0.4418100	0.5832520
C	-0.9078750	1.4401880	0.1191270
C	-1.5952590	2.6301680	-0.1150950
C	-3.0479830	2.4103400	0.2091720
C	0.4536000	1.3458900	-0.1235020
C	1.1005670	2.4847690	-0.6049650
C	0.4531910	3.6908820	-0.8496210
C	-0.9224110	3.7542920	-0.5991950
C	1.4317540	0.2185990	0.0355630
N	2.6850030	0.8417870	-0.4097620
C	2.5369550	2.1590070	-0.7890310
O	-2.6970620	-2.8548570	-1.8494350
H	-2.8017980	-1.6537000	0.4843860
C	-2.3999740	1.7989110	3.1176570
C	-6.0636430	-2.6955330	-0.1946160
C	-5.4867720	0.5520970	0.1030000
O	-1.5576510	4.9416200	-0.8476250
C	-4.0030090	-3.9852800	0.2827210
C	3.9749660	0.1649510	-0.4420860
C	4.3904530	-0.3986060	0.9336110
C	3.9199800	-0.9330580	-1.5119540
O	3.4016120	-2.0147580	-1.3599620
O	4.4831690	-0.5396230	-2.6666280
O	3.4363880	2.8916400	-1.1916360
H	-4.0629400	-2.4967160	2.4568020
H	-5.2704840	-1.2544510	2.2281190
H	-3.6398810	-0.4974670	3.8949120
H	-2.2984090	-0.8783230	2.8284760
H	-4.3684630	1.3208740	2.4240390
H	-3.8671680	0.9410710	-1.9880020
H	-2.5666020	-0.1749520	-1.6007430
H	-3.9875090	-1.1012810	-3.3651650
H	-5.4235070	-0.9458410	-2.3878560
H	-4.6721570	-3.2568960	-2.3211570
H	-3.6704570	2.5166980	-0.6815610
H	-3.4326010	3.1193550	0.9467410

H	0.9846900	4.5571360	-1.2228110
H	1.5099450	-0.1217360	1.0731370
H	1.1835330	-0.6492300	-0.5832880
H	-2.4353490	-2.7562400	-2.7714900
H	-1.3686040	1.4483170	3.0282380
H	-2.4293020	2.8511390	2.8271160
H	-2.6758440	1.7468770	4.1744120
H	-6.4154040	-3.6144570	-0.6740090
H	-6.4059070	-2.7189530	0.8423430
H	-6.5598370	-1.8613230	-0.6881480
H	-5.5021470	1.6319490	-0.0629300
H	-5.9322730	0.3673120	1.0801010
H	-6.1499340	0.1171560	-0.6427020
H	-2.4948900	4.8760830	-0.6303800
H	-4.4576740	-4.2229810	1.2468780
H	-2.9185850	-3.9878120	0.4014300
H	-4.2660390	-4.7915420	-0.4091060
H	4.6908290	0.9203550	-0.7653350
H	4.3853570	-1.2534750	-3.3186860
H	4.2676740	0.4105930	1.6586380
H	3.7100940	-1.2037920	1.2175880
C	5.8195210	-0.9007730	0.9547200
C	6.1050230	-2.2696500	0.9003050
C	6.8886890	0.0028890	1.0207740
C	7.4239350	-2.7259220	0.9086650
H	5.2898090	-2.9837120	0.8545500
C	8.2069200	-0.4490910	1.0287640
H	6.6885030	1.0684420	1.0747850
C	8.4789650	-1.8169150	0.9713580
H	7.6250130	-3.7907480	0.8690360
H	9.0208140	0.2652400	1.0846950
H	9.5040210	-2.1698690	0.9799280

Table S21. Coordinates (Ångstroms) for conformer (2′′*S*, 3*R*, 5*S*, 8*R*, 9*R*, 10*S*)-**3**_C3 (Energy: -1748.9433 Hartree, Solvent: CH_3OH, Boltzmann %: 1.3).

Symbol	X	Y	Z
C	-3.5084600	0.2840160	-0.0695220
C	-3.3849610	-1.2708990	0.1395920
C	-3.6627560	-1.6525110	1.6031260
C	-2.7004380	-0.9346690	2.5524020
C	-2.6735840	0.5916200	2.3703360
C	-2.4607040	0.9797000	0.8845800
C	-3.1699530	0.6173560	-1.5451310
C	-3.9609330	-0.2244120	-2.5524530
C	-3.7568500	-1.7246000	-2.3399580
C	-4.1211490	-2.1837490	-0.9049670
O	-1.1101940	0.4830660	0.5063480
C	-0.2969570	1.5340480	0.2525820
C	-0.9337150	2.7699760	0.3489980
C	-2.3796080	2.5336710	0.6913160
C	1.0460250	1.4492140	-0.0810220
C	1.7268090	2.6455710	-0.3081210
C	1.1300390	3.8992420	-0.2207450
C	-0.2280410	3.9519300	0.1119180
C	1.9706010	0.2806020	-0.2547170
N	3.2401530	0.9456770	-0.5883060
C	3.1329850	2.3170670	-0.6464000
O	-2.3816450	-2.0813880	-2.5959800
H	-2.3252900	-1.4818190	-0.0350280
C	-1.6402110	1.2072900	3.3267230
C	-5.6606530	-2.2036400	-0.7714630
C	-4.9103610	0.8519690	0.2678250
O	-0.8152780	5.1871410	0.1907680
C	-3.6468030	-3.6461280	-0.7298360
C	4.4849860	0.2827500	-0.9190890
C	5.2213960	-0.3735940	0.2850820
C	4.3350290	-0.6158450	-2.1477980
O	3.3430030	-0.7206490	-2.8284570
O	5.4871010	-1.2534170	-2.4297700
O	4.0461680	3.0911070	-0.9290490
H	-3.5451590	-2.7300660	1.7385930
H	-4.6964740	-1.4232490	1.8801360
H	-2.9611800	-1.1590250	3.5921470
H	-1.6866680	-1.3206170	2.3955380
H	-3.6578970	0.9895470	2.6441170
H	-3.3594460	1.6749730	-1.7503720
H	-2.1052340	0.4448940	-1.7137970
H	-3.6481700	0.0367290	-3.5703230
H	-5.0295430	0.0040880	-2.5067180
H	-4.3933200	-2.2810510	-3.0416510
H	-3.0357360	2.8782950	-0.1106180
H	-2.6929540	3.0555770	1.5992570

H	1.6871080	4.8095390	-0.4033040
H	2.0665610	-0.3146400	0.6562410
H	1.6627970	-0.3825180	-1.0669840
H	-2.1560930	-1.7728370	-3.4804830
H	-0.6328690	0.8482850	3.1010480
H	-1.6276570	2.2988740	3.2995110
H	-1.8740620	0.9113820	4.3530450
H	-6.0769650	-2.9661970	-1.4371840
H	-5.9588960	-2.4636360	0.2465690
H	-6.1365560	-1.2573830	-1.0242790
H	-4.8797020	1.9399590	0.3639830
H	-5.3218580	0.4556580	1.1957300
H	-5.6260030	0.6333640	-0.5227500
H	-1.7445210	5.1047600	0.4352210
H	-4.0743050	-4.0949380	0.1694190
H	-2.5599690	-3.7167740	-0.6679920
H	-3.9741870	-4.2492780	-1.5823390
H	5.1448640	1.0899520	-1.2580960
H	5.3592860	-1.7706380	-3.2413430
H	6.1867330	-0.7245370	-0.0865270
H	5.4287050	0.4343700	0.9897310
C	4.5120820	-1.4975110	1.0134460
C	4.3961120	-2.7773320	0.4537830
C	3.9800540	-1.2819560	2.2909040
C	3.7478080	-3.8024380	1.1416680
H	4.8260540	-2.9813440	-0.5203630
C	3.3343910	-2.3059300	2.9838910
H	4.0731810	-0.3021770	2.7482860
C	3.2113690	-3.5699240	2.4083910
H	3.6691030	-4.7854380	0.6906530
H	2.9303840	-2.1158110	3.9720340
H	2.7103170	-4.3681950	2.9441130

Table S22. Coordinates (Ångstroms) for conformer (2′′*S*, 3*R*, 5*S*, 8*R*, 9*R*, 10*S*)-**3**_C4 (Energy: -1748.9424 Hartree, Solvent: CH_3OH, Boltzmann %: 0.5).

Symbol	X	Y	Z
C	-4.0597080	-0.0085140	0.0003780
C	-3.8886790	-1.4669050	0.5662100
C	-4.2172770	-1.5156740	2.0677970
C	-3.3253520	-0.5558240	2.8589560
C	-3.3455760	0.8879230	2.3307980
C	-3.0815770	0.9345290	0.8040250
C	-3.6680670	-0.0049200	-1.4997350
C	-4.3776180	-1.0925360	-2.3138030
C	-4.1281150	-2.4925820	-1.7527630
C	-4.5429420	-2.6272150	-0.2653420
O	-1.6980940	0.4261630	0.5976770
C	-0.9139010	1.4255930	0.1337810
C	-1.5984800	2.6208950	-0.0802440
C	-3.0482280	2.4045700	0.2593030
C	0.4435730	1.3267210	-0.1280610
C	1.0901720	2.4672440	-0.6067070
C	0.4454370	3.6785430	-0.8317740
C	-0.9264300	3.7462470	-0.5625150
C	1.4184400	0.1932400	0.0060660
N	2.6708270	0.8205700	-0.4301960
C	2.5213490	2.1327600	-0.8144770
O	-2.7299130	-2.8333280	-1.8663850
H	-2.8153660	-1.6632520	0.4819860
C	-2.3783450	1.7524240	3.1546690
C	-6.0851080	-2.6856690	-0.1875720
C	-5.4950640	0.5565170	0.1485700
O	-1.5592450	4.9389910	-0.7912080
C	-4.0254660	-3.9878590	0.2590230
C	3.9319790	0.1253480	-0.5456120
C	4.3443220	-0.5496900	0.7954530
C	3.9151160	-0.8287190	-1.7584380
O	2.9934970	-0.9169150	-2.5302990
O	5.0250310	-1.5640530	-1.9597150
O	3.4193270	2.8565560	-1.2393330
H	-4.0660260	-2.5268440	2.4523970
H	-5.2700820	-1.2769510	2.2481560
H	-3.6242450	-0.5475850	3.9125090
H	-2.2921870	-0.9208690	2.8317450
H	-4.3547960	1.2928850	2.4710620
H	-3.8912060	0.9669330	-1.9495330
H	-2.5905010	-0.1569670	-1.5868190
H	-4.0266240	-1.0574130	-3.3519250
H	-5.4550480	-0.9108240	-2.3623110
H	-4.7090060	-3.2244400	-2.3307330
H	-3.6811740	2.5254570	-0.6221200
H	-3.4197530	3.1063600	1.0103630

H	0.9760100	4.5456620	-1.2043320
H	1.4969840	-0.1680200	1.0366210
H	1.1685370	-0.6563080	-0.6365420
H	-2.4714320	-2.7169740	-2.7872790
H	-1.3495310	1.3978710	3.0521620
H	-2.4047200	2.8084270	2.8778380
H	-2.6457990	1.6882150	4.2129270
H	-6.4431400	-3.5972260	-0.6763000
H	-6.4202670	-2.7213560	0.8513680
H	-6.5819910	-1.8436110	-0.6668710
H	-5.5081130	1.6382360	-0.0048580
H	-5.9333260	0.3620440	1.1270570
H	-6.1656530	0.1323330	-0.5967170
H	-2.4943660	4.8747020	-0.5646440
H	-4.4742480	-4.2364680	1.2231940
H	-2.9402420	-3.9953790	0.3701230
H	-4.2958750	-4.7843620	-0.4412190
H	4.6675070	0.8993690	-0.7882080
H	5.6762160	-1.4414500	-1.2479190
H	4.1173340	0.1788990	1.5781960
H	3.7159440	-1.4236550	0.9808840
C	5.8061060	-0.9433370	0.8970210
C	6.8156520	0.0319850	0.8680130
C	6.1786400	-2.2847360	1.0579000
C	8.1557790	-0.3262690	0.9898680
H	6.5521140	1.0791560	0.7603820
C	7.5219410	-2.6449270	1.1804600
H	5.4119930	-3.0517610	1.0908710
C	8.5132200	-1.6669540	1.1455950
H	8.9210660	0.4411480	0.9679230
H	7.7889430	-3.6881350	1.3044880
H	9.5566960	-1.9444060	1.2404940

A) Structures of the low-energy conformers of (2′′*S*, 3*R*, 5*S*, 8*R*, 9*R*, 10*S*)-**1**

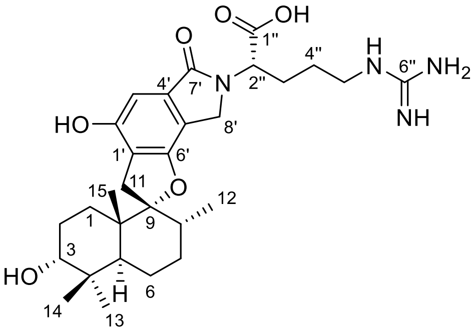

(2′′*S*, 3*R*, 5*S*, 8*R*, 9*R*, 10*S*)-**1**_C1 (58.3%)	(2′′*S*, 3*R*, 5*S*, 8*R*, 9*R*, 10*S*)-**1**_C2 (14.6%)
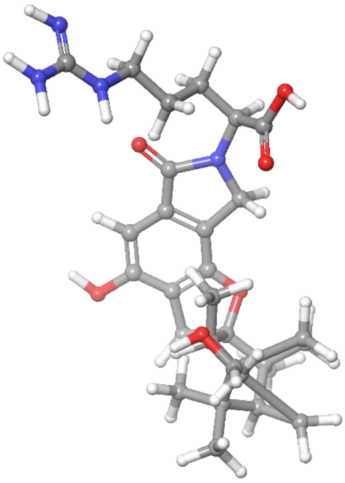	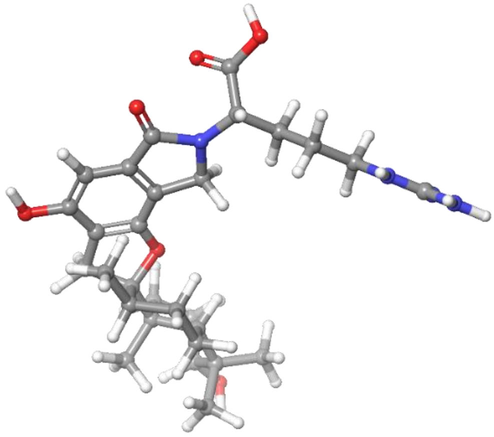
(2′′*S*, 3*R*, 5*S*, 8*R*, 9*R*, 10*S*)-**1**_C3 (27.1%)	(2′′*S*, 3*R*, 5*S*, 8*R*, 9*R*, 10*S*)-**1**_C4 ($1.9.10^{-2}$%)
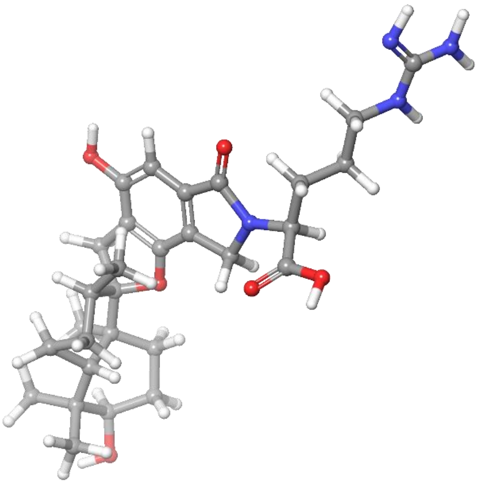	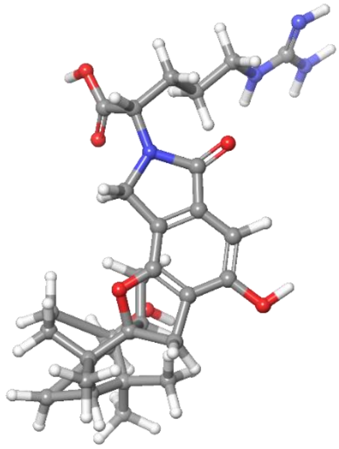

B) Structures of the low-energy conformers of (2′′*R*, 10*R*, 10′*R*, 13*R*, 13′*R*, 14*S*, 14′*S*, 15*S*, 15′*S*, 18*R*, 18′*R*)-**2**

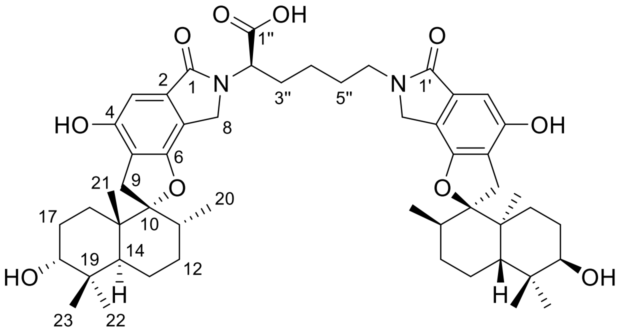

(2′′*R*, 10*R*, 10′*R*, 13*R*, 13′*R*, 14*S*, 14′*S*, 15*S*, 15′*S*, 18*R*, 18′*R*)-**2**_C1 (44.8%) 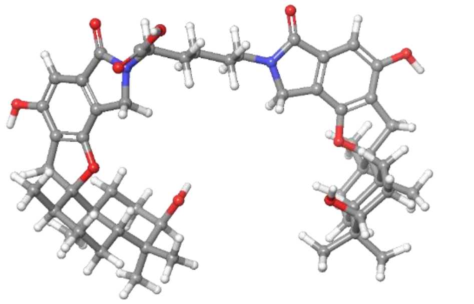	(2′′*R*, 10*R*, 10′*R*, 13*R*, 13′*R*, 14*S*, 14′*S*, 15*S*, 15′*S*, 18*R*, 18′*R*)-**2**_C2 (1.4%) 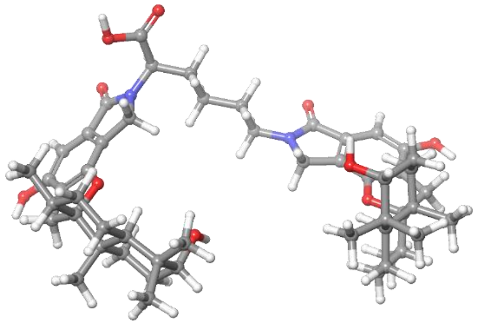
(2′′*R*, 10*R*, 10′*R*, 13*R*, 13′*R*, 14*S*, 14′*S*, 15*S*, 15′*S*, 18*R*, 18′*R*)-**2**_C3 (50.2%) 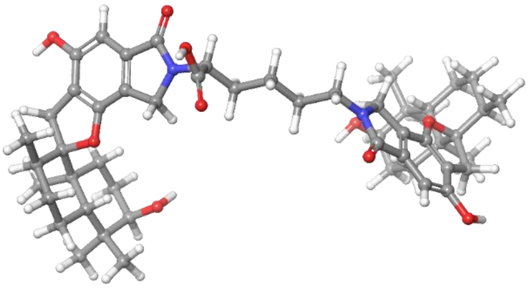	(2′′*R*, 10*R*, 10′*R*, 13*R*, 13′*R*, 14*S*, 14′*S*, 15*S*, 15′*S*, 18*R*, 18′*R*)-**2**_C4 (3.6%) 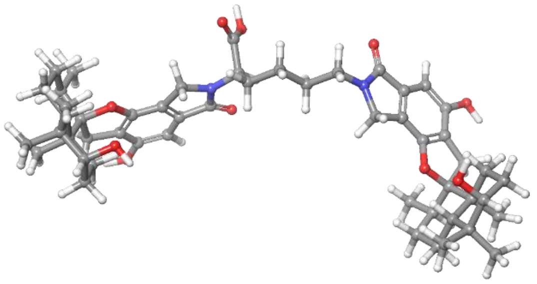

C) Structures of the low-energy conformers of (2′′*S*, 3*R*, 5*S*, 8*R*, 9*R*, 10*S*)-**3**

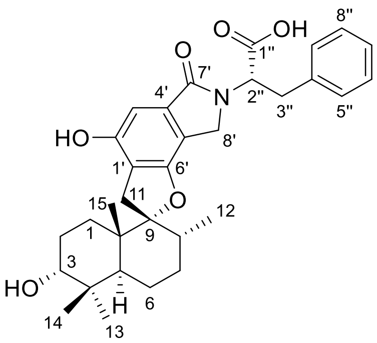

(2′′*S*, 3*R*, 5*S*, 8*R*, 9*R*, 10*S*)-**3**_C1 (73.9 %) 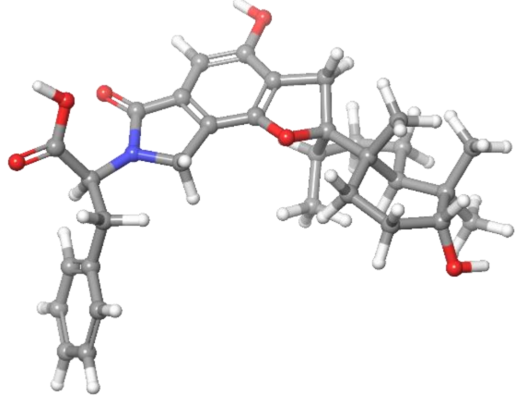	(2′′*S*, 3*R*, 5*S*, 8*R*, 9*R*, 10*S*)-**3**_C2 (24.3%) 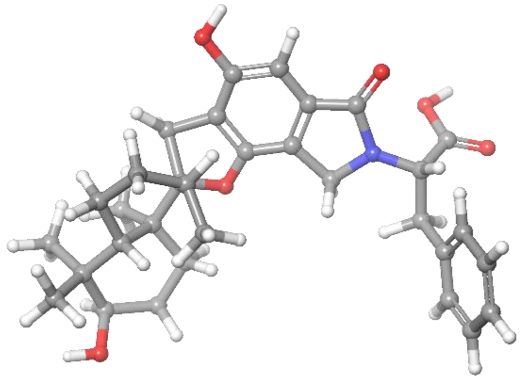
(2′′*S*, 3*R*, 5*S*, 8*R*, 9*R*, 10*S*)-**3**_C3 (1.3%) 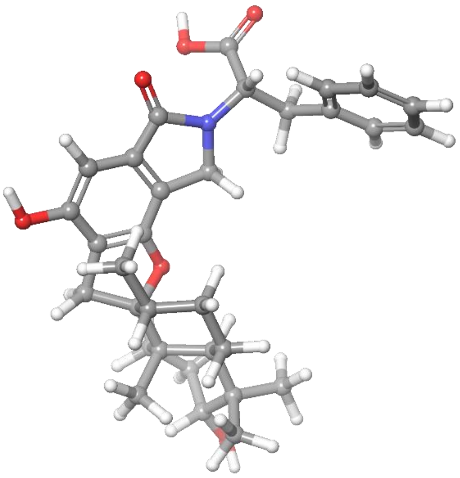	(2′′*S*, 3*R*, 5*S*, 8*R*, 9*R*, 10*S*)-**3**_C4 (0.5%) 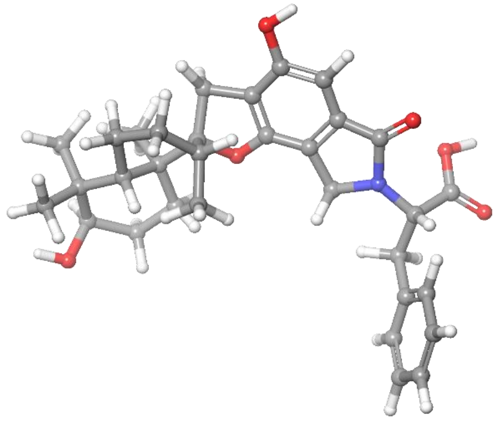

Figure S67. Structures of the low-energy B3LYP/6-311G(d,p) conformers of A) (2′′*S*, 3*R*, 5*S*, 8*R*, 9*R*, 10*S*)–**1**, B) (2′′*R*, 10*R*, 10′*R*, 13*R*, 13′*R*, 14*S*, 14′*S*, 15*S*, 15′*S*, 18*R*, 18′*R*)-**2**, and C) (2′′*S*, 3*R*, 5*S*, 8*R*, 9*R*, 10*S*)–**3** in CH_3OH (298 K and 1 atm).

3. Molecular network annotations

Table S23. Molecular network annotations (isolated compounds are highlighted in blue).

Number	Retention time (min)	*m/z*	Exact mass	Δ (ppm)	Name	Adduct
**1**	20.61	543.3170	543.3177	-1.29	stachybotrin J	$[M+H]^+$
**2**	26.41	883.5077	883.5103	-2.94	stachybocin G	$[M+H]^+$
**3**	23.43	534.2851	534.3850	0.19	stachybotrin I	$[M+H]^+$
**4**	20.03	444.2378	444.2381	-0.68	stachybotrin H	$[M+H]^+$
**5**	19.99	386.2323	386.2326	-0.78	stachybotrylactam	$[M+H]^+$
**6**	22.16	428.2418	428.2431	-3.04	stachybotrylactam acetate	$[M+H]^+$
**7**	20.69	486.2494	486.2486	1.65	2α-acetoxystachybotrylactam acetate	$[M+H]^+$
**8**	19.57	430.2583	430.2588	-1.16	stachybotramide	$[M+H]^+$
**9**	20.76	486.2853	486.2850	0.62	chartarlactam B	$[M+H]^+$
**10**	23.74	500.3019	500.3007	2.40	F1839-J	$[M+H]^+$
**17**<br>**18**	16.71<br>18.99<br>19.72	384.2179<br>384.2181<br>384.2179	384.2169<br>384.2169<br>384.2169	2.60<br>3.12<br>2.60	F1839-A<br>chartarlactam E	$[M-H_2O+H]^+$<br>$[M+H]^+$
**19**	17.44	402.2278	402.2275	0.75	chartarlactam J	$[M+H]^+$
**20**	18.95	402.2280	402.2275	1.24	chartarlactam C	$[M+H]^+$
**21**	18.86	386.2319	386.2326	-1.81	chartarlactam M	$[M+H]^+$
**22**	19.33	502.2809	502.2799	1.99	K-76-4	$[M+H]^+$
**23**	19.52	486.2847	486.2850	-0.61	stachybotrin K	$[M+H]^+$
**24**	19.76	488.2643	488.2643	0	K-76-3	$[M+H]^+$
**25**	20.33	472.2702	472.2694	1.69	stachybotrysam E	$[M+H]^+$
**26**	20.86	550.2787	550.2799	-2.18	K-76-7	$[M+H]^+$
**27**	20.93	442.2588	442.2588	0	stachybotrin D	$[M+H]^+$
**28**	20.94	400.2499	400.2482	4.25	stachybotrin E	$[M+H]^+$
**29**	21.17	458.2540	458,2537	0.65	stachybonoid E	$[M+H]^+$
**30**	22.69	486.2850	486.2850	0	stachybonoid F	$[M+H]^+$
**31**	22.76	500.3016	500.3007	1.80	stachartin C	$[M+H]^+$
**32**	23.04	373.2377	373.2373	1.07	F1839-I	$[M+H]^+$
**33**	24.93	883.5064	883.5103	-4.41	stachybocin A	$[M+H]^+$
